# FGFR2 residence in primary cilia is necessary for epithelial cell signaling

**DOI:** 10.1083/jcb.202311030

**Published:** 2025-04-22

**Authors:** Alexandru Nita, Sara P. Abraham, Eman R. Elrefaay, Bohumil Fafilek, Eliska Cizkova, Vlad Constantin Ursachi, Iva Gudernova, Adolf Koudelka, Pooja Dudeja, Tomas Gregor, Zuzana Feketova, Gustavo Rico, Katerina Svozilova, Canan Celiker, Aleksandra A. Czyrek, Tomas Barta, Lukas Trantirek, Antoni Wiedlocha, Pavel Krejci, Michaela Bosakova

**Affiliations:** 1 Department of Biology, Faculty of Medicine, Masaryk University, Brno, Czech Republic; 2 Institute of Animal Physiology and Genetics of the CAS, Brno, Czech Republic; 3 International Clinical Research Center, St. Anne’s University Hospital, Brno, Czech Republic; 4 Department of Histology and Embryology, Faculty of Medicine, Masaryk University, Brno, Czech Republic; 5 CEITEC - Central European Institute of Technology, Masaryk University, Brno, Czech Republic; 6 Department of Molecular Cell Biology, Institute for Cancer Research, Oslo University Hospital, Oslo, Norway; 7 Centre for Cancer Cell Reprograming, Faculty of Medicine, University of Oslo, Oslo, Norway

## Abstract

Primary cilium projects from cells to provide a communication platform with neighboring cells and the surrounding environment. This is ensured by the selective entry of membrane receptors and signaling molecules, producing fine-tuned and effective responses to the extracellular cues. In this study, we focused on one family of signaling molecules, the fibroblast growth factor receptors (FGFRs), their residence within cilia, and its role in FGFR signaling. We show that FGFR1 and FGFR2, but not FGFR3 and FGFR4, localize to primary cilia of the developing mouse tissues and in vitro cells. For FGFR2, we demonstrate that the ciliary residence is necessary for its signaling and expression of target morphogenic genes. We also show that the pathogenic FGFR2 variants have minimal cilium presence, which can be rescued for the p.P253R variant associated with the Apert syndrome by using the RLY-4008 kinase inhibitor. Finally, we determine the molecular regulators of FGFR2 trafficking to cilia, including IFT144, BBS1, and the conserved T^429^V^430^ motif within FGFR2.

## Teaser

Nita et al. show that the FGFR2 localizes to primary cilia of the developing mouse tissues and in vitro cells; they identify the molecular mechanism of this action, and show its critical function in FGFR2 signaling.

## Introduction

A single immotile primary cilium exists on the surface of most mammalian cells to orchestrate communication with the extracellular environment by hosting and regulating multiple signaling machinery (Wheway et al., 2018; Nachury and Mick, 2019). A growing list of studies shows that disrupted cilium architecture and signaling manifest in human disorders—the ciliopathies (Youn and Han, 2018; Quadri and Upadhyai, 2023; Silva and Cavadas, 2023; Abraham et al., 2021). The developmental ciliopathies are estimated to affect 1 in 500 individuals worldwide (Best et al., 2022), and the overall frequency is likely much higher due to the involvement of disrupted cilia in homeostasis and aging diseases including metabolic disorders (Brewer et al., 2022; Engle et al., 2021) and cancer (Jenks et al., 2018; Guan et al., 2023).

The primary cilium is built of a microtubule core named axoneme, which extends from the basal body that had matured from the mother centriole (Pedersen et al., 2008). The axoneme is sheathed by the ciliary membrane, which initiates as an extension of the cell plasma membrane but later specializes through compartmentation. This is provided by proximal structures forming the transition zone (Wei et al., 2015), which ensures selective distribution of proteins and lipids between the cytosol and the cilium (Garcia-Gonzalo and Reiter, 2017). The transmembrane proteins cross the transition zone either through the lateral diffusion (Milenkovic et al., 2009), through binding to the IFT proteins (Yang and Huang, 2020), as cargo via the BBSome (Ye et al., 2018), or by the involvement of the Golgi-to-cilium trafficking complex (Long and Huang, 2020). For some receptors, the intramolecular protein motif necessary for ciliary localization was revealed (Tam et al., 2000; Berbari et al., 2008; Loktev and Jackson, 2013; Su et al., 2015); however, no universal ciliary localization sequence has been identified.

Multiple signaling pathways were found to depend on the primary cilium (Mill et al., 2023) and can be initiated by stimulation of a receptor localized at the ciliary membrane. For example, the Hedgehog pathway receptors Patched 1 and Smoothened localize to the primary cilium, which is critical for the pathway dynamics during development (Rohatgi et al., 2007; Kim et al., 2009; Zhang and Beachy, 2023), and its failure may induce and promote multiple types of cancer (Jiang, 2022; Suchors and Kim, 2022). Other G protein–coupled receptors function within primary cilia of the sensory cells, such as the odorant receptors in the olfactory sensory neurons (Jenkins et al., 2009; Uytingco et al., 2019) or photon-sensing rhodopsin within the outer segment–modified cilia in the retina (Wang and Deretic, 2014). Another example is Notch receptors that signal from cilia to form the epidermis (Leitch et al., 2014; Ezratty et al., 2011). The Wnt coreceptor LRP6 also localizes to primary cilia to regulate the formation of neuronal precursors, kidney proximal tubules, and preadipocytes (Veland et al., 2013; Zhang et al., 2023).

The receptor tyrosine kinase (RTK) family comprises 58 transmembrane receptors that direct cell proliferation, metabolism, and cell-fate decisions of virtually all vertebrate tissues (Lemmon and Schlessinger, 2010; Robinson et al., 2000). Over 2000 RTK variants have been identified and associated with many human pathologies (Du and Lovly, 2018; Choura and Rebaï, 2011; Saraon et al., 2021; McDonell et al., 2015; https://www.ncbi.nlm.nih.gov/clinvar/), and the function of primary cilia in the RTK-driven morphogenesis and pathogenesis is beginning to emerge. The well-studied and one of the first identified ciliary RTKs, the platelet-derived growth factor receptor alpha (PDGFRα), localizes to primary cilia of fibroblast cells to control directional cell migration, cell cycle reentry, and wound healing (Schneider et al., 2005, 2010; Clement et al., 2013). Loss of ciliary PDGFRα, such as with the cancer-associated D842V variant, produces strong and prolonged signaling (Nielsen et al., 2015; Schmid et al., 2018). Other cilium-resident RTK, the insulin-like growth factor 1 receptor (IGF1R), mediates the maturation of preadipocytes (Zhu et al., 2009). Without cilia, IGF1R cannot be fully activated, leading to the reduced expression of the target genes required for adipogenesis. Similarly, loss of cilia impedes activation of the cilium-resident tropomyosin receptor kinase B (Leitch and Zaghloul, 2014). The list of RTKs found in cilia further comprises INSR (Gerdes et al., 2014), EGFR (Danilov et al., 2009; Ma et al., 2005), TIE, TEK (Teilmann and Christensen, 2005), RON (Manzanares et al., 2007), and FGFR1 (Honda et al., 2018; Evans et al., 2002).

The architecture and general function of cilia are also controlled by RTKs as well evidenced by the fibroblast growth factor receptors (FGFRs). The FGFR family consists of four members (FGFR1-4) that respond to the extracellular binding of FGF ligands by activation of intracellular signaling pathways (Ornitz et al., 1996). Deregulated FGFR signaling has been implicated not only in human developmental syndromes (Ornitz and Marie, 2019) but also in postnatal disorders including cancer (Katoh, 2019). In cultured mammalian cells, FGFR signaling was shown to regulate cilium length and interact with the Hedgehog pathway (Kunova Bosakova et al., 2018, 2019; Yuan et al., 2019). In *Xenopus* and zebrafish, manipulation with FGFR activity destabilized cilia in the organs of laterality and produced developmental defects (Neugebauer et al., 2009; Sempou et al., 2018). In tissues of patients and mice with skeletal dysplasia caused by activating FGFR3 mutation, the cilia were short and the Hedgehog signaling was inhibited (Martin et al., 2018; Kunova Bosakova et al., 2018), altogether contributing to the pathogenesis of the disorders.

Although the interaction of FGFR signaling with primary cilia has been demonstrated, the mechanisms are poorly understood. In this work, we asked which members of the FGFR family localize to primary cilia and if that controls their signaling competence. We found FGFR1 and FGFR2 in primary cilia. For FGFR2, we show that the ciliary residence is critical for its signaling and target gene expression and that it can be manipulated by targeting the ciliary trafficking regulators and the ciliary localization sequence within FGFR2.

## Results

### FGFR1 and FGFR2, but not FGFR3 and FGFR4, localize to primary cilia

**Figure 1.**
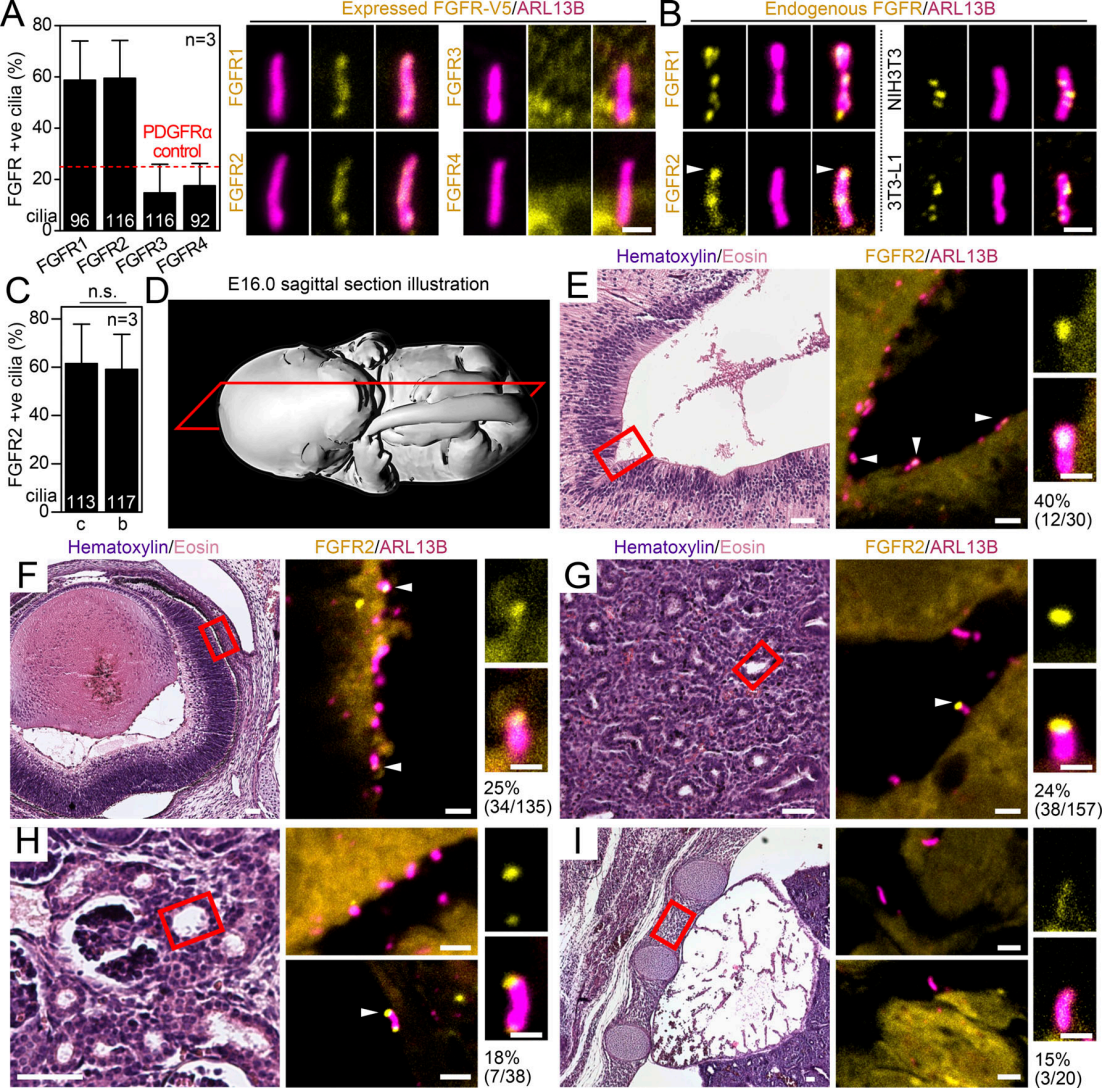
**FGFR2 localizes to primary cilia of mouse embryonic tissues and cell lines. (A)** FGFR1 and FGFR2, but not FGFR3 and FGFR4, localize to primary cilia. The IMCD3 cells were transfected with V5-tagged constructs and serum-starved for 2 days, and the expressed proteins and cilia were stained by V5 and ARL13B antibodies, respectively. The frequency of FGFR-positive cilia was plotted; the 25% threshold set by the frequency of PDGFRα-positive cilia is indicated by the red dashed line. Scale bar, 1 μm. **(B)** Endogenous FGFR1 and FGFR2 were probed by FGFR-specific antibodies in serum-starved IMCD3 (left), NIH3T3 (FGFR1), or 3T3-L1 (FGFR2) cells. The arrowhead indicates enrichment of FGFR2 in IMCD3 cilium tips. The intensity profiles of ARL13B and V5 (FGFR2) signals of the IMCD3 cilium are in Fig. S1 A. The same cilium is shown in both figures. Scale bar, 1 µm. **(C)** Frequency of ciliary localization of expressed FGFR2c and FGFR2b variants in IMCD3 cells. **(D)** Schematic presentation of the sagittal sections used for the E15.5-16.0 mouse embryos. The whole sections are shown in Fig. S1 B. **(E–H)** FGFR2 localizes to primary cilia in E15.5-16.0 mouse epithelia, including the brain ventricle (E), the retinal epithelium (F), the bronchioles (G), and the kidney collecting ducts (H). The red box in the hematoxylin/eosin-stained tissue image indicates the region which was used to scan the ARL13B and FGFR2 signals; the FGFR2 signal accumulating in the tip is indicated by the arrowhead. Detailed images of cilia with FGFR2 signal are shown as well. **(I)** FGFR2 localizes to primary cilia in E15.5-16.0 mouse intercostal mesenchyme. Scale bars, 50 μm (histology), 2 μm (IHC), and 0.5 μm (cilium detail). The numbers indicate the percentage of cilia with FGFR2 signal (% = FGFR2+ve/total cilia). The images of separate V5 and ARL13B channels and the negative control lacking the FGFR2 antibody are shown in Fig. S1, C–L, respectively. Statistical significances were calculated using Welch’s *t* test (P < 0.05); n.s., not significant. Bar plots—mean ± SEM. The *n* value indicates the number of independent experiments; the number of analyzed cilia is shown directly in the graphs.

To test the ciliary localization of FGFRs, we transiently expressed the vectors for C-terminally V5-tagged FGFR1-4 (Gudernova et al., 2017) in IMCD3 cells, an epithelial cell line that readily produces primary cilia (Deane et al., 2013; Sun et al., 2019; Rauchman et al., 1993). 4 h after transfection, the ciliation was induced by serum starvation; the cells were fixed ∼24 h later, and the FGFR-transfected cells and the cilia were visualized by immunocytochemistry using V5 and ARL13B antibodies, respectively. While ∼60% of primary cilia were positive for the presence of FGFR1-V5 and FGFR2-V5, only about 15% showed any signal for FGFR3-V5 or FGFR4-V5, which was below the threshold set by the cilium-resident PDGFRα (26.7% in PDGFRα-V5–transfected IMCD3 cells; Fig. 1 A) (Schneider et al., 2005, 2010; Clement et al., 2013; Schmid et al., 2018).

**Figure S1.**
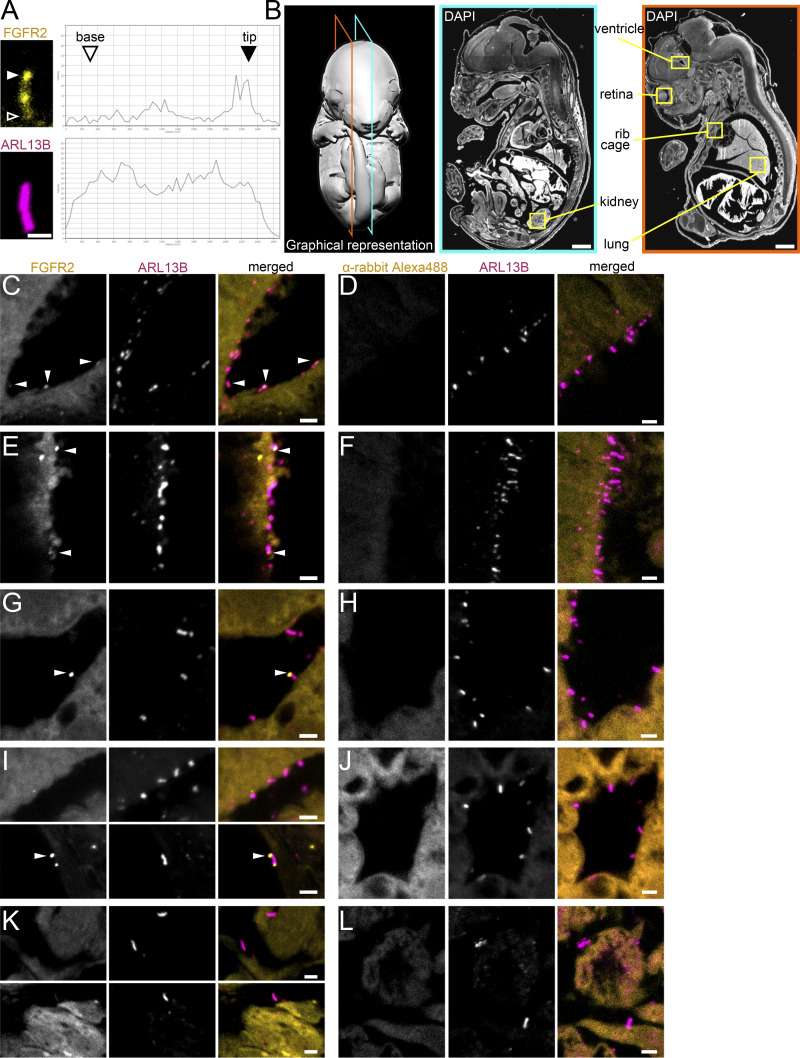
**Expanded view on FGFR2 immunohistochemistry within the mouse embryonic tissues. (A)** Analysis of the signal profile of the IMCD3 cilium from Fig. 1 B. Histogram of the FGFR2 (top) and ARL13B (bottom) signal intensity from the base to the tip of the cilium, showing accumulation of FGFR2 at the ciliary tip (full arrowhead). The IMCD3 cilium pictured is the same as in Fig. 1 B. Scale bar, 1 μm. **(B)** Schematic presentation of the sagittal sections used for immunohistochemistry of the E15.5-E16.0 mouse embryos. The E16.0 embryo model (left) and two DAPI-stained sections (middle and right) are shown, and the locations involving the kidney, brain ventricle, retina, lung, and ribs cage, used to show the FGFR2-expressing primary cilia in Fig. 1, E–I and Fig. S1, C–L, are indicated. Scale bars, 1 mm. **(C, E, G, I, and K)** Separate V5 (FGFR2) and ARL13B channels for merged images in Fig. 1, E–I. Scale bars, 2 μm. **(D, F, H, J, and L)** Negative control immunohistochemistry lacking the FGFR2 antibody. The FGFR2 signal accumulating in the tip is indicated by the arrowhead. Scale bars, 2 μm.

Next, we tested whether the endogenous FGFR1 and FGFR2 also localized to primary cilia, by utilizing antibodies specific to the endogenous proteins. For that, we used serum-starved IMCD3 (for FGFR1 and FGFR2), NIH3T3 (for FGFR1), and 3T3-L1 cells (for FGFR2). In all cell lines, we confirmed ciliary localization of the tested FGFRs; while FGFR2 was mostly observed concentrated in the distal half of the cilia (Fig. 1 B, arrowheads; Fig. S1 A), FGFR1 showed a rather homogeneous signal throughout the cilia of IMCD3 cells (Fig. 1 B). FGFR1 was previously found in the kinocilia of mechanosensory hair cells of the inner ear where it participated in formation of the apical cell polarity (Honda et al., 2018). Our data expand the pool of cell types with ciliary FGFR1 and introduce a novel cilium-resident receptor—FGFR2.

### FGFR2 is present in the primary cilia of mouse embryonic tissues

FGFR2 exists in two variants, generated by alternative splicing within the third immunoglobulin-like domain, which produces either the FGFR2b variant commonly associated with epithelial cells or the FGFR2c variant typical for mesenchyme (Orr-Urtreger et al., 1993). We expressed both variants in IMCD3 cells and found no difference in their ciliary localization (Fig. 1 C), suggesting that cilia in both epithelial and mesenchymal tissues might contain FGFR2. To test this hypothesis, we used E15.5-E16.0 mouse embryos sectioned in the sagittal plane (Fig. 1 D and Fig. S1 B). The sections were stained with FGFR2 and ARL13B antibodies, and the cilia in the previously ascribed FGFR2-expressing tissues (https://www.emouseatlas.org) were investigated for the presence of FGFR2; contiguous sections stained without the FGFR2 antibody were used as negative controls (Fig. 1, E–I and Fig. S1, C–L). We obtained FGFR2 signals in the primary cilia of the brain ventricle epithelium (Fig. 1 E), the outer retina epithelium (Fig. 1 F), the lung bronchioles (Fig. 1 G), and the kidney collecting ducts (Fig. 1 H). The majority of the signals were concentrated in the ciliary tips (Fig. 1, E–H; and Fig. S1, C, E, G, I, and K, arrowheads), similar to the IMCD3 cells (Fig. 1 B). In addition to the epithelial tissues listed above, we also analyzed the mesenchymal tissue in the intercostal region and detected FGFR2 throughout the cilia (Fig. 1 I). In contrast, we failed to see ciliary FGFR2 in the limb ectoderm and inner ear, perhaps due to a low expression level below the detection limit of the method. We cannot, however, exclude the possibility that FGFR2 does not localize to primary cilia in these tissues. Taken together, we found FGFR2 in the primary cilia of multiple mouse embryonic tissues of both epithelial and mesenchymal origins.

### FGFR2 signaling depends on primary cilia

**Figure 2.**
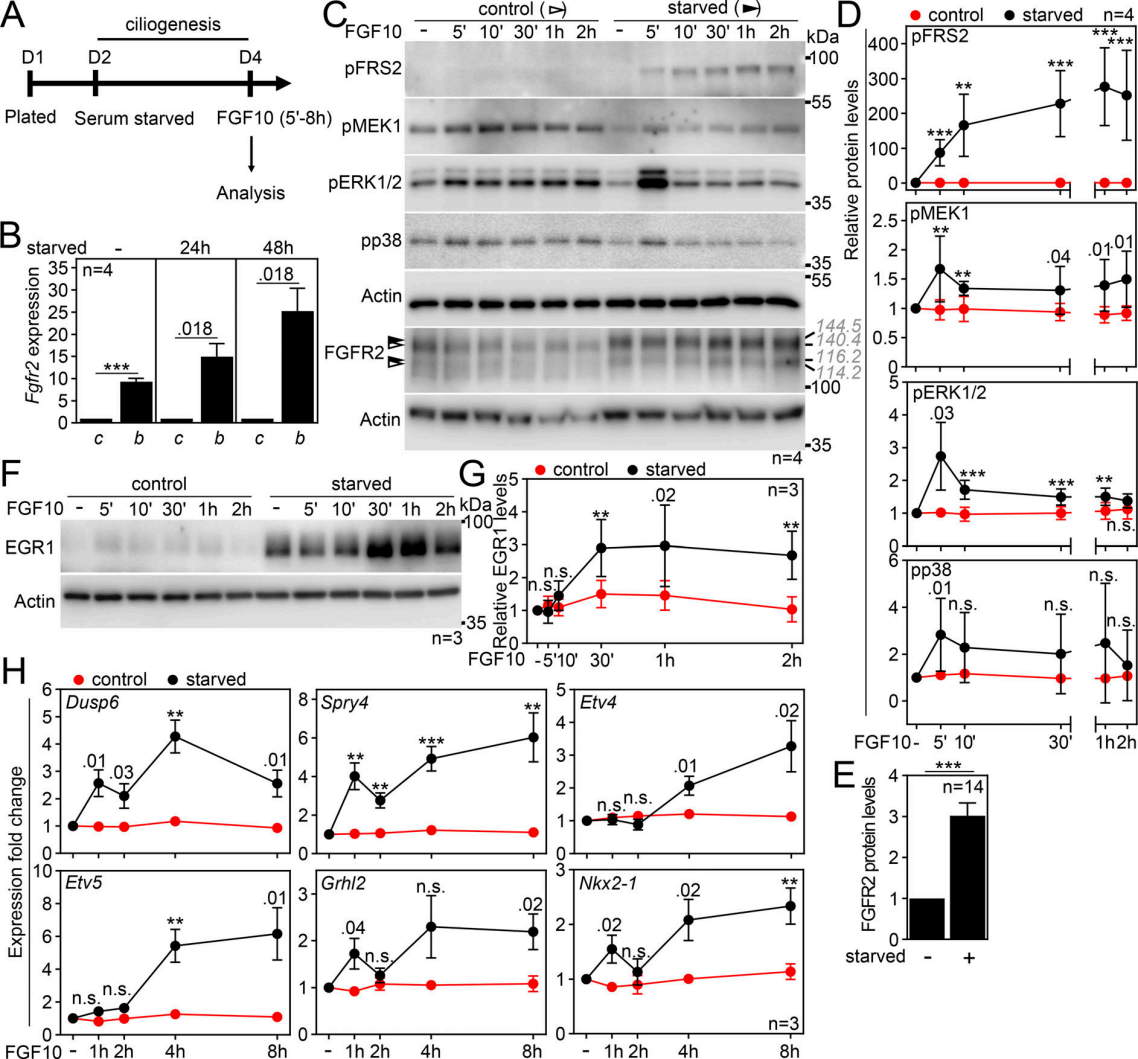
**FGFR2b signaling depends on primary cilia. (A)** Scheme of the signaling experiments. **(B)** IMCD3 cells express the epithelial FGFR2b variant. The transcript levels were analyzed by qRT-PCR after 0–48 h of serum starvation, normalized to *Ubb*, and the fold expression of *Fgfr2b* over *Fgfr2c* was plotted. **(C)** FGF10 stimulation activates FGFR2b only in serum-starved cells. IMCD3 cell lysates were immunoblotted for phosphorylated (p) FRS2, MEK1, ERK1/2, and p38, and for FGFR2; actin was used to normalize the protein levels in densitometry. The FGFR2 migration was measured, and the average values are shown in gray italics. Note the FGFR2 upshift in serum-starved cells (black arrowheads). The FGFR pathway components not found activated are in Fig. S2 A. **(D)** Densitometry of phospho-protein blots in C, normalized to actin and plotted relative to FGF-naïve cells. **(E)** Densitometry of FGFR2 blots in FGF-naïve cells in C, normalized to actin. **(F and G)** FGF10 stimulation induces the expression of EGR1 in serum-starved IMCD3 cells. Immunoblot analysis of EGR1 and actin that was used for normalization in densitometry, plotted in G as values relative to FGF-naïve cells. **(H)** Expression of FGF10 target genes in ciliated IMCD3 cells. The transcript levels were analyzed by qRT-PCR, normalized to *Ubb*, and the fold expression over FGF-naïve cells was plotted. The expression of additional tested genes for which we did not find significant upregulation is in Fig. S2 B. Statistical significances were calculated using Welch’s *t* test (P < 0.05; **P < 0.01, ***P < 0.001); n.s., not significant. Bar and line plots—mean ± SEM. The *n* value indicates the number of independent experiments. The gray italics show the actual migration of FGFR2 bands. Source data are available for this figure: SourceData F2.

**Figure S2.**
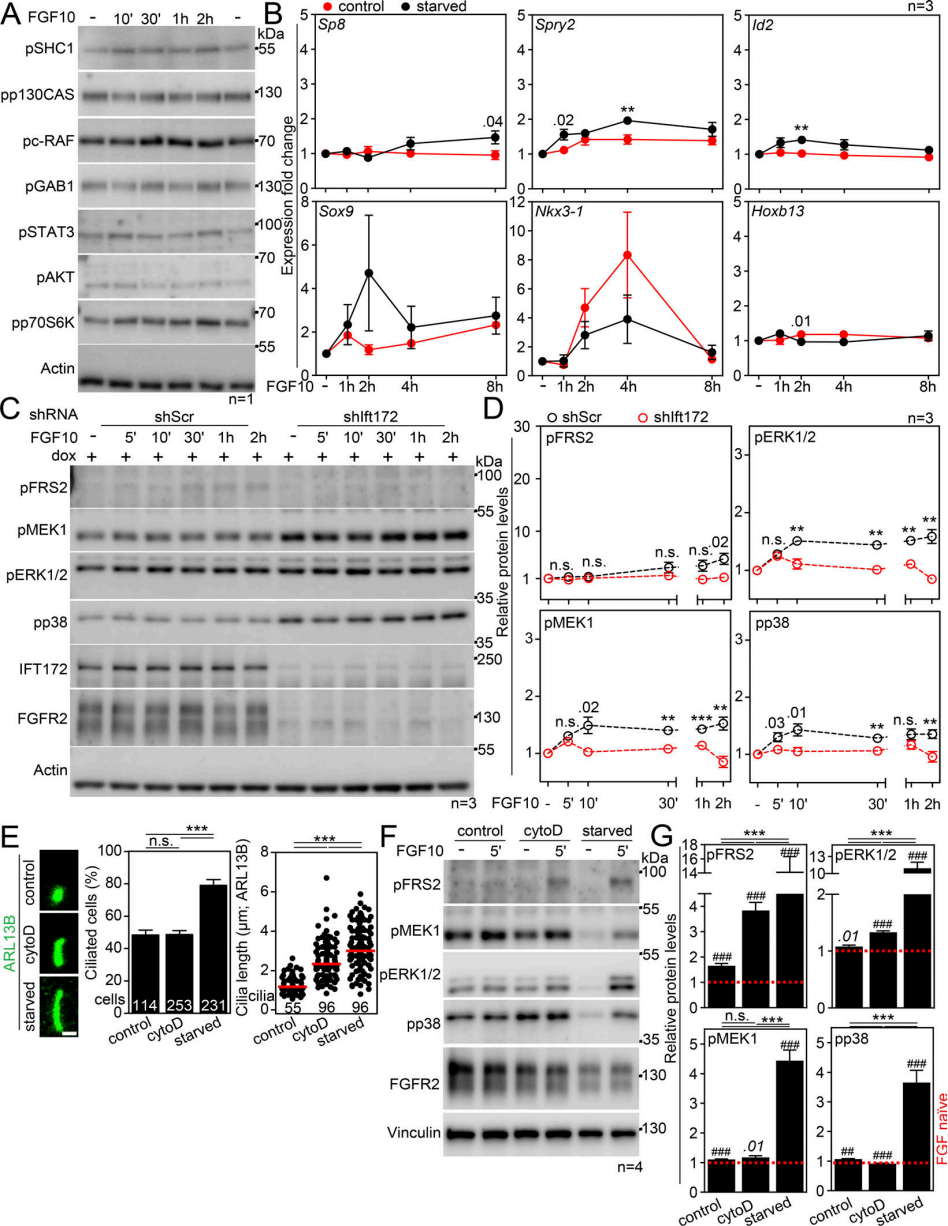
**Expanded view on cilium-dependent FGFR2 signaling. (A)** FGFR2 pathways not activated by FGF10 in serum-starved, ciliated IMCD3 cells. The IMCD3 cells were serum-starved for 2 days, treated with FGF10 for 10′-2 h, and immunoblotted for phosphorylation (p) of the indicated proteins known to function downstream of FGFRs. Actin was used as a loading control. **(B)** Genes not induced by FGF10 in serum-starved, ciliated IMCD3 cells. The IMCD3 cells were serum-starved for 2 days and then treated with FGF10 for up to 8 h; the control cells were grown in complete media. The expression of the indicated genes was normalized to *Ubb* and plotted. **(C)** Diminished signaling in non-starved IMCD3 cells expressing scrambled (Scr) or Ift172 shRNA in a dox-inducible manner. The IMCD3 cells had been dox-treated for 3 days and then treated with FGF10 for the indicated time. The cell lysates were immunoblotted for phosphorylated (p) FRS2, MEK, ERK1/2, and p38, and for FGFR2 and IFT172; actin was used to normalize the protein levels in densitometry. **(D)** Densitometry of phospho-protein blots in C, normalized to actin and plotted relative to FGF-naïve cells. **(E–G)** IMCD3 cells were treated with 200 nM cytochalasin D in complete media for 24 h to induce primary ciliary extension. **(E)** Cilia were immunostained by ARL13B antibody, and the frequency and length of primary cilia were obtained and plotted. Scale bar, 1 µm. **(F)** IMCD3 cells were treated with FGF10 for 5′ and immunoblotted for phosphorylated (p) FRS2, MEK, ERK1/2, and p38; actin was used as a loading control. **(G)** Densitometry of phospho-protein blots in F, normalized to actin and plotted relative to FGF-naïve cells. The significance indicated by italics and # is toward the FGF10-naïve cells. Statistical significances were calculated using Welch’s *t* test (P < 0.05; **/^##^P < 0.01, ***/^###^P < 0.001); n.s., not significant. Bar and line plots—mean ± SEM. Scatter plots—dots (individual cilia) and medians (red bar). The *n* value indicates the number of independent experiments; the number of analyzed cilia/cells is shown directly in the graphs. Source data are available for this figure: SourceData FS2.

Next, we asked if FGFR2 requires primary cilia to signal. The IMCD3 cells express endogenous FGFR2b (Fig. 2 B) and respond to treatment with the FGFR2b cognate ligand FGF10 (Ohuchi et al., 2000). Upon FGFR stimulation, multiple downstream pathways can get activated, including MAPK, PI3K, PLCγ, and STAT pathways (Eswarakumar et al., 2005; Turner and Grose, 2010; Ornitz and Itoh, 2015). In IMCD3 cells that had been serum-starved to produce cilia (Fig. 2 A), stimulation with FGF10 activated MAP kinase signaling, as tested by phosphorylation (p) of FRS2, MEK1, ERK1/2, and to a lesser extent also p38; no phosphorylation was detected in FGF10-treated control cells that had grown in complete medium (Fig. 2, C and D). Also, no activation of any of the tested components of the PI3 kinase, PLCγ, and STAT pathways was detected in FGF10-stimulated IMCD3 cell lysates (Fig. S2 A). This suggests that FGFR2b stimulation activates MAPK kinase signaling only in serum-starved, ciliated cells.

Activation of the MAP kinase signaling typically results in gene expression changes (Kolch, 2000; Murphy et al., 2004). Stimulation of serum-starved, ciliated IMCD3 cells with FGF10 produced upregulation of the early growth response 1 protein, which was detectable after 30 min of stimulation (Fig. 2, F and G) (Gudernova et al., 2017). Therefore, we decided to test whether other FGF10/FGFR2b target genes, implicated in the morphogenesis of multiple organs and tissues, including the kidney, lung, thymus, and skeleton (Huang et al., 2005; Michos et al., 2010; Jones et al., 2019; Kawakami et al., 2004; Revest et al., 2001), also became expressed. We observed rapid upregulation at 1 h of FGF10 stimulation for *Dusp6* and *Spry4*, the two established negative feedback regulators of FGFR signaling (Li et al., 2007; Katoh and Katoh, 2006) (Fig. 2 H). Other FGF10-induced genes included *Etv4*, *Etv5*, *Grhl2*, and *Nkx2-1*, known to function during early mouse lung and kidney morphogenesis (Jones et al., 2019; Michos et al., 2010) (Fig. 2 H). Expression analysis of six additional genes revealed minimal to no significant response to FGF10 (Fig. S2 B), perhaps due to their limited function in IMCD3 cells. In summary, we showed that FGF10 induces the expression of target morphogenic genes only in serum-starved, ciliated cells.

**Figure 3.**
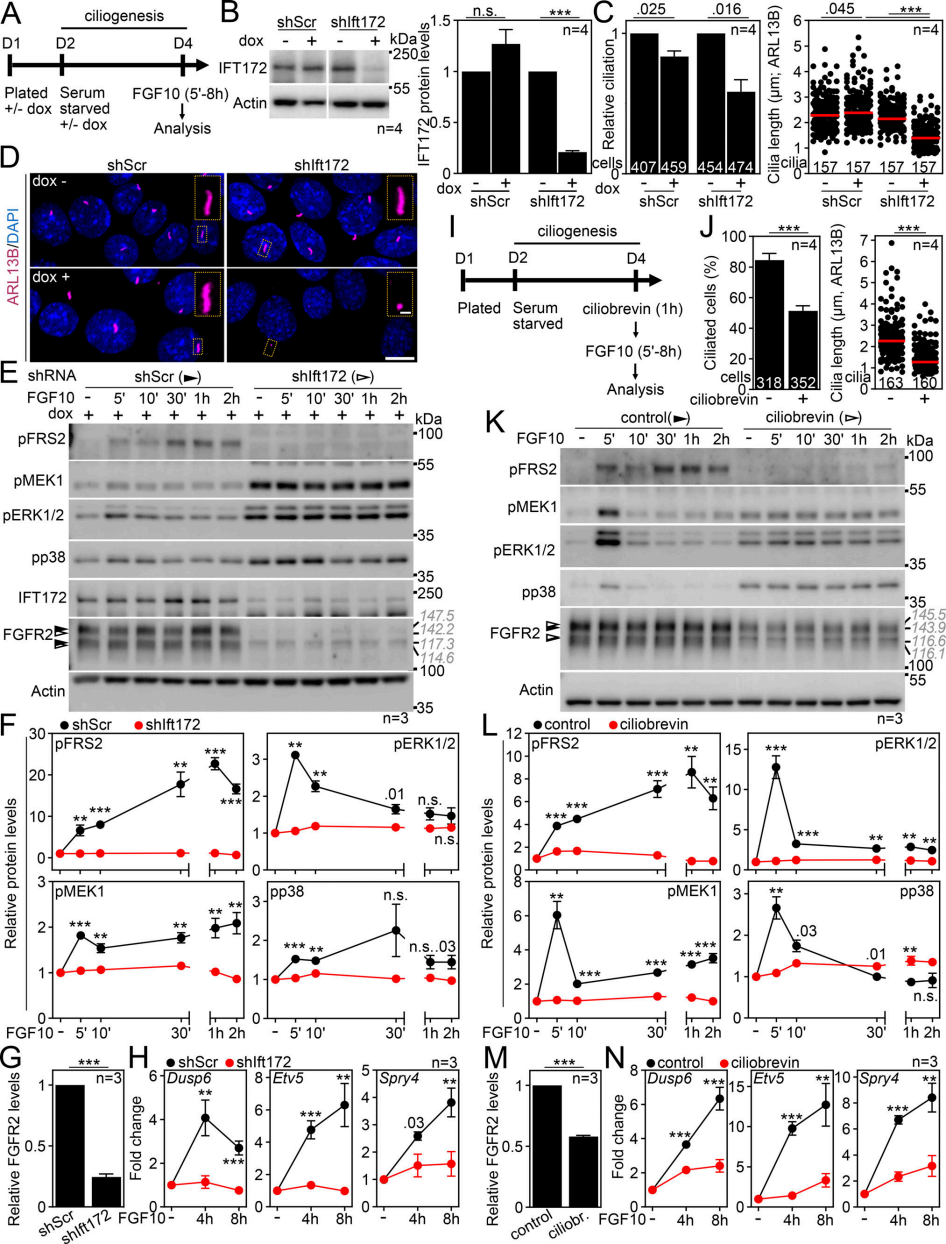
**Deciliation produces loss of FGFR2b signaling. (A)** Scheme of the signaling experiments involving dox-inducible shRNA in IMCD3 cells. **(B)** IFT172 levels in cells after 3 days of shRNA expression (dox^+^ cells); cells expressing scrambled shRNA (shScr cells) were used as controls. Actin was used for the normalization of IFT172 densitometry, which was plotted. **(C)** Inhibition of cilium frequency and length in shIft172 dox^+^ cells. The numbers of analyzed cells and cilia are indicated. **(D)** Representative field views and cilium details of shScr and shIft172 cells ± dox. Scale bars, 10 µm (cells) and 1 µm (cilia). **(E and F)** FGF10 stimulation does not activate FGFR2b signaling in cells that had lost cilia due to IFT172 knockdown. IMCD3 cell lysates were immunoblotted for phosphorylated (p) FRS2, MEK1, ERK1/2, and p38, and for FGFR2 and IFT172; actin was used to normalize the protein levels in densitometry. The FGFR2 migration was measured, and the average values are shown in gray italics. The diminished signaling response of non-starved cells is shown in Fig. S2, C and D. **(F)** Densitometry of phospho-protein blots in E, normalized to actin and plotted relative to FGF-naïve cells. **(G)** Densitometry of FGFR2 blots in FGF-naïve cells in E, normalized to actin. **(H)** Expression of FGF10 target genes. The transcript levels were analyzed by qRT-PCR, normalized to *Gapdh*, and the fold expression of *Fgfr2* over FGF-naïve cells was plotted. **(I)** Scheme of the signaling experiments involving ciliobrevin treatment in IMCD3 cells. **(J)** Inhibition of cilium frequency and length in ciliobrevin cells. The numbers of analyzed cells and cilia are indicated. **(K)** Loss of FGF10 response in cells treated with ciliobrevin. The protein lysates were analyzed as in E. The FGFR2 migration was measured, and the average values are shown in gray italics. **(L)** Densitometry of phospho-protein blots in K, normalized to actin and plotted relative to FGF-naïve cells. **(M)** Densitometry of FGFR2 blots in FGF-naïve cells in K, normalized to actin. **(N)** Expression of FGF10 target genes. The transcript levels were analyzed by qRT-PCR, normalized to *Gapdh*, and the fold expression of *Fgfr2b* over FGF-naïve cells was plotted. Statistical significances were calculated using Welch’s *t* test (P < 0.05; **P < 0.01, ***P < 0.001); n.s., not significant. Bar and line plots—mean ± SEM. Scatter plots—dots (individual cilia) and medians (red bar). The *n* value indicates the number of independent experiments; the number of analyzed cilia/cells is shown directly in the graphs. The gray italics show the actual migration of FGFR2 bands. Source data are available for this figure: SourceData F3.

To exclude the possibility of acquired FGFR2 signaling being a consequence of serum starvation rather than the presence of primary cilia, we produced IMCD3 cells in which ciliogenesis diminished due to the doxycycline (dox)-dependent expression of shRNA targeting IFT172 (Wang et al., 2018) (Fig. 3, A–D). In serum-starved, cilium-ablated cells (shIft172 + dox), the FGF10 stimulation produced hardly any signaling response (Fig. 3, E and F), while the scrambled control cells (shScr + dox) signaled normally as judged by pFRS2, pMEK1, pERK1/2, and pp38 levels; the target gene expression was also diminished in the cilium-ablated cells (Fig. 3 H). Without serum starvation, none of the cells responded to FGF10 (Fig. S2, C and D). Alternatively, we treated cells with ciliobrevin A, which produces rapid deciliation by inhibiting the ciliary dynein motor (Firestone et al., 2012). After 1 h with ciliobrevin, the ciliation dropped by 39% (P = 0.0008; Welch’s *t* test), and the remaining cilia were shorter by 39% on average (P < 0.0001, Welch’s *t* test), resulting in a debilitated primary cilium (Fig. 3, I and J). When stimulated with FGF10, minimal signaling response and target gene expression were detected in the ciliobrevin-treated cells (Fig. 3, K–N). Finally, we tested whether forced cilium production in complete media induces sensitivity of IMCD3 cells to FGF10 stimulation. For that, we used cytochalasin D, which promotes ciliogenesis by inhibiting actin filaments (Pan et al., 2007). A 24-h cytochalasin treatment induced axoneme maturation and induced partial signaling response in the non-starved FGF10-treated IMCD3 cells (Fig. S2, E–G). Taken together, we demonstrate that FGFR2 requires primary cilium to signal.

### Production of primary cilium stabilizes FGFR2

**Figure S3.**
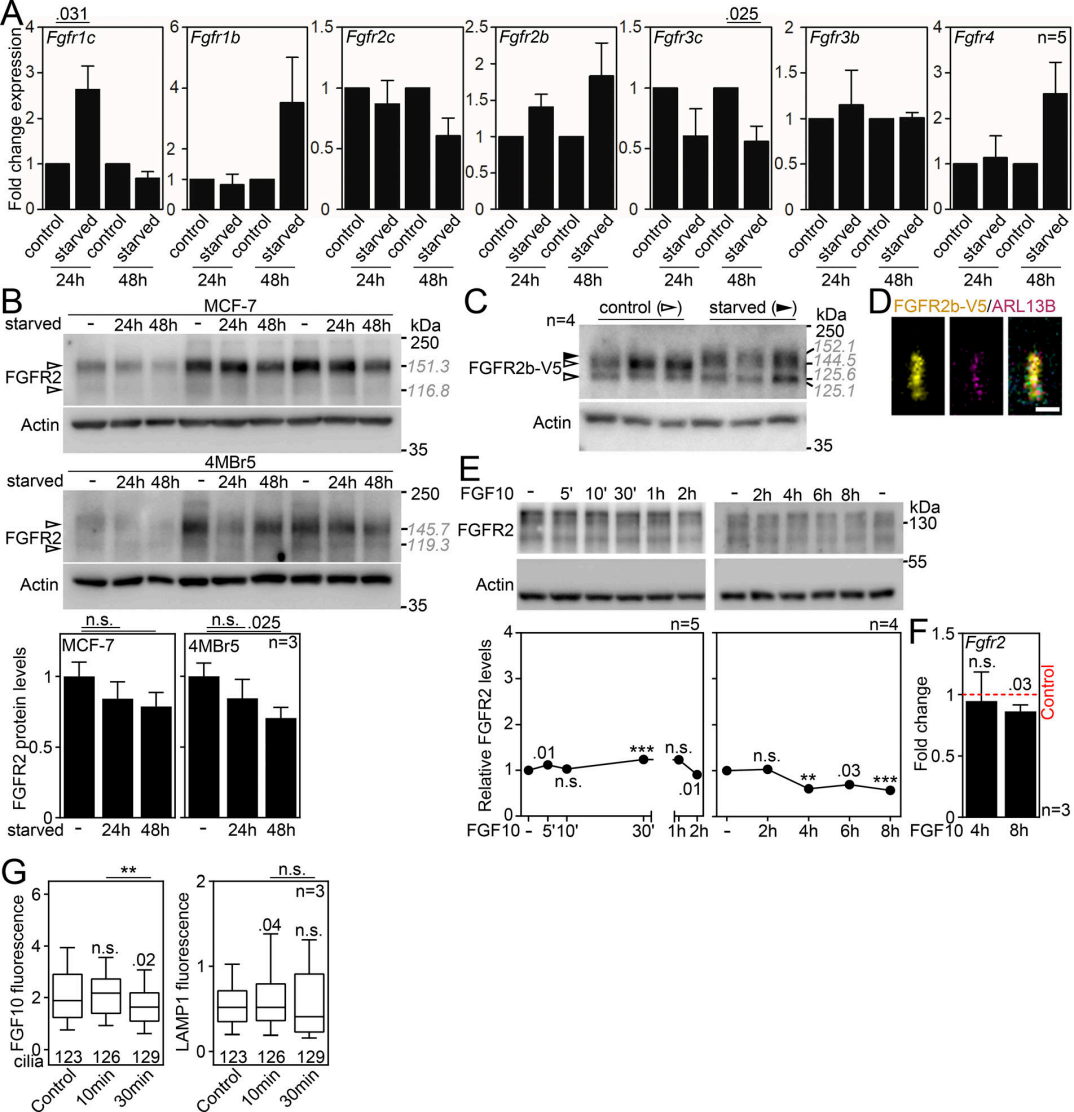
**Expanded view on FGFR expression and FGFR2 gel migration. (A)** qRT-PCR analysis of *Fgfr1-4*, including their b and c variants, in IMCD3 cells that had been serum-starved for 24 or 48 h. *Ubb* was used for normalization; data are presented relative to non-starved control cells. **(B)** Western blot of MCF-7 and 4MBr5 cells that had been serum-starved for 24 or 48 h. The actual migration of FGFR2 is shown in gray italics (kDa). Actin was used for FGFR2 normalization in densitometry; data are presented relative to non-starved control cells. The three replicates represent independent biological experiments. **(C)** Western blot of IMCD3 cells stably transfected with FGFR2b-V5, showing migration upshift of FGFR2 in 48-h serum-starved cells (black arrowhead). The actual migration of FGFR2 is shown in gray italics. **(D)** Confocal image of the 48-h serum-starved FGFR2b-V5 cells, showing signal for FGFR2b in the ARL13B-labeled primary cilium. Scale bar, 1 μm. **(E)** Western blot of IMCD3 cells that had been serum-starved for 48 h and treated with FGF10 for the indicated time. Actin was used for FGFR2 normalization in densitometry; data are presented relative to non-treated cells. **(F)** qRT-PCR analysis of *Fgfr2* in serum-starved IMCD3 cells that have been treated with FGF10 for 4 or 8 h. *Ubb* was used for normalization; data are presented relative to non-treated control cells (red dashed line). **(G)** Fluorescence intensity analysis of the observed cilium-bound FGF10-DyLight 550 and cilium-localized LAMP1 from Fig. 4 D. To note is the weaker FGF10 signal at 30′, suggesting cilium exit of FGFR2/FGF10. Moreover, 10 min after the ligand stimulation, LAMP1 accumulates in the primary cilium. The significance is displayed toward the non-treated control cells. Statistical significances were calculated using Welch’s *t* test (P < 0.05; **P < 0.01, ***P < 0.001); n.s., not significant. Bar and line plots—mean ± SEM. Box and whiskers—min-max 10–90%. The *n* value indicates the number of independent experiments; the number of analyzed cilia is shown directly in the graphs. The gray italics show the actual migration of FGFR2 bands. Source data are available for this figure: SourceData FS3.

We noticed that the serum-starved IMCD3 cells expressed about three times more FGFR2 than the non-starved control cells (P < 0.0001, Welch’s *t* test; Fig. 2, C and E), which was unlikely to be solely due to increased expression since the *Fgfr2b* transcript levels did not change significantly (Fig. S3 A). Notably, the FGFR2b accumulation appeared cilium-dependent since serum starvation of two epithelial cell lines that cannot produce primary cilia, MCF-7 and 4MBr-5, did not increase the FGFR2 levels (Fig. S3 B). This suggests that FGFR2 accumulates in serum-starved IMCD3 cells due to compartmentation within the cilium and formation of the cilium-specific FGFR2 signaling complexes that are known to reduce receptor degradation (Partridge et al., 2004; Lenferink et al., 1998; Reddi et al., 2007). It is of note that the ciliary membrane has a unique lipid composition compared with the general plasma membrane (Nechipurenko, 2020). In IMCD3-like canine MDCK cells, the ciliary membrane is enriched with sphingolipids including those found in lipid rafts (Janich and Corbeil, 2007; He et al., 2012), and additional detergent-resistant lipid microdomains condense around the ciliary base (Vieira et al., 2006). Such microdomains provide a platform where RTKs accumulate and interact with their downstream signaling effectors (Levental and Veatch, 2016; Roy and Patra, 2023). For example, a specific lipid composition is required for GRB2 to bind FGFR2 to stabilize its homodimer conformation (Rohwedder et al., 2021), which protects FGFR from degradation (Ahmed et al., 2010, 2013; Lin et al., 2012) and allows for extensive FGFR autophosphorylation (Ahmed et al., 2010). This can be evidenced by the migration upshift of FGFR2 within the acrylamide gels, which was apparent in serum-starved IMCD3 cells (∼4.1 kDa for the upper, membranous FGFR2 band with P = 0.005, Welch’s *t* test; Fig. 2 C). A similar upshift was present in serum-starved, ciliated IMCD3 cells stably expressing V5-tagged FGFR2 (∼7.6 kDa with P = 0.001, Welch’s *t* test; Fig. S3 C), and importantly, it was absent in non-ciliated MCF-7 and 4MBr-5 cells (Fig. S3 B). At the same time, the cilium-ablated shIft172 cells showed accelerated FGFR2 migration (∼5.3 kDa with P = 0.0004, Welch’s *t* test) (Fig. 3 E), which was similar to the non-starved IMCD3 cells (Fig. 2 C). In summary, the presence of primary cilia stabilizes FGFR2, which correlates with sensitivity to ligand stimulation.

### FGF10 activates FGFR2/MAP kinase signaling within the primary cilium

**Figure 4.**
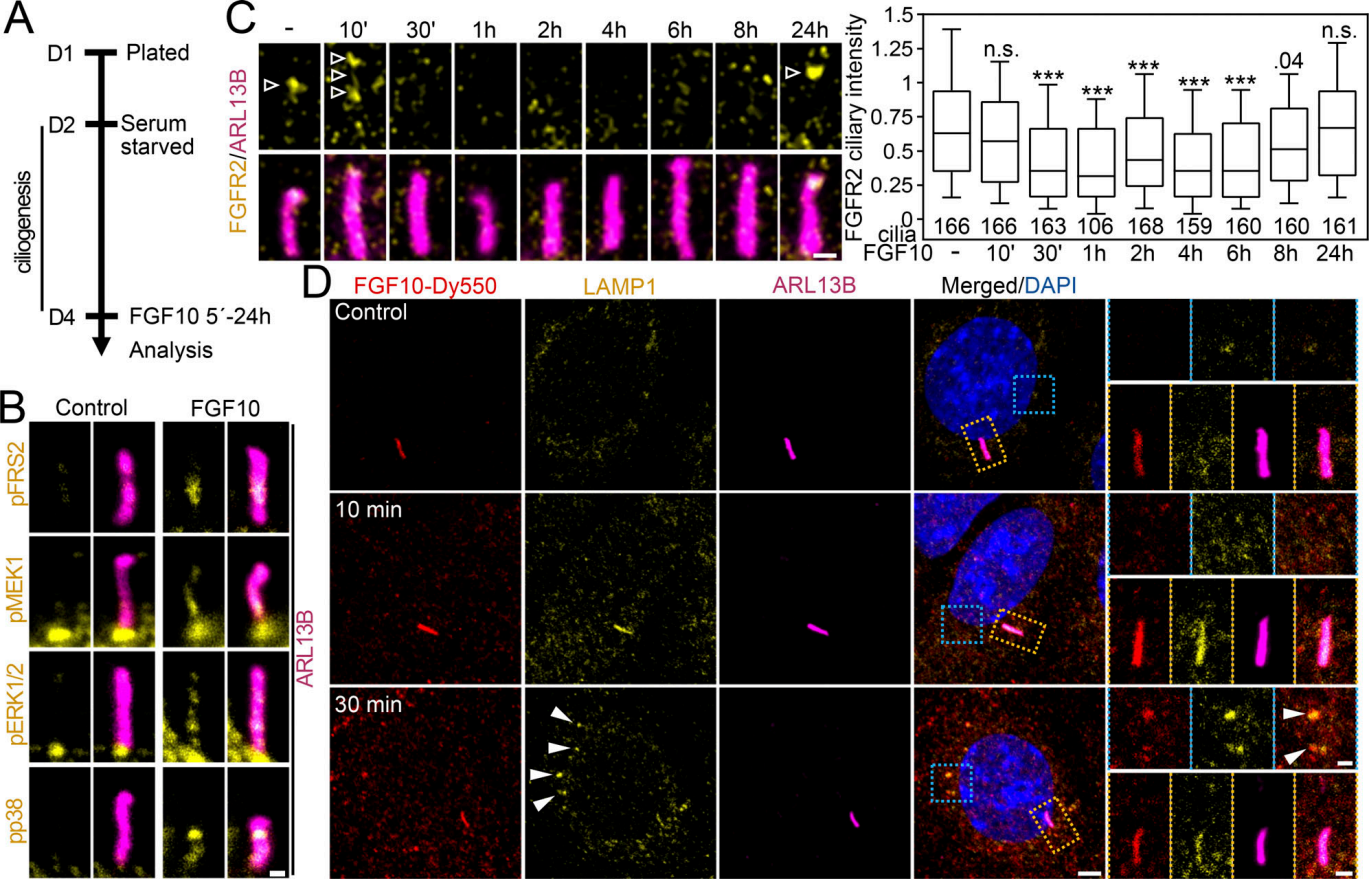
**FGF10 activates FGFR2b/MAP kinase signaling within the primary cilium. (A)** Scheme of the confocal experiments involving FGFR2 dynamics and signaling. **(B)** Ciliary localization of MAP kinase signaling proteins. IMCD3 cells were serum-starved for 2 days, treated with 50 ng/ml FGF10 for 5′, and subjected to immunocytochemistry for ARL13B (cilia) and the phosphorylated (p) FRS2, MEK1, ERK1/2, and p38. Note the changes in intensity and localization of p-proteins within cilia upon FGF10 stimulation. Scale bar, 0.5 μm. **(C)** Dynamics of ciliary FGFR2 upon FGF10 stimulation, and accumulation of the receptor signal at the distal part of the cilium (arrowheads). IMCD3 cells were serum-starved for 2 days, treated with FGF10 for the indicated time, and subjected to FGFR2 and ARL13B immunocytochemistry. The ciliary FGFR2 intensity was measured and plotted. The significance is displayed toward the non-treated control. **(D)** IMCD3 cells were serum-starved for 2 days and then treated with 300 ng/ml FGF10-DyLight 550 (FGF10-Dy550) and 1 µg/ml heparin on ice for 30 min (control cells). The cells were then incubated at 37°C for 10 or 30 min and subjected to immunocytochemistry for LAMP1 and ARL13B. Note the ciliary LAMP1 signal peaking at 10′ and the perinuclear puncta with overlapping LAMP1 and FGF10-Dy550 at 30′. Scale bar, 2 μm (cells) and 1 μm (insets). Statistical significances were calculated using Welch’s *t* test (P < 0.05; **P < 0.01, ***P < 0.001); n.s., not significant. Box and whiskers—min-max 10–90%. The *n* value indicates the number of independent experiments; the number of analyzed cilia is shown directly in the graph.

Having established the importance of primary cilia for FGFR2 signaling, we aimed to learn about the spatial dynamics of the pathway within cilia. Since the MAP kinase activation peaked at 5′ upon FGF10 stimulation (Fig. 2, C and D), we used the same setup to colocalize pFRS2, pMEK1, pERK1/2, and pp38 within the ciliary region. Without FGF10 stimulation, virtually no pFRS2 was detected in cilia, corresponding to its absence in the western blot (Fig. 2, C and D; and Fig. 4, A and B). FGF10 treatment produced a clear pFRS2 signal at the proximal/central part of the cilium, which was coupled with the ciliary appearance of pMEK1, pERK1/2, and pp38 (Fig. 4 B). Taken together, FGFR2 responds to FGF10 stimulation by activation of the MAP kinase pathway within primary cilia.

Upon binding of the FGF ligand, FGFRs typically internalize and undergo either degradation or recycling to the cell surface (Kostas et al., 2018; Haugsten et al., 2005, 2011, 2016). For FGFR2, the final destination seems ambiguous and dependent on the stimulating ligand, the receptor localization, or the cell type (Samad et al., 2024). Therefore, we asked if FGFR2 leaves the cilium when the pathway gets activated if it gets internalized, and how it becomes sorted within cells. We started by treating the serum-starved IMCD3 cells with FGF10 for up to 24 h and analyzed the ciliary FGFR2 intensity. We found that as early as 30′ after FGF10 stimulation, the median ciliary FGFR2 levels dropped by 44% (with P < 0.0001, Welch’s *t* test), stayed low until 6 h, and completely recovered 24 h after initial treatment (Fig. 4 C). The decreased ciliary intensity was not due to receptor degradation since the total cellular FGFR2 levels did not change much within the 8-h period after FGF10 treatment (Fig. S3 E). Similarly, the restoration of ciliary FGFR2 was not due to increased *Fgfr2* gene expression (Fig. S3 F). FGFR2b stimulation by FGF10 has been shown to favor the recycling pathway of the receptor rather than degradation (Francavilla et al., 2013). Our data further support this observation and suggest that FGFR2b undergoes ciliary exit when activated by FGF10.

To improve sensitivity of the subcellular tracking of FGFR2b, we took advantage of FGF10 that had been covalently labeled with DyLight 550 (FGF10-Dy550). The IMCD3 cells were incubated with FGF10-Dy550 on ice for 30 min to saturate the FGFR2. At this stage, the FGF10-Dy550 was present exclusively at the cilium (Fig. 4 D). Then, the cells were provided warm media and transferred to 37°C to induce signaling. After 30 min, the ciliary FGF10-Dy550 signal was substantially weaker, suggesting exit from the cilium (Fig. S3 G). Since FGF ligands typically internalize together with the bound FGFR (Haugsten et al., 2005), we followed the FGF10-Dy550 in cells and noticed vesicular structures appearing at the perinuclear region. These vesicles colocalized with LAMP1, a glycoprotein that marks late endosomes in which FGFR is found during its turnover in cells (Sørensen et al., 2006). Taken together, our data show that ciliary FGFR2 undergoes internalization through the LAMP1 compartments.

### Diminished ciliary localization of the disease-associated FGFR2 variants

**Figure 5.**
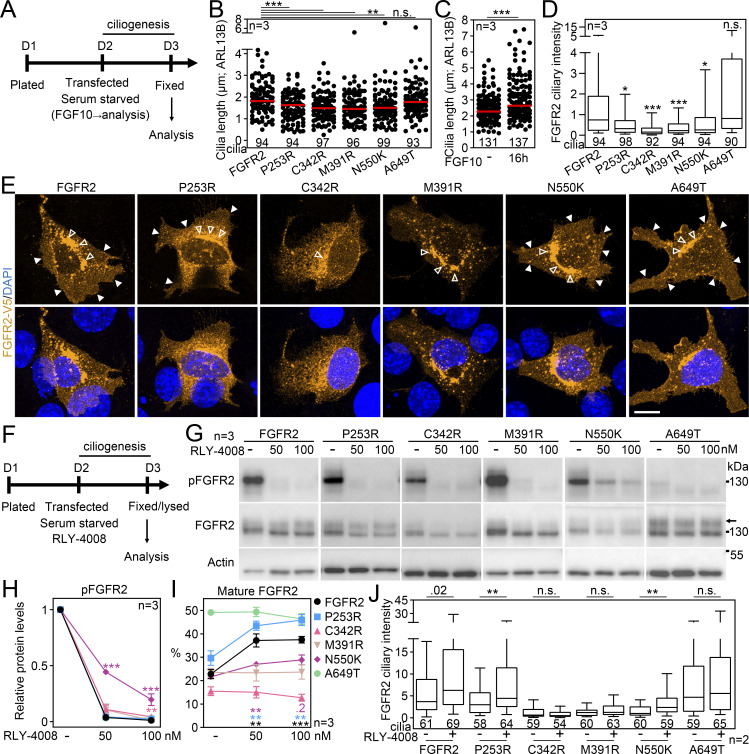
**Disease-associated activating FGFR2 variant shows poor ciliary localization. (A)** Scheme of experiments involving the expression of disease-associated FGFR2 variants in IMCD3 cells. **(B)** Activating FGFR2 variants shorten primary cilia. The fixed IMCD3 cells were stained by V5 (FGFR2) and ARL13B antibodies, and the cilium length was measured and plotted. The cilium frequency was also tested and is plotted in Fig. S4 A. **(C)** Serum-starved IMCD3 cells were treated with FGF10 for 16 h, and the cilium length was obtained. **(D)** Activating FGFR2 variants show low ciliary amounts. The ciliary intensity of FGFR2 in transfected IMCD3 cells was measured and plotted. The significance is displayed toward the wild-type FGFR2. **(E)** Subcellular localization of FGFR2 variants. White arrowheads—cell membrane signal; empty arrowhead—perinuclear signal. Scale bar, 10 μm. **(F)** Scheme of experiments involving the expression of disease-associated FGFR2 variants and RLY-4008 inhibition. **(G–I)** Maturation and activity of FGFR2 variants in the presence of RLY-4008. **(G)** The cell lysates were immunoblotted for FGFR2 transactivation using the pFGFR^Y653/4^ antibody. Actin was used as a loading control. The arrow indicates the mature FGFR2 with slower gel migration. **(H)** pFGFR densitometry, normalized to total FGFR2 and plotted relative to RLY-4008–naïve cells. The significance is displayed toward the wild-type FGFR2. **(I)** Effect of RLY-4008 on the maturation of FGFR2 variants. The percentage of mature FGFR2 was obtained after FGFR2 densitometry of blots in (G; mature/total) and plotted relative to RLY-4008–naïve cells. The significance is displayed toward the RLY-4008–naïve cells. The effect of RLY-4008 on cilium length, ciliation percentage and subcellular localization of FGFR2 is shown in Fig. S4, B–D. **(J)** Ciliary intensity of FGFR2 variants after RLY-4008 kinase inhibition. Statistical significances were calculated using Welch’s *t* test (P < 0.05; **P < 0.01, ***P < 0.001); n.s., not significant. Line plots—mean ± SEM. Scatter plots—dots (individual cilia) and medians (red bar). Box and whiskers—min-max 10–90%. The *n* value indicates the number of independent experiments; the number of analyzed cilia is shown directly in the graphs. Source data are available for this figure: SourceData F5.

**Figure S4.**
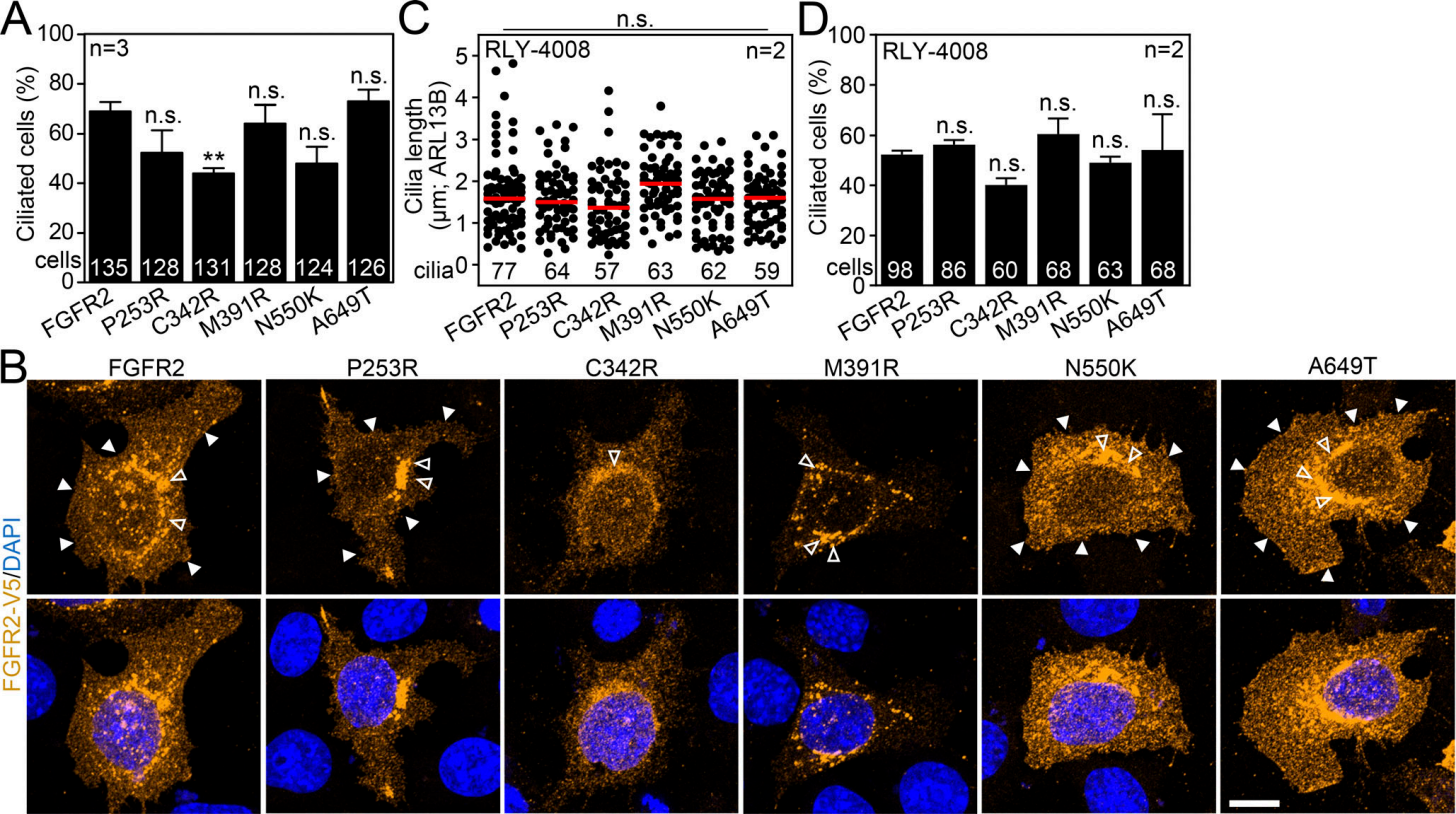
**Expanded view on** **activating FGFR2 variants** **. (A)** IMCD3 cells were fixed and stained by V5 and ARL13B antibodies, and the ciliary frequency of transfected cells was calculated and plotted. **(B)** Subcellular localization of FGFR2 variants in the presence of 100 nM RLY-4008. White arrowheads—cell membrane signal; empty arrowhead—perinuclear signal. Scale bar, 10 μm. **(C)** Immunocytochemistry analysis showing rescue of the cilium lengths of the cells transfected with the FGFR2 variants and treated with RLY-4008. **(D)** Percentage of ciliated cells is not affected in IMCD3s expressing the FGFR2 variants and treated with RLY-4008. Statistical significances were calculated using Welch’s *t* test (P < 0.05; **P < 0.01, ***P < 0.001); n.s., not significant. The significance is displayed toward the wild-type FGFR2. Bar plots—mean ± SEM. Scatter plots—dots (individual cilia) and medians (red bar). The *n* value indicates the number of independent experiments; the number of analyzed cilia/cells is shown directly in the graphs.

Having established that FGFR2 leaves the primary cilium after ligand binding and activation, we asked if the same holds true for the activating FGFR2 variants coupled with human disorders. For that, we selected the cancer-associated FGFR2 p.N550K (Stehbens et al., 2018), and also the Apert syndrome p.P253R (Wilkie et al., 1995), the Crouzon syndrome p.C342R (Reardon et al., 1994), the bent bone dysplasia p.M391R (Merrill et al., 2012), and the kinase-dead p.A649T (Shams et al., 2007). The variants carrying an activating mutation, but not the kinase-dead FGFR2 A649T, shortened primary cilia by 10–24% (P < 0.01, Welch’s *t* test) and produced a mild inhibition of ciliogenesis with some variants (Fig. 5, A and B; and Fig. S4 A). This was in contrast to FGF10 stimulation, which produced cilium elongation by 16% on average (P < 0.0001, Welch’s *t* test; Fig. 5 C). This is in line with the previously published data where ligand-mediated FGFR activation produced longer primary cilia in cultured mammalian cells (Kunova Bosakova et al., 2018). In contrast, the pathological FGFR3 activity in the human skeletal syndromes was associated with shortening of primary cilia (Kunova Bosakova et al., 2018; Martin et al., 2018), similar to our observation with the FGFR2 variants (Fig. 5 B). This suggests that the transient, ligand-mediated FGFR activation has a differing impact on cilium length than the sustained, mutation-driven activity. Part of the mechanism of how FGFR3 regulates cilium length is through extraciliary interaction with the evolutionary-conserved ciliogenesis-associated kinase CILK1 (Chaya et al., 2014; Berman et al., 2003; Burghoorn et al., 2007; Kunova Bosakova et al., 2019; Paige Taylor et al., 2016). FGFR2, however, does not interact with CILK1 (Kunova Bosakova et al., 2019) and may rather connect with cilia through residency and dependent signaling (Figs. 1, 2, 3, and 4).

We found all disease-associated FGFR2 variants to have limited amounts in primary cilia (Fig. 5 D). Since altered subcellular localization has been reported with the N550K, P253R, and M391R variants (Stehbens et al., 2018; Ahmed et al., 2008; Merrill et al., 2012), we tested whether that is recapitulated in our system and potentially prohibits the ciliary entry. The overexpressed wild-type FGFR2 showed a widespread signal at the cell membrane, and a significant perinuclear signal was present as well (Fig. 5 E), likely due to the forced expression where proteins tend to accumulate in the endoplasmic reticulum (Wagner et al., 2006). This pattern was similar in the P253R, N550K, and A649T variants; however, the M391R and C342R variants showed inhibited plasma membrane signals, and the latter was also present throughout the cytosol (Fig. 5 E). When the transfected cells were treated with the highly selective FGFR2 inhibitor RLY-4008 (Subbiah et al., 2023), the FGFR2 activity completely diminished for all variants but p.N550K at 100 nM concentration (Fig. 5, F–H). The kinase inhibition greatly improved the maturation of wild-type FGFR2 and the p.P253R variant and partly also p.N550K (Fig. 5 G, arrow, and Fig. 5 I), which was coupled with their enhanced ciliary localization (Fig. 5 J). The maturation of p.C342R and p.M391R FGFR2 and their ciliary localization did not change with complete kinase inhibition by RLY-4008 (Fig. 5, F–J and Fig. S4), suggesting that their altered subcellular localization stems from other mechanisms. FGFR2 M391R was shown to localize to the nucleus of transfected BaF3 cells (Merrill et al., 2012); however, we did not observe any nuclear signals in transfected IMCD3 cells in which the FGFR2 M391R signal was mostly concentrated in perinuclear vesicles (Fig. 5 E), even after RLY-4008 kinase inhibition (Fig. S4 B). It is possible that the M391R mutation, which changes the conformation of the transmembrane domain, prevents membrane integration of FGFR2 (Merrill et al., 2012). Whether the disrupted disulfide bond formation within FGFR2 C342R (Robertson et al., 1998) contributes to impaired receptor maturation and ciliary localization is unknown.

### FGFR2 ciliary trafficking requires IFT144, BBS1, and GRK2

**Figure 6.**
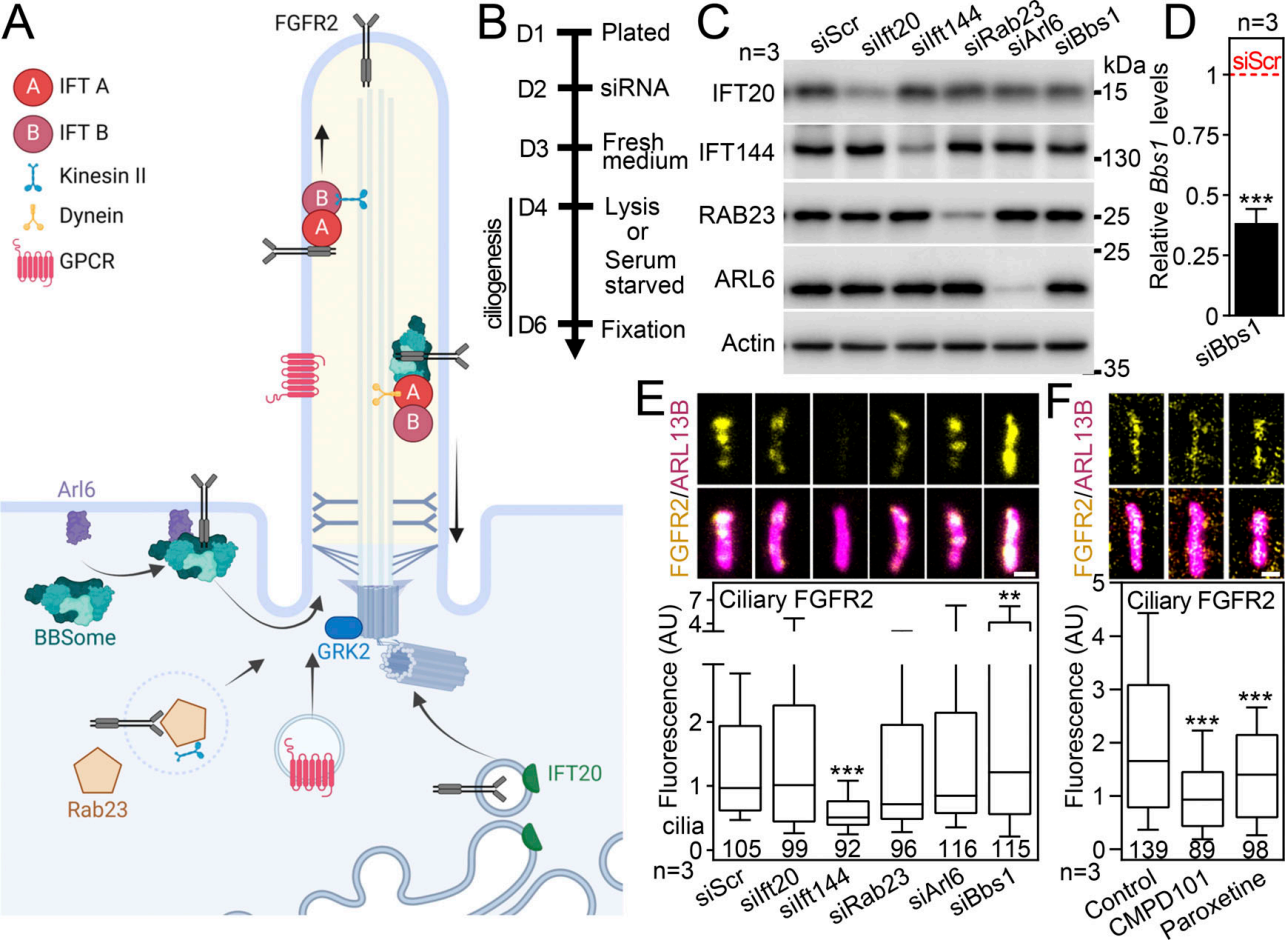
**FGFR2 ciliary trafficking requires IFT144, BBS1 and GRK2. (A)** Schematic presentation of proteins with previously ascribed function in ciliary trafficking of transmembrane receptors. **(B)** Scheme of the siRNA experiments. **(C)** Western blot of cells transfected with siRNA, showing downregulation of IFT20, IFT144, RAB23, and ARL6, respectively; actin was used as a loading control and for normalization of densitometry (Fig. S5 A). The effect on cilium frequency and length is in Fig. S5 B. **(D)***Bbs1* transcript level in cells transfected with Bbs1 siRNA, compared with the scrambled control (siScr, red dashed line), and normalized using *Gapdh* expression. **(E)** Effect of siRNA expression on ciliary FGFR2 amounts, obtained after FGFR2 and ARL13B immunocytochemistry. Scale bar, 1 μm. The significance is displayed toward the siScr cells. Total cellular FGFR2 levels are in Fig. S5 C. The effect of siRNA expression on ciliary levels of FGFR1 and FGFR2 in IMCD3, NIH3T3, and 3T3-L1 cells is in Fig. S5, E–J. **(F)** Effect of GRK2 inhibitors CMPD101 (10 µM) and paroxetine (2 µM) on ciliary FGFR2 amounts, after serum starvation in the presence of the respective inhibitors. Scale bar, 1 μm. The significance is displayed toward the non-treated cells. The total cellular FGFR2 levels are in Fig. S5 D. Statistical significances were calculated using Welch’s *t* test (P < 0.05; **P < 0.01, ***P < 0.001); n.s., not significant. Bar plot—mean ± SEM. Box and whiskers—min-max 10–90%. The *n* value indicates the number of independent experiments; the number of analyzed cilia is shown directly in the graphs. Source data are available for this figure: SourceData F6.

**Figure S5.**
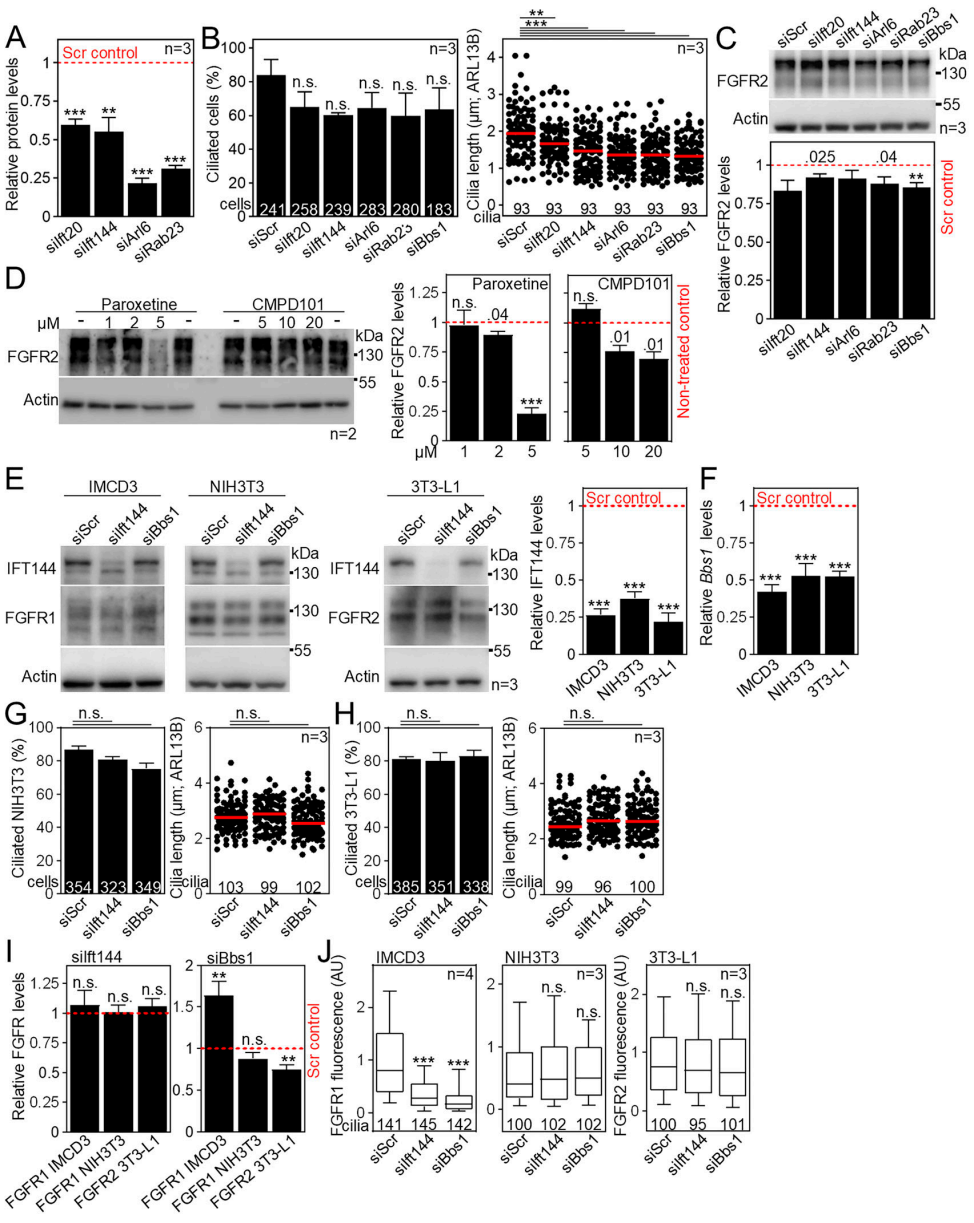
**Expanded view on molecular regulators of ciliary FGFR trafficking. (A)** Densitometry of western blots in Fig. 6 C. **(B)** IMCD3 cells were transfected with siRNA, fixed after 2-day serum starvation, the cilia were immunostained by ARL13B antibody, and the frequency and length of primary cilia were obtained and plotted. The significance is displayed toward the siScr cells. **(C)** FGFR2 levels in IMCD3 cells transfected with siRNA. Actin was used for quantification using densitometry where the data are presented relative to the Scrambled (Scr) siRNA control cells (red dashed line). **(D)** IMCD3 cells were serum-starved for 48 h in the presence of GRK2 inhibitors CMPD101 or paroxetine, and the cell lysates were then western-blotted for FGFR2 and actin for loading controls and normalization in densitometry. The FGFR2 levels were plotted relative to non-treated cells (red dashed line). **(E)** Western blot of cells transfected with siRNA, showing downregulation of IFT144 and the FGFR1 and FGFR2 levels in IMCD3, NIH3T3, and 3T3-L1 cells, respectively. Actin was used as a loading control and for normalization of densitometry where the data are presented relative to the Scrambled (Scr) siRNA control cells (red dashed line). **(F)***Bbs1* transcript level in cells transfected with Bbs1 siRNA, compared with the siScr control (red dashed line), and normalized using *Ubb* expression. **(G and H)** Cilia were immunostained by ARL13B antibody, and the frequency and length of primary cilia in siRNA-transfected NIH3T3 (G) and 3T3-L1 (H) cells were obtained and plotted. **(I)** Densitometry of FGFR1 and FGFR2 blots from Fig. S5 E, presented relative to the Scrambled (Scr) siRNA control cells (red dashed line). **(J)** Effect of siRNA expression on ciliary FGFR1 (IMCD3 and NIH3T3 cells) and FGFR2 (3T3-L1 cells) levels, compared with siScr cells. The ciliary FGFR1 and FGFR2 signals were obtained after FGFR1/2 and ARL13B immunocytochemistry. Statistical significances were calculated using Welch’s *t* test (P < 0.05; **P < 0.01, ***P < 0.001); n.s., not significant. Bar plots—mean ± SEM. Scatter plots—dots (individual cilia) and medians (red bar). Box and whiskers—min-max 10–90%. The *n* value indicates the number of independent experiments; the number of analyzed cilia/cells is shown directly in the graphs.

To identify the molecular regulators of FGFR2 ciliary localization, we targeted the expression of five proteins previously associated with ciliary import of the membrane molecules, namely, IFT20 (Follit et al., 2006; Jonassen et al., 2008), IFT144 (Behal et al., 2012; Mukhopadhyay et al., 2010), ARL6 (Jin et al., 2010), RAB23 (Boehlke et al., 2010), and BBS1 (Starks et al., 2015; Singh et al., 2020), using siRNA (Fig. 6, A–D and Fig. S5 A). Downregulation of these proteins had only a mild effect on cilium frequency and length in IMCD3 cells (Fig. S5 B). Significant changes were found for ciliary FGFR2 intensity that dropped in IFT144 siRNA cells by 61% (P < 0.001, Welch’s *t* test) and increased in BBS1 siRNA cells by 70% on average (P = 0.0022, Welch’s *t* test), when compared to control scrambled siRNA cells (Fig. 6 E and Fig. S5 C). We also analyzed ciliary FGFR2 in cells treated with the GRK2 kinase inhibitors paroxetine and CMPD101 (Thal et al., 2012; Pusapati et al., 2018), since GRK2 activity was shown to regulate membrane trafficking and activity of several RTKs, including the cilium-resident IGF1R, INSR, and EGFR (Métayé et al., 2005; Cipolletta et al., 2009; Hupfeld and Olefsky, 2007; Hildreth et al., 2004), as well as ciliary trafficking of Smoothened (Bosakova et al., 2020; Pusapati et al., 2018). At concentrations that did not strongly affect the FGFR2 expression levels (Fig. S5 D), the GRK2 inhibition reduced the ciliary FGFR2 levels by 15–43% on average (P < 0.001, Welch’s *t* test; Fig. 6 F). Collectively, our data indicate mechanisms that FGFR2 uses to localize in cilia. Mechanistically, ARL6 enables lateral diffusion of proteins from the plasma membrane toward the cilium through enriched rafts, which are loaded on the BBSome (Jin et al., 2010). The BBSome then acts as a cargo adaptor for the IFT machinery through interaction with IFT144 and mediates cargo exit from the primary cilium (Wei et al., 2012). Our data suggest that FGFR2 does not arrive at the cilium through lateral diffusion, and instead implicate an interaction with BBSome that appears to regulate both ciliary import (less signal in IFT144 siRNA cells) and export (more signal in BBS1 siRNA cells) of FGFR2 (Fig. 6 E).

Next, we asked if these mechanisms also function in ciliary trafficking of FGFR1 and in other cell types. Similar as for FGFR2, downregulation of IFT144 reduced amounts of ciliary FGFR1 in IMCD3 cells (Fig. S5, E–J); however, BBS1 downregulation reduced ciliary FGFR1 as well, which was in contrast to FGFR2 (Fig. 6 E versus Fig. S5 J). Neither IFT144 nor BBS1 seemed to regulate ciliary levels of FGFR1 and FGFR2 in the mesenchymal cell lines NIH3T3 and 3T3-L1, respectively. While we do not have an explanation for this phenomenon, it is possible that different mechanisms apply to the ciliary trafficking of FGFRs in the mesenchymal (NIH3T3 and 3T3-L1) and epithelial (IMCD3) cells. Taken together, we showed that IFT144 promotes FGFR1 and FGFR2 trafficking to the primary cilia of epithelial IMCD3 cells.

### FGFR2 requires its juxtamembrane T^429^V^430^ motif to enter the primary cilium

**Figure 7.**
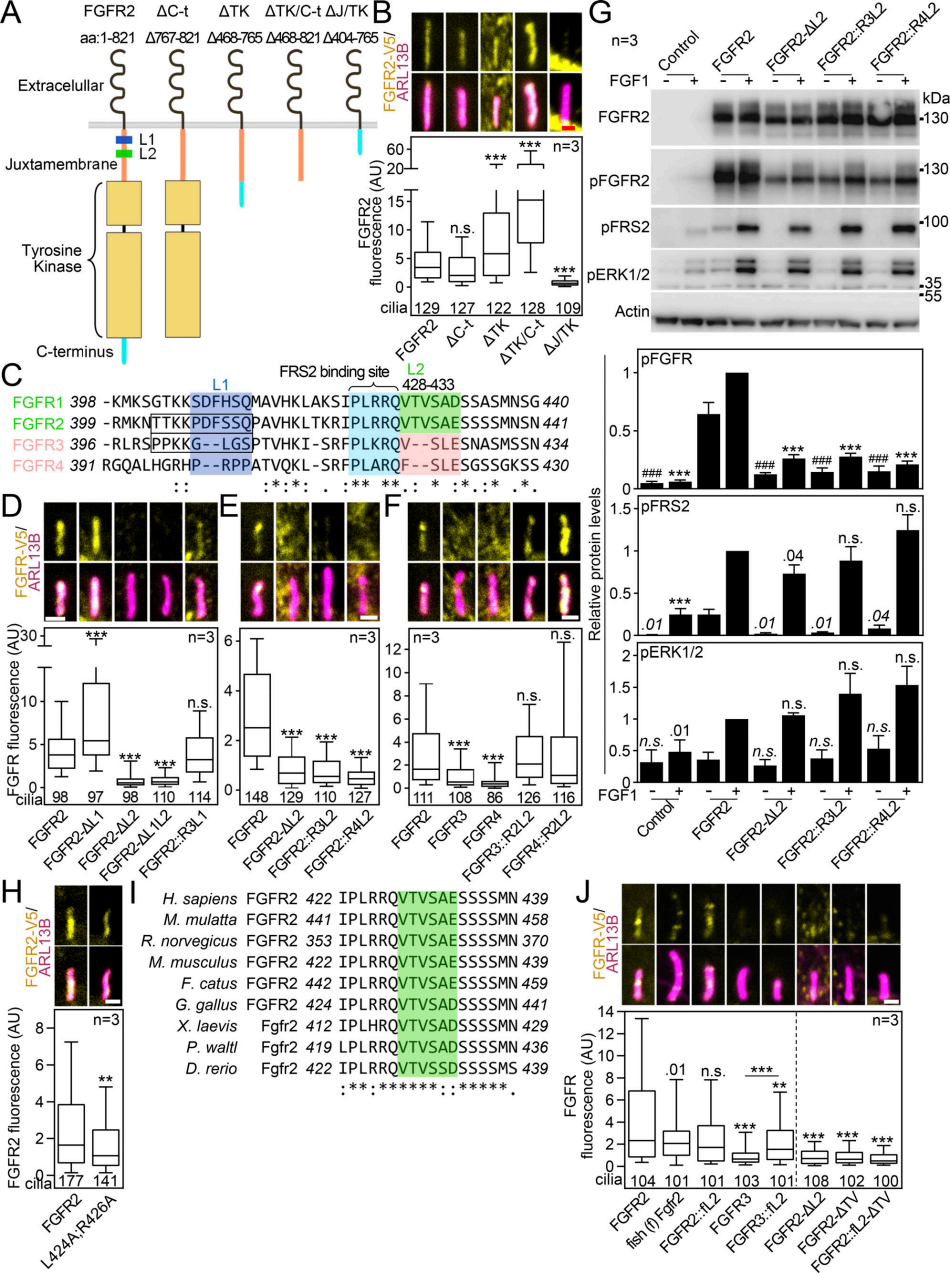
**FGFR2 requires its juxtamembrane T** ^**429**^ **V** ^**430**^ **motif to enter the primary cilium. (A)** Schematic presentation of FGFR2 truncation constructs; the FGFR2 domains, the L1 and L2 sequences, and the deleted amino acids are indicated; the expression is shown in Fig. S6 A. **(B)** FGFR2 constructs were expressed in IMCD3; the cells were serum-starved for 24 h, and the ciliary FGFR2 intensity was measured and plotted. Note the loss of ciliary signal for ΔJ/TK. The number of analyzed cilia is indicated. Scale bar, 1 μm. The significance is displayed toward the wild-type FGFR2. **(C)** Amino acid sequence alignment of the N-terminal part of the juxtamembrane region of human FGFR1-4; the conservation is indicated (*fully conserved residue; : strongly conserved residue, i.e., >0.5 in Gonnet PAM 250 matrix; . weakly conserved residue, i.e., ≤0.5 in Gonnet PAM 250 matrix). The L1 and L2 motifs are highlighted, as well as the FRS2 binding site. The box marks the extended L1 motif targeted in the constructs shown in D to preserve stability of the protein. **(D–F)** L2 sequence is critical for ciliary entry of FGFRs. Various FGFR constructs comprising the L1 and L2 sequences were expressed in IMCD3 cells and analyzed as in B. The number of analyzed cilia is indicated. The sequences and expression are shown in Fig. S6, B and C. Scale bar, 1 μm. **(D)** L1 motif does not regulate the ciliary localization of FGFR2. **(E)** L2 motif is critical for ciliary localization of FGFR2. The effect of L2 placement into other locations within FGFR2 is shown in Fig. S6, D–F. **(F)** Substitution by the FGFR2 L2 sequence rescues ciliary localization of FGFR3 and FGFR4. **(G)** Disruption of L2 sequence inhibits FGFR2 activation. 293T cells were transfected with FGFR2 vectors that varied in the sequence of the L2 motif. The next day, the cells were treated with FGF1, and the FGFR2 activity was evaluated by phosphorylation (p) of FGFR, FRS2, and ERK1/2. Actin was used for normalization in densitometry, and the data were plotted relative to FGF-treated FGFR2. The significance is indicated by italics and # toward the non-treated FGFR2, and by regular text and * toward the FGF1-treated FGFR2. **(H)** The FGFR2-L424A;R426A variant was expressed and analyzed as in B. The number of analyzed cilia is indicated. The expression is shown in Fig. S6 G. Scale bar, 1 μm. **(I)** Amino acid sequence alignment of the FGFR2 region comprising the L2 motif (green) in vertebrates ranging from *H. sapiens* to *D. rerio*, showing the high level of conservation. **(J)** Identification of the T^429^V^430^ motif within L2, responsible for the FGFR2 ciliary localization. IMCD3 cells were transfected and analyzed as in B. The number of analyzed cilia is indicated. Scale bar, 1 μm. The significance is displayed toward the wild-type FGFR2. The sequences and expression are shown in Fig. S6, H and I. Statistical significances were calculated using Welch’s *t* test (P < 0.05; **P < 0.01, ### or ***P < 0.001); n.s., not significant. Bar plots—mean ± SEM. Box and whiskers—min-max 10–90%. The *n* value indicates the number of independent experiments; the number of analyzed cilia is shown directly in the graphs. Source data are available for this figure: SourceData F7.

**Figure S6.**
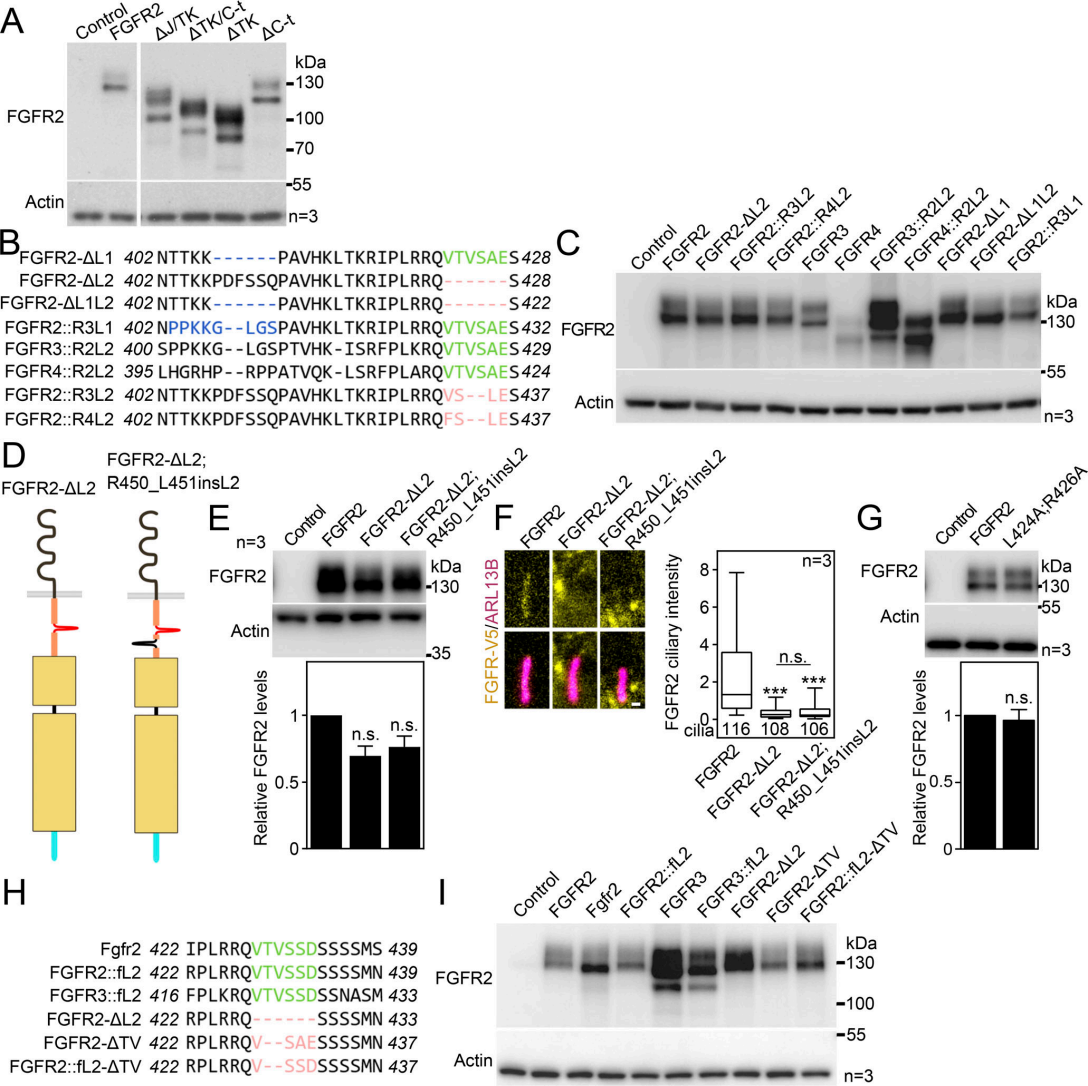
**Expanded view on the FGFR constructs tested for ciliary localization. (A)** Western blot showing expression and maturation of the FGFR2 truncation constructs from Fig. 7, A and B, using 1-day transfection of IMCD3 cells. Actin was used as a loading control. **(B)** Nucleotide sequence within the region of interest in FGFR constructs used in Fig. 7, D–F. **(C)** Western blot showing expression and maturation of the FGFR constructs from Fig. 7, D–F, using 1-day transfection of IMCD3 cells. Actin was used as a loading control. **(D)** Schematic presentation of construct with the L2 motif placed into another location within the juxtamembrane region. **(E)** Western blot showing expression and maturation of the FGFR2 constructs from Fig. S6 D, using 1-day transfection of IMCD3 cells. Actin was used as a loading control and normalization in densitometry. **(F)** Ciliary FGFR2 signals were obtained after FGFR2 and ARL13B immunocytochemistry of transfected IMCD3 cells and plotted. **(G)** Western blot showing the expression of the FGFR2-L424A;R426A variant from Fig. 7 H, using 1-day transfection of IMCD3 cells; actin was used for normalization in densitometry. **(H)** Nucleotide sequence within the region of interest in FGFR2 constructs used in Fig. 7 J. **(I)** Western blot showing the expression and maturation of the FGFR constructs from Fig. 7 J, using 1-day transfection of IMCD3 cells. Actin was used as a loading control. Statistical significances were calculated using Welch’s *t* test (P < 0.05; **P < 0.01, ***P < 0.001); n.s., not significant. Bar plots—mean ± SEM. Box and whiskers—min-max 10–90%. The significance is displayed toward the wild-type FGFR2. The *n* value indicates the number of independent experiments; the number of analyzed cilia is shown directly in the graph. Source data are available for this figure: SourceData FS6.

Next, we searched for a ciliary localization motif within FGFR2. We generated a series of C-terminally V5-tagged FGFR2 truncation mutants (Fig. 7 A), which we expressed in IMCD3 cells and quantified their capacity to localize to the primary cilia. The C-terminal region is important for FGFR interaction with downstream adaptors (Mohammadi et al., 1991; Ahmed et al., 2010; Lonic et al., 2008); however, we found no difference in FGFR2 ciliary localization when the C terminus was removed (ΔC-t; Fig. 7 B). Next, we deleted the whole tyrosine kinase domain (ΔTK); this FGFR2 variant showed enhanced accumulation within cilia (2.3-fold on average; P < 0.0001, Welch’s *t* test), which was boosted by additional removal of C-t (ΔTK/C-t; P < 0.0001, Welch’s *t* test). It is possible that removal of the bulky tyrosine kinase domain impedes FGFR2 ciliary exit orchestrated by BBSome (Fig. 6 E). Importantly, deletion of the juxtamembrane region completely abolished ciliary FGFR2 localization (ΔJ/TK; P < 0.0001, Welch’s *t* test; Fig. 7 B), which was not due to poor expression or stability since all FGFR2 variants expressed at similar amounts (Fig. S6 A).

The four members of the FGFR family differ in their ciliary localization capacity. While we found FGFR1 and FGFR2 to enter the primary cilia of IMCD3 cells, FGFR3 and FGFR4 did not (Fig. 1 A). When we aligned the juxtamembrane sequences of FGFR1-4, we identified two motifs consisting of six amino acids (denoted as L1 and L2; Fig. 7 C) that were identical or highly similar in FGFR1 and FGFR2 but were not conserved in FGFR3 and FGFR4. Removal of L2 (FGFR2-ΔL2) but not L1 (FGFR2-ΔL1) completely abolished ciliary localization of FGFR2 (Fig. 7 D; and Fig. S6, B and C). Similarly, the replacement of the L1 sequence in FGFR2 by the corresponding FGFR3 sequence (FGFR2::R3L1; extended by upstream four amino acids to preserve protein stability) had no effect on ciliary localization. This suggests the critical function of the L2 motif (^428^VTVSAE^433^) in FGFR2 ciliary entry. When we substituted the FGFR2 L2 motif with corresponding sequences found in cilium-non-localizing FGFR3 (FGFR2::R3L2) and FGFR4 (FGFR2::R4L2), the FGFR2 ciliary localization was lost (Fig. 7 E; and Fig. S6, B and C). In a reciprocal experiment, the substitution of the sequences in FGFR3 (FGFR3::R2L2) and FGFR4 (FGFR4::R2L2) for the FGFR2 L2 motif completely restored their ciliary localization capacity (P < 0.0001, Welch’s *t* test; Fig. 7 F; and Fig. S6, B and C). When we placed the L2 motif into another position within the juxtamembrane region (FGFR2-ΔL2; R450_L451insL2) (Fig. S6, D and E), we did not observe any rescue in ciliary localization of the FGFR2-ΔL2 variant (Fig. S6 F). This suggests that topology of the region surrounding the L2 site likely contributes to ciliary trafficking. Taken together, we identified the FGFR2 L2 motif (^428^VTVSAE^433^) responsible for the ciliary localization of FGFRs.

The L2 motif lies just next to the region responsible for the binding of the FGFR adaptor protein FRS2 (^423^PLRRQ^427^ in FGFR2) (Fig. 7 C) (Hadari et al., 2001; Ridyard and Robbins, 2003; Ray et al., 2020). Therefore, we asked if deletion or substitution in the L2 motif interferes with FRS2 activation, and if so, does that have any function in the ciliary localization of FGFR2? For that, we overexpressed the FGFR2 variants with a modified L2 motif in 293T cells and analyzed phosphorylation of FGFR2, FRS2, and ERK. All tested FGFR2 variants showed inhibited levels of both basal and FGF-induced pFGFR, compared with wild-type FGFR2, by 74–87% (with P < 0.0001, Welch’s *t* test; Fig. 7 G). Furthermore, the FGFR2 variants showed inhibited levels of basal pFRS2 by 91–98% (with P = 0.0129–0.0472, Welch’s *t* test), and removal of L2 (FGFR2-ΔL2) produced a mild reduction in FGF-induced FRS2 phosphorylation compared with wild-type FGFR2 (by ∼27%; P = 0.04, Welch’s *t* test). To address this further, we produced a double-point FGFR2 mutant that disables interaction with FRS2 (FGFR2-L424A;R426A) (Ray et al., 2020), and tested its ciliary localization capacity in transfected IMCD3 cells. We found FGFR2-L424A;R426A to have only a mild reduction in ciliary localization capacity (by 30% on average with P = 0.0067, Welch’s *t* test; Fig. 7 H), which was not due to lesser expression (Fig. S6 G). Since the removal of the L2 motif abolished FGFR2 ciliary localization (by 78% on average with P < 0.0001, Welch’s *t* test; FGFR2-ΔL2 in Fig. 7 D), we can exclude the FRS2 binding as a critical mediator of FGFR2 ciliary localization.

To narrow down the FGFR2 amino acids responsible for ciliary entry, we aligned the region containing the L2 motif among vertebrates ranging from *Homo sapiens* to *Danio rerio* and found the whole region to be well conserved (Fig. 7 I). Correspondingly, the zebrafish Fgfr2 and also human FGFR2 and FGFR3 variants containing the zebrafish L2 motif (FGFR2::fL2 and FGFR3::fL2; Fig. S6, H and I) localized well to primary cilia of IMCD3 cells (Fig. 7 J). The evolutionary sequence alignment of FGFR2 further showed that T^429^V^430^, the two amino acids present within the FGFR1/2 L2 motif and absent in FGFR3 and FGFR4, are conserved back to zebrafish. This suggested that T^429^V^430^ are responsible for the FGFR2 ciliary entry. Indeed, deletion of T^429^V^430^ (FGFR2-ΔTV and FGFR2::fL2-ΔTV; Fig. S6, H and I) completely abolished ciliary localization of FGFR2 (Fig. 7 J).

### FGFR2 requires the ciliary localization T^429^V^430^ motif to signal

**Figure 8.**
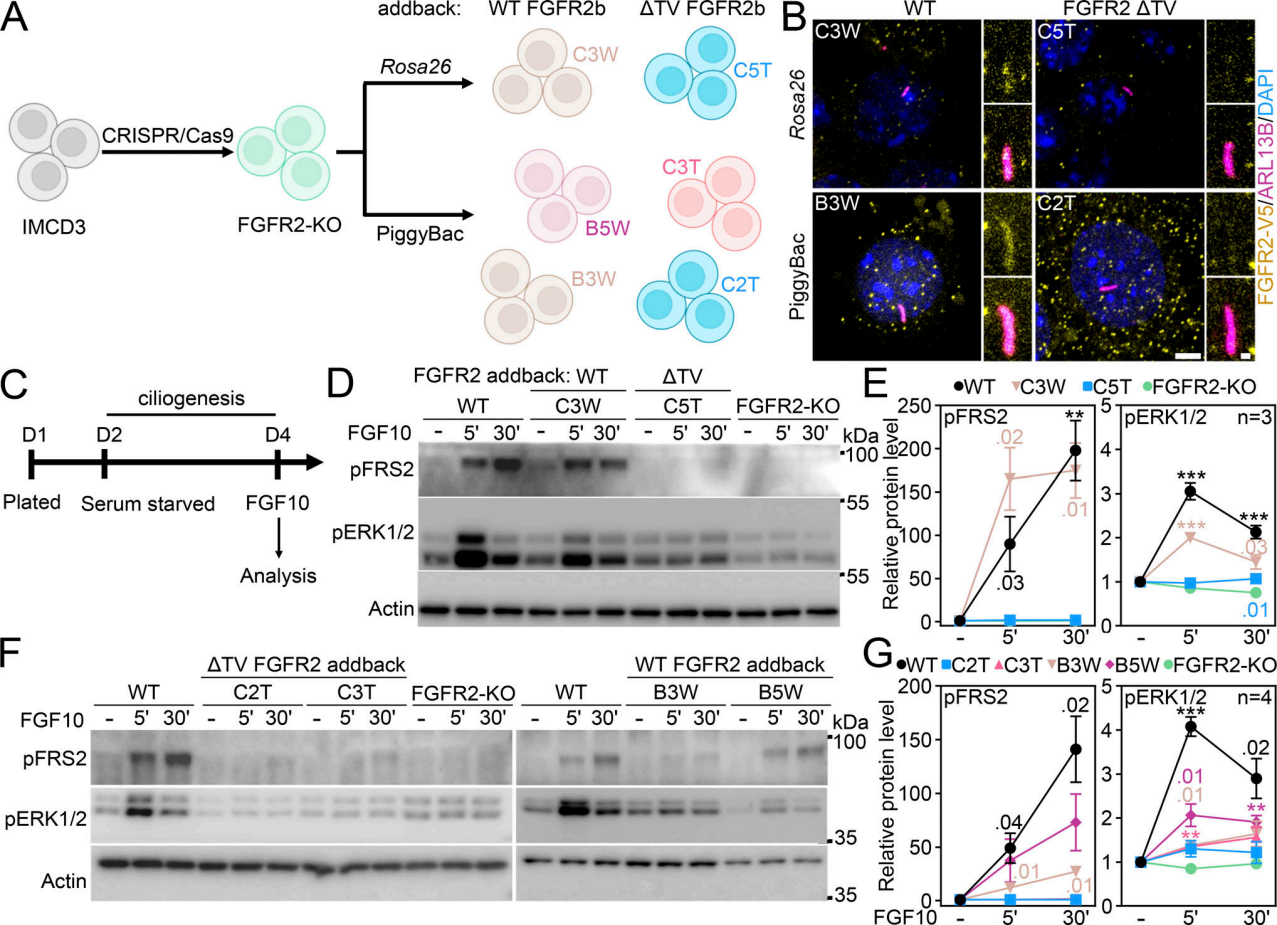
**FGFR2 requires the ciliary localization T** ^**429**^ **V** ^**430**^ **motif to signal. (A)** Scheme of the experimental strategies used to generate IMCD3 expressing human wild-type (WT) FGFR2b, or bearing the ΔTV mutation through an FGFR2 knock-out (KO) intermediate. The individual clones used in the subsequent experiments are shown. **(B)** Immunocytochemistry for ARL13B and V5 (FGFR2) in the newly generated cell lines using both PiggyBac and *Rosa26* targeting. The primary ciliary localization pattern confirms that the ΔTV FGFR2 is not able to enter the ciliary compartment. Scale bar, 2 μm for whole cell and 0.5 μm for cilium details. **(C)** Scheme of experiments assessing the signaling capabilities of the newly generated cell lines. **(D and E)** IMCD3 WT, FGFR2-KO or with FGFR2b addback in the *Rosa26* locus was subjected to blot analysis. IMCD3 cells with the wild-type FGFR2 addback cells (C3W) respond to FGF10 stimulation comparably to the parental cell line as seen in the phosphorylation (p) of FRS2 and ERK1/2. The non-ciliary ΔTV variant–expressing cells (C5T) failed to respond to the ligand, similar to the FGFR2-KO cells. The significance is displayed toward the non-treated cells. **(F and G)** IMCD3 FGFR2 addbacks generated by PiggyBac vector display a similar pattern in response to FGF10 stimulation. Two ΔTV FGFR2 addback lines (C2T, C3T) show no FRS2 phosphorylation and minimal downstream activation of ERK1/2. Conversely, the cells expressing the wild-type FGFR2 variant (B3W, B5W) display a signaling response pattern similar to the parental IMCD3. The significance is displayed toward the non-treated cells. Statistical significances were calculated using Welch’s *t* test (P < 0.05; **P < 0.01, ***P < 0.001); n.s., not significant. Line plots—mean ± SEM. The *n* value indicates the number of independent experiments. Source data are available for this figure: SourceData F8.

Next, we produced cells expressing FGFR2-ΔTV. For that, we first targeted the *Fgfr2* locus in IMCD3 cells in order to generate gene knockouts (*Fgfr2*-KO) (Fig. 8 A). The *Fgfr2*-KO cells were then transfected with either the *Rosa26* targeting vector or PiggyBac transposase to achieve stable transfection of either wild-type or ΔTV FGFR2. Importantly, the FGFR2-ΔTV addback cells failed to localize FGFR2 to the primary cilium, while the wild-type FGFR2 addbacks showed ciliary localization (Fig. 8 B). Next, we tested their FGF10 signaling response. For that, the serum-starved IMCD3 cells were treated with FGF10 for 5′ or 30′ (Fig. 8 C), and the signaling response was tested using pFRS2 and pERK1/2 readouts. As expected, the FGFR2-ΔTV addback cells failed to respond to FGF10, while the wild-type FGFR2 addback cells signaled similar to parental IMCD3 cells (Fig. 8, D–G).

## Discussion

**Figure 9.**
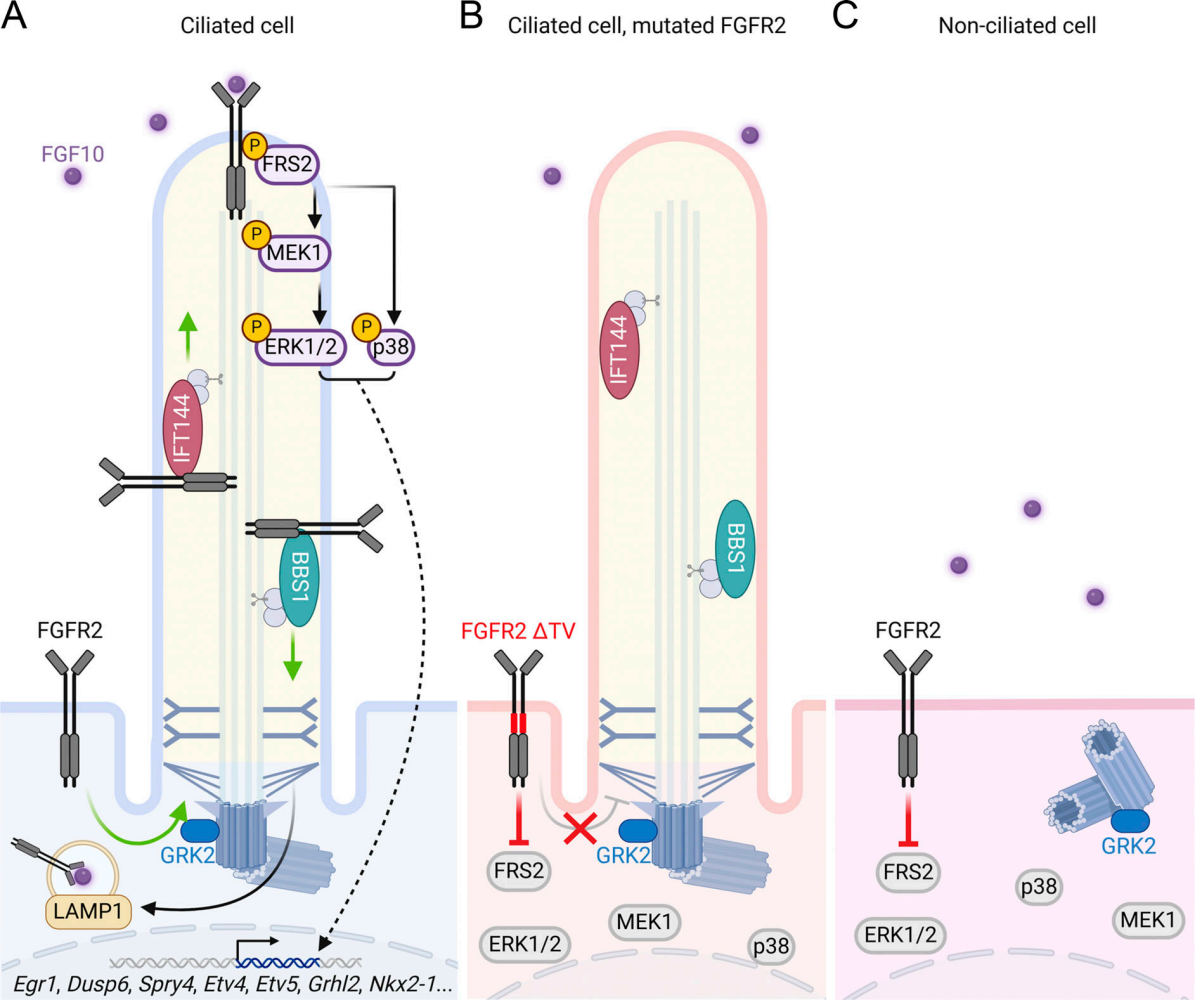
**Proposed mechanism of ciliary FGFR2 trafficking and signaling. (A)** In ciliated cells, FGFR2 accumulates in the primary cilium, which is supported by IFT144 and GRK2; BBS1 assists the ciliary exit. Upon FGF10 stimulation, the activated FGFR2 initiates the downstream signaling by phosphorylation (p) of FRS2, MEK1, ERK1/2, and p38 within the cilium, which results in transcription of the target genes. The activated FGFR2 exits the primary cilium and is targeted to the LAMP1 vesicles. **(B)** When the ciliary localization sequence is disrupted, FGFR2 is not allowed to enter the primary cilium and cannot signal. **(C)** When the primary cilium is lost in cells, the FGFR2 cannot signal.

In our paper, we discovered a novel cilium-resident receptor, FGFR2, identified the protein regulators of this process and the ciliary localization motif within FGFR2, and demonstrated the dependence of FGFR2 signaling on the primary cilia (Fig. 9). Interestingly, we found FGFR2 and FGFR1, but not FGFR3 and FGFR4, in primary cilia. This bias within the FGFR family appears to stem from the amino acid sequence of the juxtamembrane region that had preserved the ciliary localization T^429^V^430^ motif only within FGFR1 and FGFR2.

When stimulated with FGF10, FGFR2 activates the MAP kinase pathway directly within the primary cilia (Fig. 4). Some of the established ciliary RTKs show a similar pattern. For example, PDGFRα activation leads to phosphorylation of AKT and MEK1/2 within the cilium and at the basal body (Schneider et al., 2005, 2010). Similarly, IGF-1R activation results in phosphorylation of the receptor substrate IRS-1 and activation of AKT at the cilium (Zhu et al., 2009). For FGFR2, we showed the temporal dynamics of FGFR2 upon binding of FGF10. The activated FGFR2 leaves the cilium and recycles back through the perinuclear LAMP1-positive vesicular compartment (Fig. 4). Interestingly, we found LAMP1 also in the IMCD3 cilia. The CilioGenics database (Pir et al., 2024, https://ciliogenics.com) identified LAMP1 as a potential ciliary candidate, and in cultured human fibroblast-like synoviocytes, LAMP1 was found in the ciliary pocket (Rattner et al., 2010), which acts as a platform for cilium-associated vesicular trafficking (Benmerah, 2013; Ghossoub et al., 2016). The primary cilia of epithelial cells, including IMCD3, do not possess ciliary pockets (Molla-Herman et al., 2010); yet several members of the endocytic pathways have been identified within IMCD3 cilia, including CDC42 (Mick et al., 2015; Kohli et al., 2017), ARF6 (Ishikawa et al., 2012; Kohli et al., 2017), RhoA (Shinde et al., 2023; Kohli et al., 2017), and components of the ESCRT complexes (Mick et al., 2015; Shinde et al., 2023; May et al., 2021; Jung et al., 2020). Our data thus expand the epithelial ciliome by LAMP1.

We found that the disease-associated FGFR2 variants carrying an activating mutation have limited amounts in primary cilia (Fig. 5). The ciliary localization may regulate RTK activity, as exampled by PDGFRα, which, when not allowed in the cilia, responds to ligand stimulation with prolonged, stronger signaling due to impaired receptor internalization (Schmid et al., 2018). The mislocalization and ectopic activity of FGFRs have been shown to produce centrosome and cilium abnormalities, manifesting as changes in cell differentiation and cancerogenesis (Nita et al., 2021; Nielsen et al., 2015; Yin et al., 2021; Li et al., 2019). It is therefore tempting to speculate that the lost ability to localize to the primary cilium may contribute to the pathogenesis of the FGFR disorders. For example, individuals with bent bone dysplasia, and Crouzon and Apert syndromes display a wide range of morphological abnormalities in the skeletal tissue, including craniofacial dysmorphism, bent and hypoplastic bones, altered mineralization of the skull, craniosynostosis, syndactyly, or brachydactyly (Stichelbout et al., 2016; Reardon et al., 1994; Wilkie et al., 1995; Merrill et al., 2012). Similar features have been observed in ciliopathies involving the skeleton, including the Sensenbrenner, Jeune, Joubert, or the oral–facial–digital syndromes (Lin et al., 2013; Walczak-Sztulpa et al., 2010; de Vries et al., 2010; Dar et al., 2001; Saari et al., 2015; Goswami et al., 2016; Maria et al., 1999; Gorlin et al., 1961), or the short-rib thoracic dysplasia (Duran et al., 2017). To what extent does poor ciliary localization contribute to the pathogenesis of the FGFR2 disorders, and whether that would be improved by therapies targeting the FGFR2 activity remain to be resolved.

## Materials and methods

### Cell culture and treatments

293T (RRID:CVCL_0063), MCF-7 (RRID:CVCL_0031), 4MBr-5 (RRID:CVCL_0031), NIH3T3 (RRID:CVCL_0594), 3T3-L1 (RRID:CVCL_0123), and IMCD3 (RRID:CVCL_0429) cells were propagated in high-glucose DMEM (293T and NIH3T3) or DMEM:F12 (1:1; IMCD3) (Gibco) that was supplemented with 10% FBS (Gibco) and penicillin/streptomycin antibiotics (Sigma-Aldrich); the basal media for 3T3-L1 were DMEM buffered with NaHCO_3_ (PAN-Biotech). All cell lines were obtained from the ATCC and tested negative for *Mycoplasma* contamination. For induction of ciliogenesis, cells were grown without serum for 24–48 h (IMCD3 and 3T3-L1), or with 0.1% FBS overnight (NIH3T3), or treated with cytochalasin D (SCBT) in complete media for 24 h. Additional treatments included FGF1 and FGF10 (R&D Systems), ciliobrevin A, RLY-4008, paroxetine, and CMPD101 (Tocris). FGF10-DyLight 550 was produced using the DyLight 550 NHS Ester (Thermo Fisher Scientific) following the manufacturer’s procedures.

### Vectors and transfection

The pcDNA3.1 vectors carrying C-terminally V5-tagged FGFR1 (RRID:Addgene_201106), FGFR2 (RRID:Addgene_201107), FGFR3 (RRID:Addgene_201108), FGFR4 (RRID:Addgene_201109), and PGDFRα (RRID:Addgene_201987) were generated by cloning full-length human cDNA into a pcDNA3.1/V5-His TOPO TA vector (Invitrogen) (Gudernova et al., 2017). The FGFR vectors generated for this study are shown in Data S1 and were produced by PCR mutagenesis or site-directed mutagenesis (Agilent) (Table S1), verified by sequencing and their in-cell expression by immunoblotting. Zebrafish fgfr2 coding sequence (ref. ENSDART00000150061.3) tagged with V5 was synthesized by GeneArt (Thermo Fisher Scientific) and cloned into a pcDNA3.1 vector opened with NheI and PmeI. IMCD3 cells were transfected using Lipofectamine 2000 (Invitrogen); 293T cells were transfected using FuGENE 6 (Promega).

To produce IMCD3 cells stably transfected with dox-inducible shRNA, we used lentivirus transduction as before (Kunova Bosakova et al., 2019; Bosakova et al., 2020). Briefly, a lentiviral vector containing dox-inducible U6 promoter and TetRep-P2A-Puro-P2A-mCherry was modified to express shRNA by introducing the oligonucleotides for Ift172 or the scrambled control (Table S1). Lentiviral particles were generated using pMD2.G (RRID:Addgene_12259) and psPAX2 (RRID:Addgene_12260) (gift from Didier Trono, EPFL, Lausanne, Switzerland). After transduction, the mCherry-positive cells were sorted using BD FACSAria II (RRID:SCR_018934; BD Biosciences) and propagated in the presence of tetracycline-free FBS (Biosera) and 1 μg/ml puromycin (Gibco). The shRNA expression was induced by 1 μg/ml dox (Invitrogen) for a total of 3 days before the analysis.

For siRNA-mediated knockdown, the cells were transfected with 10 nM Ift144 (s102790), Rab23 (s72602), Arl6 (s80290), Bbs1 (s78731), or 20 nM Ift20 (s79931, Ambion) siRNA using Lipofectamine 2000. The next morning, the cells were given fresh complete media, and the 48-h (IMCD3) or 24-h (NIH3T3 and 3T3-L1) serum starvation was initiated the day after.

### CRISPR/Cas9 editing

A pair of CRISPR/Cas9 nickases (pX335-U6-Chimeric_BB-CBh-hSpCas9n(D10A); RRID:Addgene_42335; gift from Feng Zhang, Broad Institute of MIT and Harvard, Cambridge, MA, USA [Cong et al., 2013]) was used to generate FGFR2 knockout IMCD3 cells, and to open the *Rosa26* locus later on for production of the addback cells. Briefly, IMCD3 cells were electroporated with CRISPR/Cas9 plasmids targeting the *Fgfr2* locus by Neon electroporator (Thermo Fisher Scientific; 1,350 V; 20 ms; 2 pulses) with 100-μl tip according to the manual. Cells were then seeded at very low density and selected in 400 ng/ml G418 (InvivoGen), and the individual clonal colonies were manually picked, expanded, and tested for FGFR2 presence by western blot. Successful targeting was verified by Sanger sequencing of the *Fgfr2* locus. Cells negative for endogenous FGFR2 were then used for addback of human FGFR2-V5, either wild-type or the ΔTV variant. Two approaches were applied for the FGFR2-V5 addback. First, stable integration of the FGFR2-expressing plasmid was mediated by PiggyBac transposase (Hera BioLabs). FGFR2 variants were cloned into the TR01F plasmid (a gift form Valeri Mossiene, University of Missouri, Columbia, MO, USA [Mossine et al., 2013]), in which copGFP sequence was removed and NF-κB–responsive element with luciferase gene was replaced by the CMV promoter and the FGFR2-V5–encoding sequence. Both the PiggyBac plasmid and the modified TR01F plasmid were electroporated as described above, and cells with stable integration of FGFR2-V5 were selected in 2 µg/ml puromycin (Thermo Fisher Scientific) and validated by Sanger sequencing. The expression of FGFR2-V5 was tested by western blot, and clones with FGFR2-V5 expression similar to endogenous FGFR2 levels in the parental IMCD3 cells were picked and used in the subsequent analyses. For the second approach, a pair of CRISPR/Cas9 nickases targeting the *Rosa26* locus was electroporated into FGFR2 knockout IMCD3 cells together with the pDonor MCS Rosa26 plasmid (a gift from Charles Gersbach, Duke University, Durham, NC, USA; RRID:Addgene_37200 [Perez-Pinera et al., 2012]) containing insulated human FGFR2-V5 variants driven from the CMV promoter and the puromycin resistance cassette. The *Rosa26* clones were selected and validated similar to PiggyBac clones.

### qRT-PCR and western blot

For qRT-PCR, the total RNA was isolated with RNA Blue (TopBio), and the cDNA was produced using Transcriptor First Strand cDNA Synthesis Kit (Roche) or High-Capacity cDNA Reverse Transcription Kit (Thermo Fisher Scientific). The PCR was done using LightCycler 480 SYBR Green I Master Mix (Roche) or qPCR 2x SYBR Master Mix (TopBio) and analyzed using LightCycler 480 II (RRID:SCR_018626; Roche). The primers used are in Table S2.

For western blot, the cell lysates were harvested into the sample buffer (125 mM Tris–HCl, pH 6.8, 20% glycerol, 4% SDS, 5% β-mercaptoethanol, 0.02% bromophenol blue). The samples were resolved by SDS-PAGE and transferred on a PVDF membrane (Millipore or Amersham), probed, and visualized by chemiluminescence (Thermo Fisher Scientific). The antibodies used in the study are in Table S3. Western blots were imaged using the Fusion Solo device (Vilber), and the optic densitometry of the bands was measured using the GelAnalyzer plugin in Fiji (RRID:SCR_002285).

To determine changes in the electrophoretic migration of proteins, digitally acquired overlays of protein ladder and luminescent signal were measured in Fiji. The molecular mass of protein isoforms was calculated from a polynomial regression equation fitted to the migration of the protein ladder. Data from all independent experiments were averaged and the final values displayed in the figures.

### Immunocytochemistry and confocal imaging

The cells were fixed with 4% PFA/PBS (Sigma-Aldrich), permeabilized with 0.1% Triton X-100/PBS, blocked in 10% normal goat serum (Abcam) or 10% horse serum (Thermo Fisher Scientific), and incubated with primary antibodies overnight at 4°C; the incubation with secondary antibodies was at RT for 1 h. The antibodies used in the study are in Table S3. The coverslips were mounted using Mowiol 40-88 (Sigma-Aldrich), and Z-stacks were acquired using a Carl Zeiss LSM 700 (RRID:SCR_017377) laser scanning microscope with 63×/1.4 Oil DIC objective at room temperature, using ZEN Black 2012 software. Cilium lengths were determined in 3D using the View5D plugin in Fiji. RTK fluorescence was measured in Fiji as mean fluorescence intensity in the primary cilium area. For that, Z-stack images were acquired by focusing on the cells, identified with DAPI, that presented a signal for the primary cilium marked with ARL13B immunocytochemistry. The Z-stack limits were chosen according to the presence of the ARL13B signal, with the limits above and below the last detected signal for each cell’s cilium. The thickness of the Z-stack sections was ∼0.34 µm. To measure the RTK fluorescence in the primary cilium, wide-field Z-stack images capturing multiple cells were acquired in multiple distinct regions of the samples to eliminate bias. Subsequent analysis involved manual segmentation of the cilium using the ARL13B signal in the strongest Z-stack section, marking the region of interest to finally transpose it for measurement in the RTK channel. The same region of interest would subsequently be manually placed next to the primary cilium to measure the cellular background, which would later be subtracted from the measured ciliary intensity. The measurement output from Fiji would comprise area, mean, min, max, integrated density (IntDen = area (µm^2^) × mean), and total fluorescence (RawIntDen). For the transfected cells, the Z-stack images were acquired by focusing on the cells identified with DAPI, and showing signal for the RTK transfection. Cells with altered morphology (typically expressing very high transgene levels) were excluded from our confocal analyses, which hence involved only cells with low/moderate levels of transgene expression. For visualization purposes, the representative microscopy images used in figures had their brightness and contrast increased at the same rate throughout the individual figure panels.

### Animal handling, histology, and immunohistochemistry

Animal handling was performed according to the experimental protocol approved by Expert Committee for ensuring the ethical handling and welfare of animals at the Faculty of Medicine of Masaryk University according to the Czech and European regulations (permit number MSMT-6340/2021-3). C57BL/6N mice (RRID:MGI:2159965) were kept under specific-pathogen-free conditions on a 12-h light/dark cycle with free access to food and water. The embryos (*n* = 4) at stage E15.5-16.0 were obtained from pregnant normal healthy females ranging between 6 and 12 wk old, which were euthanized by a brief carbon dioxide inhalation followed by cervical dislocation. All obtained embryos were randomly assigned for this study or for different purposes. No sex distinction for the embryos was done in this study. The embryos were washed twice in PBS and fixed with 4% PFA/PBS (Sigma-Aldrich) at 4°C for 48 h. The embryos were embedded in paraffin, and the 8-µm-thick sections were used for histology and immunohistochemistry. The paraffin sections were deparaffinized with xylene, rehydrated using decreasing ethanol concentrations (100%, 96%, and 70%), and subjected to standard hematoxylin–eosin (Sigma-Aldrich) staining protocol. For immunohistochemistry, the hydrated sections were subjected to antigen retrieval with citrate buffer, pH 3.5, at 85°C for 60 min, permeabilized with 0.1% Triton X-100/PBS, blocked in 10% horse serum (Biosera), and incubated with primary antibodies at 4°C overnight; the incubation with secondary antibodies was at RT for 1 h. The antibodies used in the study are in Table S3. The labeled sections were mounted using DAPI-containing VectaShield (Vector Laboratories) and analyzed by confocal microscopy using Carl Zeiss LSM 700. Since the analysis did not observe a treatment effect, blinded analysis was not considered.

### Statistical analysis

All experiments were performed at least in triplicate unless stated otherwise; a formal power calculation was not performed. The *n* values express the number of independent biological experiments, performed as separate transfections and/or treatments performed at different days. In cell culture experiments, to quantify the cilium lengths and RTK ciliary fluorescence, between 22 and 50 primary cilia were analyzed per biological experiment. Data are presented as the mean ± SEM. for bar plots; scatter dot plots are graphed with the median line, and box–whisker plots display the 90/10 percentile at the whiskers. Two-tailed Welch’s *t* test was used to calculate the P values (P < 0.05 = P value specified in the figures instead of *; **P < 0.01, ***P < 0.001) using GraphPad Prism (RRID:SCR_002798). Data distribution was assumed to be normal, but this was not formally tested.

### Online supplemental material

Fig. S1 provides expanded view on FGFR2 immunohistochemistry of mouse embryonic tissues. Fig. S2 provides expanded view on cilium-dependent FGFR2 signaling. Fig. S3 provides expanded view on the expression of FGFRs and FGFR2 migration. Fig. S4 provides expanded view on activating FGFR2 variants. Fig. S5 provides expanded view on molecular regulators of ciliary FGFR trafficking. Fig. S6 provides expanded view on the FGFR constructs tested for ciliary localization. Table S1 provides a list of oligonucleotides used in this study. Table S2 provides a list of qRT-PCR primers used in this study. Table S3 provides a list of antibodies used in this study. Data S1 provides sequences of constructs generated for this study.

## Supplementary Material

Table S1 provides a list of oligonucleotides used in this study.

Table S2 provides a list of qRT-PCR primers used in this study.

Table S3 provides a list of antibodies used in this study.

Data S1 shows sequences of constructs generated for this study.

SourceData F2 is the source file for Fig. 2.

SourceData F3 is the source file for Fig. 3.

SourceData F5 is the source file for Fig. 5.

SourceData F6 is the source file for Fig. 6.

SourceData F7 is the source file for Fig. 7.

SourceData F8 is the source file for Fig. 8.

SourceData FS2 is the source file for Fig. S2.

SourceData FS3 is the source file for Fig. S3.

SourceData FS6 is the source file for Fig. S6.

## Data Availability

All data needed to evaluate the conclusions in the paper are present in the paper and/or the Supplementary Materials. The materials developed for this study are available upon request and following the Material Transfer Agreement.
