# Supplementary material for: FGFR2 residence in primary cilia is necessary for epithelial cell signaling
Source: J Cell Biol. 2025 Apr 22;224(7):e202311030. doi: 10.1083/jcb.202311030 (PMC12010920; doi:10.1083/jcb.202311030)
Supplement: Table S1 — provides a list of oligonucleotides used in this study. [file jcb_202311030_tables1.docx]

Table S1. List of oligonucleotides used in this study.

| *Name* | | *Application* | | | *Sequence 5'-3'* |
| --- | --- | --- | --- | --- | --- |
| Scrambled_fwd | | shRNA | | CCGGGCCGTACTTACTCTCAGTTTACTCGAGTAAACTGAGAGTAAGTACGGCTTTTTG | |
| Scrambled_rev | | shRNA | | AATTCAAAAAGCCGTACTTACTCTCAGTTTACTCGAGTAAACTGAGAGTAAGTACGGC | |
| IFT172_fwd | | shRNA | | CCGGGCTGCTGATCTCTCATTACTACTCGAGTAGTAATGAGAGATCAGCAGCTTTTTG | |
| IFT172_rev | | shRNA | | AATTCAAAAAGCTGCTGATCTCTCATTACTACTCGAGTAGTAATGAGAGATCAGCAGC | |
| FGFR2_fwd | | PCR cloning | | CTAGTTAAGCTTGGTACCGAGC | |
| FGFR2_rev | | PCR cloning | | GGGCCCTCTAGACTCGAG | |
| ΔTK/C-t_rev | | PCR cloning | | TCTAGACTCGAGCGGCCGCCACTGTGCTGGATATCTGCAGAATTGTCTTGACCCTTGGCTTCATACTCGGAGACCCCTG | |
| ΔTK_fwd | | PCR cloning | | CAGGGGTCTCCGAGTATGAAAATGAGGAATACTTGGACCTCAG | |
| ΔTK_rev | | PCR cloning | | AGGTCCAAGTATTCCTCATTTTCATACTCGGAGACCCCTG | |
| ΔC-t_rev | | PCR cloning | | TCTAGACTCGAGCGGCCGCCACTGTGCTGGATATCTGCAGAATTGTCTTGACCCTTGGCGGTTGTGAGAGTGAGAATTCG | |
| ΔJ/TK_fwd | | PCR cloning | | TGTGCCGAATGAAGAACACGAATGAGGAATACTTGGACCTCAG | |
| ΔJ/TK_rev | | PCR cloning | | AGGTCCAAGTATTCCTCATTCGTGTTCTTCATTCGGCACAG | |
| FGFR2-ΔL2_fwd | | PCR cloning | | GTATCCCCCTGCGGAGACAGTCCAGCTCCTCCATGAACTCC | |
| FGFR2-ΔL2_rev | | PCR cloning | | GGAGTTCATGGAGGAGCTGGACTGTCTCCGCAGGGGGATACG | |
| FGFR2-ΔL1_fwd | | PCR cloning | | CCGGCTGTGCACAAGCT | |
| FGFR2-ΔL1_rev | | PCR cloning | | GTTCTTCATTCGGCACAGG | |
| FGFR2::R3L1_fwd | | PCR cloning | | CAAGAAAGGCCTGGGCTCCCCGGCTGTGCACAAG | |
| FGFR2::R3L1_rev | | PCR cloning | | CCAGGCCTTTCTTGGGGGGGTTCTTCATTCGGCACA | |
| FGFR3::R2L2_fwd | | PCR cloning | | GTGACAGTTTCGGCTGAGTCCAACGCGTCC | |
| FGFR3::R2L2_rev | | PCR cloning | | CTCAGCCGAAACTGTCACCTGTCGCTTGAG | |
| FGFR4::R2L2_fwd | | PCR cloning | | GTAACAGTTTCGGCTGAGTCAGGCTCTTCC | |
| FGFR4::R2L2_rev | | PCR cloning | | AGCCGAAACTGTTACCTGTCGGGCCAGA | |
| FGFR2::R3L2_fwd | | PCR cloning | | AGGTATCACTGGAGTCCAGCTCCTCCATGA | |
| FGFR2::R3L2_rev | | PCR cloning | | GGACTCCAGTGATACCTGTCTCCGCAGGGG | |
| FGFR2::R4L2_fwd | | PCR cloning | | TTCTCACTGGAGTCCAGCTCCTCCATGA | |
| FGFR2::R4L2_rev | | PCR cloning | | TGGACTCCAGTGAGAACTGTCTCCGCAGGG | |
| FGFR2-A432L_fwd | | Site mutagenesis | | GAGACAGGTAACAGTTTCGCTAGAGTCCAGCTCCTCCATGA | |
| FGFR2-A432L_rev | | Site mutagenesis | | TCATGGAGGAGCTGGACTCTAGCGAAACTGTTACCTGTCTC | |
| FGFR2-ΔTV_fwd | | Site mutagenesis | | CCCTGCGGAGACAGGTATCGGCTGAG | |
| FGFR2-ΔTV_rev | | Site mutagenesis | | CTCAGCCGATACCTGTCTCCGCAGGG | |
| FGFR2::fL2_fwd | | Site mutagenesis | | GAGACAGGTAACAGTTTCGAGTGATTCCAGCTCCTCCATGAACT | |
| FGFR2::fL2_rev | | Site mutagenesis | | AGTTCATGGAGGAGCTGGAATCACTCGAAACTGTTACCTGTCTC | |
| FGFR2::fL2-ΔTV_fwd | | Site mutagenesis | | CCCCCTGCGGAGACAGGTATCGAGTGATT | |
| FGFR2::fL2-ΔTV_rev | | Site mutagenesis | | AATCACTCGATACCTGTCTCCGCAGGGGG | |
| FGFR3::f2L2_fwd | | Site mutagenesis | | GACAGGTGACAGTTTCGAGTGATTCCAACGCGTCCATGAG | |
| FGFR3::f2L2_rev | | Site mutagenesis | | CTCATGGACGCGTTGGAATCACTCGAAACTGTCACCTGTC | |
| L424A;R426A_fwd | | Site mutagenesis | | CTGACCAAACGTATCCCCGCGCGGGCACAGGTAACAGTTTCGGC | |
| L424A;R426A_rev | | Site mutagenesis | | GCCGAAACTGTTACCTGTGCCCGCGCGGGGATACGTTTGGTCAG | |
| FGFR2 M391R_fwd | | Site mutagenesis | | GTCTTCTTAATCGCCTGTAGGGTGGTAACAGTCATCC | |
| FGFR2 M391R_rev | | Site mutagenesis | | GGATGACTGTTACCACCCTACAGGCGATTAAGAAGAC | |
| R450_L451insVTVSAE_fwd | | Site mutagenesis | | GAGGATAACAACACGCGTAACAGTTTCGGCTGAGCTCTCTTCAACGGCAG | |
| R450_L451insVTVSAE_rev | | Site mutagenesis | | CTGCCGTTGAAGAGAGCTCAGCCGAAACTGTTACGCGTGTTGTTATCCTC | |
| mFgfr2#ex1#Crispr1.1 | | CRISPR gRNA | | caccgCACTTCTTCTGAGACCATGG * | |
| mFgfr2#ex1#Crispr1.2 | | CRISPR gRNA | | aaacCCATGGTCTCAGAAGAAGTGc * | |
| mFgfr2#ex1#Crispr2.1 | | CRISPR gRNA | | caccgGAGATATGGAAGAGGACCA * | |
| mFgfr2#ex1#Crispr2.2 | | CRISPR gRNA | | aaacTGGTCCTCTTCCATATCTCc * | |
| mRosa26 Crispr 1.1 | | CRISPR gRNA | | caccgGAGGGGGAAGGGATTCTCCC * | |
| mRosa26 Crispr 1.2 | | CRISPR gRNA | | aaacGGGAGAATCCCTTCCCCCTCc * | |
| mRosa26 Crispr 2.1 | | CRISPR gRNA | | caccgACTCCAGTCTTTCTAGAAGA * | |
| mRosa26 Crispr 2.2 | | CRISPR gRNA | | aaacTCTTCTAGAAAGACTGGAGTc * | |
| hU6-F | | Sequencing | | gagggcctatttcccatgatt | |
| FmFgfr2#Crispr-seq | | Sequencing | | aggaacagcagtaacaacagca | |
| RmFgfr2#Crispr-seq | | Sequencing | | CCAAAACGACTTGGTACCCTTA | |
| F_R26_outer_left | | Sequencing | | GCCGACGTCTCGTCGCTGATTG | |
| R_R26_outer_right | | Sequencing | | GTAAGCAGTAATCAATACCATGTGGCTC | |
| F_R26_inner_right | | Sequencing | | atatgggccggaagtcctaatatgg | |
| R_R26_inner_left | | Sequencing | | TTCCCCGTCAAGCTCTAAATCGGGG | |
| Fm-CMV-Fgfr2-Puro#InFusion | | PCR cloning | | CTGATCAGCGGGTTTCAGTCAATCTTTCACAAATTTTGT | |
| Rm-CMV-Fgfr2-Puro#InFusion | | PCR cloning | | GGCCACTAGTGCTAGTATGCCAAGTACGCCCCCT | |
|  |  | |  | | |

* the lower-case letters are the overhangs needed for insertion of the gRNA coding sequence into the CRISPR plasmid
