# Supplementary material for: FGFR2 residence in primary cilia is necessary for epithelial cell signaling
Source: J Cell Biol. 2025 Apr 22;224(7):e202311030. doi: 10.1083/jcb.202311030 (PMC12010920; doi:10.1083/jcb.202311030)
Supplement: Table S2 — provides a list of qRT-PCR primers used in this study. [file jcb_202311030_tables2.docx]

Table S2. List of qRT-PCR primers used in this study.

| *Primer name* | *Sequence* |
| --- | --- |
| Bbs1_fwd | AGAACCTGGCAGATGAGGAC |
| Bbs1_rev | GGGTCCAGCACTAGGAGTTC |
| Dusp6_fwd | ATAGATACGCTCAGACCCGTG |
| Dusp6_rev | ATCAGCAGAAGCCGTTCGTT |
| Etv4_fwd | CGGAGGATGAAAGGCGGATAC |
| Etv4_rev | TCTTGGAAGTGACTGAGGTCC |
| Etv5_fwd | TCAGTCTGATAACTTGGTGCTTC |
| Etv5_rev | GGCTTCCTATCGTAGGCACAA |
| Fgfr1_rev | TTGGTGCCGCTCTTCATCTT |
| Fgfr1b_fwd | CGGGAATTAATAGCTCGGATGC |
| Fgfr1c_fwd | GGAGTTAATACCACCGACAAG |
| Fgfr2-all-isoforms_rev | AGATGACTGTCACCACCATGCA |
| Fgfr2b_fwd | CACTCGGGGATAAATAGCTCC |
| Fgfr2c_fwd | CGGTGTTAACACCACGGAC |
| Fgfr3-all-isoforms_fwd | CGCCCTACGTCACTGTACTCAA |
| Fgfr3b_rev | GTCCCGCTCCGACACATT |
| Fgfr3c_rev | GTGACATTGTGCAAGGACAGAAC |
| Fgfr4_fwd | GTACCCTCGGACCGCGGCACATAC |
| Fgfr4_rev | GCCGAAGCTGCTGCCGTTGATG |
| Gapdh_fwd | AACTTTGGCATTGTGGAAGG |
| Gapdh_rev | ATCCACAGTCTTCTGGGTGG |
| Grhl2_fwd | AAGCCCAGTGCAACAACTCC |
| Grhl2_rev | TGTCCGGTCCTCTGTAGGTTT |
| Hoxb13_fwd | GGGGTCGGAATCTAGTCTCCC |
| Hoxb13_rev | CCTCCAAAGTAGCCATAAGGCA |
| Id2_fwd | CGACCCGATGAGTCTGCTCTA |
| Id2_rev | GACGATAGTGGGATGCGAGTC |
| Nkx2-1_fwd | AACTGCGGGGATCTGAGCTG |
| Nkx2-1_rev | CATGATTCGGCGTCGGCTGG |
| Nkx3-1_fwd | ATGCTTAGGGTAGCGGAGC |
| Nkx3-1_rev | TGCGGATTGCCTGAGTGTC |
| Sox9_fwd | CGGAACAGACTCACATCTCTCC |
| Sox9_rev | GCTTGCACGTCGGTTTTGG |
| Sp8_fwd | GCTACCTGTAATAAGATCGGCAG |
| Sp8_rev | GAGGAGCGTTTCCAAGGGTG |
| Spry2_fwd | CTGAAGCAGAGTTTGGAAAG |
| Spry2_rev | CTTAGAACACATCTGAACTCC |
| Spry4_fwd | CCTCAAAGACCCCTAGAAG |
| Spry4_rev | GTCTTCATCTGGTCAATGG |
| Ubb_fwd | ATGTGAAGGCCAAGATCCAG |
| Ubb_rev | TAATAGCCACCCCTCAGACG |
