# Supplementary material for: FGFR2 residence in primary cilia is necessary for epithelial cell signaling
Source: J Cell Biol. 2025 Apr 22;224(7):e202311030. doi: 10.1083/jcb.202311030 (PMC12010920; doi:10.1083/jcb.202311030)
Supplement: Table S3 — provides a list of antibodies used in this study. [file jcb_202311030_tables3.docx]

Table S3. List of antibodies used in this study.

| *Target* | *Application* | *Cat. No.* | *Vendor* |
| --- | --- | --- | --- |
| β-Actin (mouse mAb) | WB | 3700 | Cell Signaling Technology |
| ARL13B (rabbit pAb) | ICC, IHC | 17711-1-AP | Proteintech |
| ARL13B (mouse mAb) | ICC, IHC | 66739-1-Ig | Proteintech |
| ARL6 (rabbit pAb) | WB | 12676-1-AP | Proteintech |
| EGR1 (rabbit pAb) | WB | 4154 | Cell Signaling Technology |
| ERK (rabbit pAb) | WB | 9102 | Cell Signaling Technology |
| FGFR1 (rabbit mAb) | WB | 9740 | Cell Signaling Technology |
| FGFR1 (rabbit pAb) | ICC | sc-7945 | Santa Cruz Biotechnology |
| FGFR2 (rabbit pAb) | WB | sc-122 | Santa Cruz Biotechnology |
| FGFR2 (rabbit mAb) | WB | 23328 | Cell Signaling Technology |
| FGFR2 (goat pAb) | IHC | sc-122G | Santa Cruz Biotechnology |
| FGFR2 (mouse mAb) | ICC | sc-6930 | Santa Cruz Biotechnology |
| IFT144 (rabbit pAb) | WB | 13647-1-AP | Proteintech |
| IFT172 (mouse mAb) | WB | sc-398393 | Santa Cruz Biotechnology |
| IFT20 (rabbit pAb) | WB | 13615-1-AP | Proteintech |
| LAMP1 (rat mAb) | ICC | sc-19992 | Santa Cruz Biotechnology |
| p-AKT^T308^ ­ (rabbit pAb) | WB | 9275 | Cell Signaling Technology |
| p-c-Raf^S289/296/301^­ (rabbit pAb) | WB | 9431 | Cell Signaling Technology |
| p-ERK1/2^T202/Y204^ (rabbit mAb) | WB | 4376 | Cell Signaling Technology |
| p-ERK1/2^T202/Y204^ (rabbit pAb) | WB | 9101 | Cell Signaling Technology |
| p-FGFR^Y653/654^ (rabbit pAb) | WB | 3471 | Cell Signaling Technology |
| p-FGFR^Y653/654^ (rabbit pAb) | WB | AF3285 | R&D Systems |
| p-FRS2^Y196^ (rabbit pAb) | WB | 3864 | Cell Signaling Technology |
| p-FRS2^Y196^ (rabbit pAb) | ICC | 3861 | Cell Signaling Technology |
| p-GAB1^Y307^ (rabbit pAb) | WB | 3234 | Cell Signaling Technology |
| p-MEK1/2^S217/221^ (rabbit pAb) | WB, ICC | 9121 | Cell Signaling Technology |
| p-p130 CAS^Y249^ (rabbit pAb) | WB | 4014 | Cell Signaling Technology |
| p-p38 MAPK^T180/Y182^ (rabbit mAb) | WB | 9215 | Cell Signaling Technology |
| p-p38 MAPK^T180/Y182^ (rabbit mAb) | ICC | 4631 | Cell Signaling Technology |
| p-p70S6K^T389^ (rabbit pAb) | WB, ICC | 9205 | Cell Signaling Technology |
| p-SHC^Y239/240^ (rabbit pAb) | WB | 2434 | Cell Signaling Technology |
| p-STAT3^S727^ (rabbit pAb) | WB | 9134 | Cell Signaling Technology |
| RAB23 (mouse mAb) | WB | sc-517357 | Santa Cruz Biotechnology |
| V5 (mouse mAb) | WB, ICC | 46-0705 | Invitrogen |
| Sheep anti-mouse HRP | WB | A6782 | Sigma-Aldrich |
| Goat anti-rabbit HRP | WB | A0545 | Sigma-Aldrich |
| Rabbit anti-goat HRP | WB | A4174 | Sigma-Aldrich |
| Donkey anti-Mouse Alexa Fluor® 488 | ICC, IHC | A21202 | Thermo Fisher Scientific |
| Donkey anti-Rabbit Alexa Fluor® 488 | ICC, IHC | A21206 | Thermo Fisher Scientific |
| Donkey anti-Goat Alexa Fluor® 488 | ICC, IHC | A11055 | Thermo Fisher Scientific |
| Donkey anti-Mouse Alexa Fluor® 568 | ICC, IHC | A10037 | Thermo Fisher Scientific |
| Donkey anti-Rabbit Alexa Fluor® 568 | ICC, IHC | A10042 | Thermo Fisher Scientific |
| Donkey anti-Mouse Alexa Fluor® 594 | ICC, IHC | A21203 | Thermo Fisher Scientific |
| Donkey anti-Rabbit Alexa Fluor® 594 | ICC, IHC | A21207 | Thermo Fisher Scientific |
| Donkey anti-Rabbit Alexa Fluor® 647 | ICC, IHC | A31573 | Thermo Fisher Scientific |
