# Supplementary material for: FGFR2 residence in primary cilia is necessary for epithelial cell signaling
Source: J Cell Biol. 2025 Apr 22;224(7):e202311030. doi: 10.1083/jcb.202311030 (PMC12010920; doi:10.1083/jcb.202311030)
Supplement: Data S1 — shows sequences of constructs generated for this study. [file jcb_202311030_datas1.docx]

**Data S1.** Sequences of constructs generated for this study.

pcDNA3.1-ΔC-t:

TCTCTGGCTAACTAGAGAACCCACTGCTTACTGGCTTATCGAAATTAATACGACTCACTATAGGGAGACCCAAGCTGGCTAGTTAAGCTTGGTACCGAGCTCGGATCCAGTACCCTTCACCATGGTCAGCTGGGGTCGTTTCATCTGCCTGGTCGTGGTCACCATGGCAACCTTGTCCCTGGCCCGGCCCTCCTTCAGTTTAGTTGAGGATACCACATTAGAGCCAGAAGAGCCACCAACCAAATACCAAATCTCTCAACCAGAAGTGTACGTGGCTGCGCCAGGGGAGTCGCTAGAGGTGCGCTGCCTGTTGAAAGATGCCGCCGTGATCAGTTGGACTAAGGATGGGGTGCACTTGGGGCCCAACAATAGGACAGTGCTTATTGGGGAGTACTTGCAGATAAAGGGCGCCACGCCTAGAGACTCCGGCCTCTATGCTTGTACTGCCAGTAGGACTGTAGACAGTGAAACTTGGTACTTCATGGTGAATGTCACAGATGCCATCTCATCCGGAGATGATGAGGATGACACCGATGGTGCGGAAGATTTTGTCAGTGAGAACAGTAACAACAAGAGAGCACCATACTGGACCAACACAGAAAAGATGGAAAAGCGGCTCCATGCTGTGCCTGCGGCCAACACTGTCAAGTTTCGCTGCCCAGCCGGGGGGAACCCAATGCCAACCATGCGGTGGCTGAAAAACGGGAAGGAGTTTAAGCAGGAGCATCGCATTGGAGGCTACAAGGTACGAAACCAGCACTGGAGCCTCATTATGGAAAGTGTGGTCCCATCTGACAAGGGAAATTATACCTGTGTAGTGGAGAATGAATACGGGTCCATCAATCACACGTACCACCTGGATGTTGTGGAGCGATCGCCTCACCGGCCCATCCTCCAAGCCGGACTGCCGGCAAATGCCTCCACAGTGGTCGGAGGAGACGTAGAGTTTGTCTGCAAGGTTTACAGTGATGCCCAGCCCCACATCCAGTGGATCAAGCACGTGGAAAAGAACGGCAGTAAATACGGGCCCGACGGGCTGCCCTACCTCAAGGTTCTCAAGGCCGCCGGTGTTAACACCACGGACAAAGAGATTGAGGTTCTCTATATTCGGAATGTAACTTTTGAGGACGCTGGGGAATATACGTGCTTGGCGGGTAATTCTATTGGGATATCCTTTCACTCTGCATGGTTGACAGTTCTGCCAGCGCCTGGAAGAGAAAAGGAGATTACAGCTTCCCCAGACTACCTGGAGATAGCCATTTACTGCATAGGGGTCTTCTTAATCGCCTGTATGGTGGTAACAGTCATCCTGTGCCGAATGAAGAACACGACCAAGAAGCCAGACTTCAGCAGCCAGCCGGCTGTGCACAAGCTGACCAAACGTATCCCCCTGCGGAGACAGGTAACAGTTTCGGCTGAGTCCAGCTCCTCCATGAACTCCAACACCCCGCTGGTGAGGATAACAACACGCCTCTCTTCAACGGCAGACACCCCCATGCTGGCAGGGGTCTCCGAGTATGAACTTCCAGAGGACCCAAAATGGGAGTTTCCAAGAGATAAGCTGACACTGGGCAAGCCCCTGGGAGAAGGTTGCTTTGGGCAAGTGGTCATGGCGGAAGCAGTGGGAATTGACAAAGACAAGCCCAAGGAGGCGGTCACCGTGGCCGTGAAGATGTTGAAAGATGATGCCACAGAGAAAGACCTTTCTGATCTGGTGTCAGAGATGGAGATGATGAAGATGATTGGGAAACACAAGAATATCATAAATCTTCTTGGAGCCTGCACACAGGATGGGCCTCTCTATGTCATAGTTGAGTATGCCTCTAAAGGCAACCTCCGAGAATACCTCCGAGCCCGGAGGCCACCCGGGATGGAGTACTCCTATGACATTAACCGTGTTCCTGAGGAGCAGATGACCTTCAAGGACTTGGTGTCATGCACCTACCAGCTGGCCAGAGGCATGGAGTACTTGGCTTCCCAAAAATGTATTCATCGAGATTTAGCAGCCAGAAATGTTTTGGTAACAGAAAACAATGTGATGAAAATAGCAGACTTTGGACTCGCCAGAGATATCAACAATATAGACTATTACAAAAAGACCACCAATGGGCGGCTTCCAGTCAAGTGGATGGCTCCAGAAGCCCTGTTTGATAGAGTATACACTCATCAGAGTGATGTCTGGTCCTTCGGGGTGTTAATGTGGGAGATCTTCACTTTAGGGGGCTCGCCCTACCCAGGGATTCCCGTGGAGGAACTTTTTAAGCTGCTGAAGGAAGGACACAGAATGGATAAGCCAGCCAACTGCACCAACGAACTGTACATGATGATGAGGGACTGTTGGCATGCAGTGCCCTCCCAGAGACCAACGTTCAAGCAGTTGGTAGAAGACTTGGATCGAATTCTCACTCTCACAACCGCCAAGGGTCAAGACAATTCTGCAGATATCCAGCACAGTGGCGGCCGCTCGAGTCTAGAGGGCCCGCGGTTCGAAGGTAAGCCTATCCCTAACCCTCTCCTCGGTCTCGATTCTACGCGTACCGGTCATCATCACCATCACCATTGA

Receptor sequence

V5 tag

6x His

pcDNA3.1-ΔTK:

TCTCTGGCTAACTAGAGAACCCACTGCTTACTGGCTTATCGAAATTAATACGACTCACTATAGGGAGACCCAAGCTGGCTAGTTAAGCTTGGTACCGAGCTCGGATCCAGTACCCTTCACCATGGTCAGCTGGGGTCGTTTCATCTGCCTGGTCGTGGTCACCATGGCAACCTTGTCCCTGGCCCGGCCCTCCTTCAGTTTAGTTGAGGATACCACATTAGAGCCAGAAGAGCCACCAACCAAATACCAAATCTCTCAACCAGAAGTGTACGTGGCTGCGCCAGGGGAGTCGCTAGAGGTGCGCTGCCTGTTGAAAGATGCCGCCGTGATCAGTTGGACTAAGGATGGGGTGCACTTGGGGCCCAACAATAGGACAGTGCTTATTGGGGAGTACTTGCAGATAAAGGGCGCCACGCCTAGAGACTCCGGCCTCTATGCTTGTACTGCCAGTAGGACTGTAGACAGTGAAACTTGGTACTTCATGGTGAATGTCACAGATGCCATCTCATCCGGAGATGATGAGGATGACACCGATGGTGCGGAAGATTTTGTCAGTGAGAACAGTAACAACAAGAGAGCACCATACTGGACCAACACAGAAAAGATGGAAAAGCGGCTCCATGCTGTGCCTGCGGCCAACACTGTCAAGTTTCGCTGCCCAGCCGGGGGGAACCCAATGCCAACCATGCGGTGGCTGAAAAACGGGAAGGAGTTTAAGCAGGAGCATCGCATTGGAGGCTACAAGGTACGAAACCAGCACTGGAGCCTCATTATGGAAAGTGTGGTCCCATCTGACAAGGGAAATTATACCTGTGTAGTGGAGAATGAATACGGGTCCATCAATCACACGTACCACCTGGATGTTGTGGAGCGATCGCCTCACCGGCCCATCCTCCAAGCCGGACTGCCGGCAAATGCCTCCACAGTGGTCGGAGGAGACGTAGAGTTTGTCTGCAAGGTTTACAGTGATGCCCAGCCCCACATCCAGTGGATCAAGCACGTGGAAAAGAACGGCAGTAAATACGGGCCCGACGGGCTGCCCTACCTCAAGGTTCTCAAGGCCGCCGGTGTTAACACCACGGACAAAGAGATTGAGGTTCTCTATATTCGGAATGTAACTTTTGAGGACGCTGGGGAATATACGTGCTTGGCGGGTAATTCTATTGGGATATCCTTTCACTCTGCATGGTTGACAGTTCTGCCAGCGCCTGGAAGAGAAAAGGAGATTACAGCTTCCCCAGACTACCTGGAGATAGCCATTTACTGCATAGGGGTCTTCTTAATCGCCTGTATGGTGGTAACAGTCATCCTGTGCCGAATGAAGAACACGACCAAGAAGCCAGACTTCAGCAGCCAGCCGGCTGTGCACAAGCTGACCAAACGTATCCCCCTGCGGAGACAGGTAACAGTTTCGGCTGAGTCCAGCTCCTCCATGAACTCCAACACCCCGCTGGTGAGGATAACAACACGCCTCTCTTCAACGGCAGACACCCCCATGCTGGCAGGGGTCTCCGAGTATGAAAATGAGGAATACTTGGACCTCAGCCAACCTCTCGAACAGTATTCACCTAGTTACCCTGACACAAGAAGTTCTTGTTCTTCAGGAGATGATTCTGTTTTTTCTCCAGACCCCATGCCTTACGAACCATGCCTTCCTCAGTATCCACACATAAACGGCAGTGTTAAAACAGCCAAGGGTCAAGACAATTCTGCAGATATCCAGCACAGTGGCGGCCGCTCGAGTCTAGAGGGCCCGCGGTTCGAAGGTAAGCCTATCCCTAACCCTCTCCTCGGTCTCGATTCTACGCGTACCGGTCATCATCACCATCACCATTGA

Receptor sequence

V5 tag

6x His

pcDNA3.1-ΔTK/C-t:

TCTCTGGCTAACTAGAGAACCCACTGCTTACTGGCTTATCGAAATTAATACGACTCACTATAGGGAGACCCAAGCTGGCTAGTTAAGCTTGGTACCGAGCTCGGATCCAGTACCCTTCACCATGGTCAGCTGGGGTCGTTTCATCTGCCTGGTCGTGGTCACCATGGCAACCTTGTCCCTGGCCCGGCCCTCCTTCAGTTTAGTTGAGGATACCACATTAGAGCCAGAAGAGCCACCAACCAAATACCAAATCTCTCAACCAGAAGTGTACGTGGCTGCGCCAGGGGAGTCGCTAGAGGTGCGCTGCCTGTTGAAAGATGCCGCCGTGATCAGTTGGACTAAGGATGGGGTGCACTTGGGGCCCAACAATAGGACAGTGCTTATTGGGGAGTACTTGCAGATAAAGGGCGCCACGCCTAGAGACTCCGGCCTCTATGCTTGTACTGCCAGTAGGACTGTAGACAGTGAAACTTGGTACTTCATGGTGAATGTCACAGATGCCATCTCATCCGGAGATGATGAGGATGACACCGATGGTGCGGAAGATTTTGTCAGTGAGAACAGTAACAACAAGAGAGCACCATACTGGACCAACACAGAAAAGATGGAAAAGCGGCTCCATGCTGTGCCTGCGGCCAACACTGTCAAGTTTCGCTGCCCAGCCGGGGGGAACCCAATGCCAACCATGCGGTGGCTGAAAAACGGGAAGGAGTTTAAGCAGGAGCATCGCATTGGAGGCTACAAGGTACGAAACCAGCACTGGAGCCTCATTATGGAAAGTGTGGTCCCATCTGACAAGGGAAATTATACCTGTGTAGTGGAGAATGAATACGGGTCCATCAATCACACGTACCACCTGGATGTTGTGGAGCGATCGCCTCACCGGCCCATCCTCCAAGCCGGACTGCCGGCAAATGCCTCCACAGTGGTCGGAGGAGACGTAGAGTTTGTCTGCAAGGTTTACAGTGATGCCCAGCCCCACATCCAGTGGATCAAGCACGTGGAAAAGAACGGCAGTAAATACGGGCCCGACGGGCTGCCCTACCTCAAGGTTCTCAAGGCCGCCGGTGTTAACACCACGGACAAAGAGATTGAGGTTCTCTATATTCGGAATGTAACTTTTGAGGACGCTGGGGAATATACGTGCTTGGCGGGTAATTCTATTGGGATATCCTTTCACTCTGCATGGTTGACAGTTCTGCCAGCGCCTGGAAGAGAAAAGGAGATTACAGCTTCCCCAGACTACCTGGAGATAGCCATTTACTGCATAGGGGTCTTCTTAATCGCCTGTATGGTGGTAACAGTCATCCTGTGCCGAATGAAGAACACGACCAAGAAGCCAGACTTCAGCAGCCAGCCGGCTGTGCACAAGCTGACCAAACGTATCCCCCTGCGGAGACAGGTAACAGTTTCGGCTGAGTCCAGCTCCTCCATGAACTCCAACACCCCGCTGGTGAGGATAACAACACGCCTCTCTTCAACGGCAGACACCCCCATGCTGGCAGGGGTCTCCGAGTATGAAGCCAAGGGTCAAGACAATTCTGCAGATATCCAGCACAGTGGCGGCCGCTCGAGTCTAGAGGGCCCGCGGTTCGAAGGTAAGCCTATCCCTAACCCTCTCCTCGGTCTCGATTCTACGCGTACCGGTCATCATCACCATCACCATTGA

Receptor sequence

V5 tag

6x His

pcDNA3.1-ΔJ/TK:

TCTCTGGCTAACTAGAGAACCCACTGCTTACTGGCTTATCGAAATTAATACGACTCACTATAGGGAGACCCAAGCTGGCTAGTTAAGCTTGGTACCGAGCTCGGATCCAGTACCCTTCACCATGGTCAGCTGGGGTCGTTTCATCTGCCTGGTCGTGGTCACCATGGCAACCTTGTCCCTGGCCCGGCCCTCCTTCAGTTTAGTTGAGGATACCACATTAGAGCCAGAAGAGCCACCAACCAAATACCAAATCTCTCAACCAGAAGTGTACGTGGCTGCGCCAGGGGAGTCGCTAGAGGTGCGCTGCCTGTTGAAAGATGCCGCCGTGATCAGTTGGACTAAGGATGGGGTGCACTTGGGGCCCAACAATAGGACAGTGCTTATTGGGGAGTACTTGCAGATAAAGGGCGCCACGCCTAGAGACTCCGGCCTCTATGCTTGTACTGCCAGTAGGACTGTAGACAGTGAAACTTGGTACTTCATGGTGAATGTCACAGATGCCATCTCATCCGGAGATGATGAGGATGACACCGATGGTGCGGAAGATTTTGTCAGTGAGAACAGTAACAACAAGAGAGCACCATACTGGACCAACACAGAAAAGATGGAAAAGCGGCTCCATGCTGTGCCTGCGGCCAACACTGTCAAGTTTCGCTGCCCAGCCGGGGGGAACCCAATGCCAACCATGCGGTGGCTGAAAAACGGGAAGGAGTTTAAGCAGGAGCATCGCATTGGAGGCTACAAGGTACGAAACCAGCACTGGAGCCTCATTATGGAAAGTGTGGTCCCATCTGACAAGGGAAATTATACCTGTGTAGTGGAGAATGAATACGGGTCCATCAATCACACGTACCACCTGGATGTTGTGGAGCGATCGCCTCACCGGCCCATCCTCCAAGCCGGACTGCCGGCAAATGCCTCCACAGTGGTCGGAGGAGACGTAGAGTTTGTCTGCAAGGTTTACAGTGATGCCCAGCCCCACATCCAGTGGATCAAGCACGTGGAAAAGAACGGCAGTAAATACGGGCCCGACGGGCTGCCCTACCTCAAGGTTCTCAAGGCCGCCGGTGTTAACACCACGGACAAAGAGATTGAGGTTCTCTATATTCGGAATGTAACTTTTGAGGACGCTGGGGAATATACGTGCTTGGCGGGTAATTCTATTGGGATATCCTTTCACTCTGCATGGTTGACAGTTCTGCCAGCGCCTGGAAGAGAAAAGGAGATTACAGCTTCCCCAGACTACCTGGAGATAGCCATTTACTGCATAGGGGTCTTCTTAATCGCCTGTATGGTGGTAACAGTCATCCTGTGCCGAATGAAGAACACGAATGAGGAATACTTGGACCTCAGCCAACCTCTCGAACAGTATTCACCTAGTTACCCTGACACAAGAAGTTCTTGTTCTTCAGGAGATGATTCTGTTTTTTCTCCAGACCCCATGCCTTACGAACCATGCCTTCCTCAGTATCCACACATAAACGGCAGTGTTAAAACAGCCAAGGGTCAAGACAATTCTGCAGATATCCAGCACAGTGGCGGCCGCTCGAGTCTAGAGGGCCCGCGGTTCGAAGGTAAGCCTATCCCTAACCCTCTCCTCGGTCTCGATTCTACGCGTACCGGTCATCATCACCATCACCATTGA

Receptor sequence

V5 tag

6x His

pcDNA3.1-FGFR2-ΔL1:

TCTCTGGCTAACTAGAGAACCCACTGCTTACTGGCTTATCGAAATTAATACGACTCACTATAGGGAGACCCAAGCTGGCTAGTTAAGCTTGGTACCGAGCTCGGATCCAGTACCCTTCACCATGGTCAGCTGGGGTCGTTTCATCTGCCTGGTCGTGGTCACCATGGCAACCTTGTCCCTGGCCCGGCCCTCCTTCAGTTTAGTTGAGGATACCACATTAGAGCCAGAAGAGCCACCAACCAAATACCAAATCTCTCAACCAGAAGTGTACGTGGCTGCGCCAGGGGAGTCGCTAGAGGTGCGCTGCCTGTTGAAAGATGCCGCCGTGATCAGTTGGACTAAGGATGGGGTGCACTTGGGGCCCAACAATAGGACAGTGCTTATTGGGGAGTACTTGCAGATAAAGGGCGCCACGCCTAGAGACTCCGGCCTCTATGCTTGTACTGCCAGTAGGACTGTAGACAGTGAAACTTGGTACTTCATGGTGAATGTCACAGATGCCATCTCATCCGGAGATGATGAGGATGACACCGATGGTGCGGAAGATTTTGTCAGTGAGAACAGTAACAACAAGAGAGCACCATACTGGACCAACACAGAAAAGATGGAAAAGCGGCTCCATGCTGTGCCTGCGGCCAACACTGTCAAGTTTCGCTGCCCAGCCGGGGGGAACCCAATGCCAACCATGCGGTGGCTGAAAAACGGGAAGGAGTTTAAGCAGGAGCATCGCATTGGAGGCTACAAGGTACGAAACCAGCACTGGAGCCTCATTATGGAAAGTGTGGTCCCATCTGACAAGGGAAATTATACCTGTGTAGTGGAGAATGAATACGGGTCCATCAATCACACGTACCACCTGGATGTTGTGGAGCGATCGCCTCACCGGCCCATCCTCCAAGCCGGACTGCCGGCAAATGCCTCCACAGTGGTCGGAGGAGACGTAGAGTTTGTCTGCAAGGTTTACAGTGATGCCCAGCCCCACATCCAGTGGATCAAGCACGTGGAAAAGAACGGCAGTAAATACGGGCCCGACGGGCTGCCCTACCTCAAGGTTCTCAAGGCCGCCGGTGTTAACACCACGGACAAAGAGATTGAGGTTCTCTATATTCGGAATGTAACTTTTGAGGACGCTGGGGAATATACGTGCTTGGCGGGTAATTCTATTGGGATATCCTTTCACTCTGCATGGTTGACAGTTCTGCCAGCGCCTGGAAGAGAAAAGGAGATTACAGCTTCCCCAGACTACCTGGAGATAGCCATTTACTGCATAGGGGTCTTCTTAATCGCCTGTATGGTGGTAACAGTCATCCTGTGCCGAATGAAGAACCCGGCTGTGCACAAGCTGACCAAACGTATCCCCCTGCGGAGACAGGTAACAGTTTCGGCTGAGTCCAGCTCCTCCATGAACTCCAACACCCCGCTGGTGAGGATAACAACACGCCTCTCTTCAACGGCAGACACCCCCATGCTGGCAGGGGTCTCCGAGTATGAACTTCCAGAGGACCCAAAATGGGAGTTTCCAAGAGATAAGCTGACACTGGGCAAGCCCCTGGGAGAAGGTTGCTTTGGGCAAGTGGTCATGGCGGAAGCAGTGGGAATTGACAAAGACAAGCCCAAGGAGGCGGTCACCGTGGCCGTGAAGATGTTGAAAGATGATGCCACAGAGAAAGACCTTTCTGATCTGGTGTCAGAGATGGAGATGATGAAGATGATTGGGAAACACAAGAATATCATAAATCTTCTTGGAGCCTGCACACAGGATGGGCCTCTCTATGTCATAGTTGAGTATGCCTCTAAAGGCAACCTCCGAGAATACCTCCGAGCCCGGAGGCCACCCGGGATGGAGTACTCCTATGACATTAACCGTGTTCCTGAGGAGCAGATGACCTTCAAGGACTTGGTGTCATGCACCTACCAGCTGGCCAGAGGCATGGAGTACTTGGCTTCCCAAAAATGTATTCATCGAGATTTAGCAGCCAGAAATGTTTTGGTAACAGAAAACAATGTGATGAAAATAGCAGACTTTGGACTCGCCAGAGATATCAACAATATAGACTATTACAAAAAGACCACCAATGGGCGGCTTCCAGTCAAGTGGATGGCTCCAGAAGCCCTGTTTGATAGAGTATACACTCATCAGAGTGATGTCTGGTCCTTCGGGGTGTTAATGTGGGAGATCTTCACTTTAGGGGGCTCGCCCTACCCAGGGATTCCCGTGGAGGAACTTTTTAAGCTGCTGAAGGAAGGACACAGAATGGATAAGCCAGCCAACTGCACCAACGAACTGTACATGATGATGAGGGACTGTTGGCATGCAGTGCCCTCCCAGAGACCAACGTTCAAGCAGTTGGTAGAAGACTTGGATCGAATTCTCACTCTCACAACCAATGAGGAATACTTGGACCTCAGCCAACCTCTCGAACAGTATTCACCTAGTTACCCTGACACAAGAAGTTCTTGTTCTTCAGGAGATGATTCTGTTTTTTCTCCAGACCCCATGCCTTACGAACCATGCCTTCCTCAGTATCCACACATAAACGGCAGTGTTAAAACAGCCAAGGGTCAAGACAATTCTGCAGATATCCAGCACAGTGGCGGCCGCTCGAGTCTAGAGGGCCCGCGGTTCGAAGGTAAGCCTATCCCTAACCCTCTCCTCGGTCTCGATTCTACGCGTACCGGTCATCATCACCATCACCATTGA

Receptor sequence

V5 tag

6x His

pcDNA3.1-FGFR2-ΔL2:

TCTCTGGCTAACTAGAGAACCCACTGCTTACTGGCTTATCGAAATTAATACGACTCACTATAGGGAGACCCAAGCTGGCTAGTTAAGCTTGGTACCGAGCTCGGATCCAGTACCCTTCACCATGGTCAGCTGGGGTCGTTTCATCTGCCTGGTCGTGGTCACCATGGCAACCTTGTCCCTGGCCCGGCCCTCCTTCAGTTTAGTTGAGGATACCACATTAGAGCCAGAAGAGCCACCAACCAAATACCAAATCTCTCAACCAGAAGTGTACGTGGCTGCGCCAGGGGAGTCGCTAGAGGTGCGCTGCCTGTTGAAAGATGCCGCCGTGATCAGTTGGACTAAGGATGGGGTGCACTTGGGGCCCAACAATAGGACAGTGCTTATTGGGGAGTACTTGCAGATAAAGGGCGCCACGCCTAGAGACTCCGGCCTCTATGCTTGTACTGCCAGTAGGACTGTAGACAGTGAAACTTGGTACTTCATGGTGAATGTCACAGATGCCATCTCATCCGGAGATGATGAGGATGACACCGATGGTGCGGAAGATTTTGTCAGTGAGAACAGTAACAACAAGAGAGCACCATACTGGACCAACACAGAAAAGATGGAAAAGCGGCTCCATGCTGTGCCTGCGGCCAACACTGTCAAGTTTCGCTGCCCAGCCGGGGGGAACCCAATGCCAACCATGCGGTGGCTGAAAAACGGGAAGGAGTTTAAGCAGGAGCATCGCATTGGAGGCTACAAGGTACGAAACCAGCACTGGAGCCTCATTATGGAAAGTGTGGTCCCATCTGACAAGGGAAATTATACCTGTGTAGTGGAGAATGAATACGGGTCCATCAATCACACGTACCACCTGGATGTTGTGGAGCGATCGCCTCACCGGCCCATCCTCCAAGCCGGACTGCCGGCAAATGCCTCCACAGTGGTCGGAGGAGACGTAGAGTTTGTCTGCAAGGTTTACAGTGATGCCCAGCCCCACATCCAGTGGATCAAGCACGTGGAAAAGAACGGCAGTAAATACGGGCCCGACGGGCTGCCCTACCTCAAGGTTCTCAAGGCCGCCGGTGTTAACACCACGGACAAAGAGATTGAGGTTCTCTATATTCGGAATGTAACTTTTGAGGACGCTGGGGAATATACGTGCTTGGCGGGTAATTCTATTGGGATATCCTTTCACTCTGCATGGTTGACAGTTCTGCCAGCGCCTGGAAGAGAAAAGGAGATTACAGCTTCCCCAGACTACCTGGAGATAGCCATTTACTGCATAGGGGTCTTCTTAATCGCCTGTATGGTGGTAACAGTCATCCTGTGCCGAATGAAGAACACGACCAAGAAGCCAGACTTCAGCAGCCAGCCGGCTGTGCACAAGCTGACCAAACGTATCCCCCTGCGGAGACAGTCCAGCTCCTCCATGAACTCCAACACCCCGCTGGTGAGGATAACAACACGCCTCTCTTCAACGGCAGACACCCCCATGCTGGCAGGGGTCTCCGAGTATGAACTTCCAGAGGACCCAAAATGGGAGTTTCCAAGAGATAAGCTGACACTGGGCAAGCCCCTGGGAGAAGGTTGCTTTGGGCAAGTGGTCATGGCGGAAGCAGTGGGAATTGACAAAGACAAGCCCAAGGAGGCGGTCACCGTGGCCGTGAAGATGTTGAAAGATGATGCCACAGAGAAAGACCTTTCTGATCTGGTGTCAGAGATGGAGATGATGAAGATGATTGGGAAACACAAGAATATCATAAATCTTCTTGGAGCCTGCACACAGGATGGGCCTCTCTATGTCATAGTTGAGTATGCCTCTAAAGGCAACCTCCGAGAATACCTCCGAGCCCGGAGGCCACCCGGGATGGAGTACTCCTATGACATTAACCGTGTTCCTGAGGAGCAGATGACCTTCAAGGACTTGGTGTCATGCACCTACCAGCTGGCCAGAGGCATGGAGTACTTGGCTTCCCAAAAATGTATTCATCGAGATTTAGCAGCCAGAAATGTTTTGGTAACAGAAAACAATGTGATGAAAATAGCAGACTTTGGACTCGCCAGAGATATCAACAATATAGACTATTACAAAAAGACCACCAATGGGCGGCTTCCAGTCAAGTGGATGGCTCCAGAAGCCCTGTTTGATAGAGTATACACTCATCAGAGTGATGTCTGGTCCTTCGGGGTGTTAATGTGGGAGATCTTCACTTTAGGGGGCTCGCCCTACCCAGGGATTCCCGTGGAGGAACTTTTTAAGCTGCTGAAGGAAGGACACAGAATGGATAAGCCAGCCAACTGCACCAACGAACTGTACATGATGATGAGGGACTGTTGGCATGCAGTGCCCTCCCAGAGACCAACGTTCAAGCAGTTGGTAGAAGACTTGGATCGAATTCTCACTCTCACAACCAATGAGGAATACTTGGACCTCAGCCAACCTCTCGAACAGTATTCACCTAGTTACCCTGACACAAGAAGTTCTTGTTCTTCAGGAGATGATTCTGTTTTTTCTCCAGACCCCATGCCTTACGAACCATGCCTTCCTCAGTATCCACACATAAACGGCAGTGTTAAAACAGCCAAGGGTCAAGACAATTCTGCAGATATCCAGCACAGTGGCGGCCGCTCGAGTCTAGAGGGCCCGCGGTTCGAAGGTAAGCCTATCCCTAACCCTCTCCTCGGTCTCGATTCTACGCGTACCGGTCATCATCACCATCACCATTGA

Receptor sequence

V5 tag

6x His

pcDNA3.1-FGFR2-ΔL1L2:

TCTCTGGCTAACTAGAGAACCCACTGCTTACTGGCTTATCGAAATTAATACGACTCACTATAGGGAGACCCAAGCTGGCTAGTTAAGCTTGGTACCGAGCTCGGATCCAGTACCCTTCACCATGGTCAGCTGGGGTCGTTTCATCTGCCTGGTCGTGGTCACCATGGCAACCTTGTCCCTGGCCCGGCCCTCCTTCAGTTTAGTTGAGGATACCACATTAGAGCCAGAAGAGCCACCAACCAAATACCAAATCTCTCAACCAGAAGTGTACGTGGCTGCGCCAGGGGAGTCGCTAGAGGTGCGCTGCCTGTTGAAAGATGCCGCCGTGATCAGTTGGACTAAGGATGGGGTGCACTTGGGGCCCAACAATAGGACAGTGCTTATTGGGGAGTACTTGCAGATAAAGGGCGCCACGCCTAGAGACTCCGGCCTCTATGCTTGTACTGCCAGTAGGACTGTAGACAGTGAAACTTGGTACTTCATGGTGAATGTCACAGATGCCATCTCATCCGGAGATGATGAGGATGACACCGATGGTGCGGAAGATTTTGTCAGTGAGAACAGTAACAACAAGAGAGCACCATACTGGACCAACACAGAAAAGATGGAAAAGCGGCTCCATGCTGTGCCTGCGGCCAACACTGTCAAGTTTCGCTGCCCAGCCGGGGGGAACCCAATGCCAACCATGCGGTGGCTGAAAAACGGGAAGGAGTTTAAGCAGGAGCATCGCATTGGAGGCTACAAGGTACGAAACCAGCACTGGAGCCTCATTATGGAAAGTGTGGTCCCATCTGACAAGGGAAATTATACCTGTGTAGTGGAGAATGAATACGGGTCCATCAATCACACGTACCACCTGGATGTTGTGGAGCGATCGCCTCACCGGCCCATCCTCCAAGCCGGACTGCCGGCAAATGCCTCCACAGTGGTCGGAGGAGACGTAGAGTTTGTCTGCAAGGTTTACAGTGATGCCCAGCCCCACATCCAGTGGATCAAGCACGTGGAAAAGAACGGCAGTAAATACGGGCCCGACGGGCTGCCCTACCTCAAGGTTCTCAAGGCCGCCGGTGTTAACACCACGGACAAAGAGATTGAGGTTCTCTATATTCGGAATGTAACTTTTGAGGACGCTGGGGAATATACGTGCTTGGCGGGTAATTCTATTGGGATATCCTTTCACTCTGCATGGTTGACAGTTCTGCCAGCGCCTGGAAGAGAAAAGGAGATTACAGCTTCCCCAGACTACCTGGAGATAGCCATTTACTGCATAGGGGTCTTCTTAATCGCCTGTATGGTGGTAACAGTCATCCTGTGCCGAATGAAGAACCCGGCTGTGCACAAGCTGACCAAACGTATCCCCCTGCGGAGACAGTCCAGCTCCTCCATGAACTCCAACACCCCGCTGGTGAGGATAACAACACGCCTCTCTTCAACGGCAGACACCCCCATGCTGGCAGGGGTCTCCGAGTATGAACTTCCAGAGGACCCAAAATGGGAGTTTCCAAGAGATAAGCTGACACTGGGCAAGCCCCTGGGAGAAGGTTGCTTTGGGCAAGTGGTCATGGCGGAAGCAGTGGGAATTGACAAAGACAAGCCCAAGGAGGCGGTCACCGTGGCCGTGAAGATGTTGAAAGATGATGCCACAGAGAAAGACCTTTCTGATCTGGTGTCAGAGATGGAGATGATGAAGATGATTGGGAAACACAAGAATATCATAAATCTTCTTGGAGCCTGCACACAGGATGGGCCTCTCTATGTCATAGTTGAGTATGCCTCTAAAGGCAACCTCCGAGAATACCTCCGAGCCCGGAGGCCACCCGGGATGGAGTACTCCTATGACATTAACCGTGTTCCTGAGGAGCAGATGACCTTCAAGGACTTGGTGTCATGCACCTACCAGCTGGCCAGAGGCATGGAGTACTTGGCTTCCCAAAAATGTATTCATCGAGATTTAGCAGCCAGAAATGTTTTGGTAACAGAAAACAATGTGATGAAAATAGCAGACTTTGGACTCGCCAGAGATATCAACAATATAGACTATTACAAAAAGACCACCAATGGGCGGCTTCCAGTCAAGTGGATGGCTCCAGAAGCCCTGTTTGATAGAGTATACACTCATCAGAGTGATGTCTGGTCCTTCGGGGTGTTAATGTGGGAGATCTTCACTTTAGGGGGCTCGCCCTACCCAGGGATTCCCGTGGAGGAACTTTTTAAGCTGCTGAAGGAAGGACACAGAATGGATAAGCCAGCCAACTGCACCAACGAACTGTACATGATGATGAGGGACTGTTGGCATGCAGTGCCCTCCCAGAGACCAACGTTCAAGCAGTTGGTAGAAGACTTGGATCGAATTCTCACTCTCACAACCAATGAGGAATACTTGGACCTCAGCCAACCTCTCGAACAGTATTCACCTAGTTACCCTGACACAAGAAGTTCTTGTTCTTCAGGAGATGATTCTGTTTTTTCTCCAGACCCCATGCCTTACGAACCATGCCTTCCTCAGTATCCACACATAAACGGCAGTGTTAAAACAAAGGGTCAAGACAATTCTGCAGATATCCAGCACAGTGGCGGCCGCTCGAGTCTAGAGGGCCCGCGGTTCGAAGGTAAGCCTATCCCTAACCCTCTCCTCGGTCTCGATTCTACGCGTACCGGTCATCATCACCATCACCATTGA

Receptor sequence

V5 tag

6x His

pcDNA3.1-FGFR2::R3L1:

TCTCTGGCTAACTAGAGAACCCACTGCTTACTGGCTTATCGAAATTAATACGACTCACTATAGGGAGACCCAAGCTGGCTAGTTAAGCTTGGTACCGAGCTCGGATCCAGTACCCTTCACCATGGTCAGCTGGGGTCGTTTCATCTGCCTGGTCGTGGTCACCATGGCAACCTTGTCCCTGGCCCGGCCCTCCTTCAGTTTAGTTGAGGATACCACATTAGAGCCAGAAGAGCCACCAACCAAATACCAAATCTCTCAACCAGAAGTGTACGTGGCTGCGCCAGGGGAGTCGCTAGAGGTGCGCTGCCTGTTGAAAGATGCCGCCGTGATCAGTTGGACTAAGGATGGGGTGCACTTGGGGCCCAACAATAGGACAGTGCTTATTGGGGAGTACTTGCAGATAAAGGGCGCCACGCCTAGAGACTCCGGCCTCTATGCTTGTACTGCCAGTAGGACTGTAGACAGTGAAACTTGGTACTTCATGGTGAATGTCACAGATGCCATCTCATCCGGAGATGATGAGGATGACACCGATGGTGCGGAAGATTTTGTCAGTGAGAACAGTAACAACAAGAGAGCACCATACTGGACCAACACAGAAAAGATGGAAAAGCGGCTCCATGCTGTGCCTGCGGCCAACACTGTCAAGTTTCGCTGCCCAGCCGGGGGGAACCCAATGCCAACCATGCGGTGGCTGAAAAACGGGAAGGAGTTTAAGCAGGAGCATCGCATTGGAGGCTACAAGGTACGAAACCAGCACTGGAGCCTCATTATGGAAAGTGTGGTCCCATCTGACAAGGGAAATTATACCTGTGTAGTGGAGAATGAATACGGGTCCATCAATCACACGTACCACCTGGATGTTGTGGAGCGATCGCCTCACCGGCCCATCCTCCAAGCCGGACTGCCGGCAAATGCCTCCACAGTGGTCGGAGGAGACGTAGAGTTTGTCTGCAAGGTTTACAGTGATGCCCAGCCCCACATCCAGTGGATCAAGCACGTGGAAAAGAACGGCAGTAAATACGGGCCCGACGGGCTGCCCTACCTCAAGGTTCTCAAGGCCGCCGGTGTTAACACCACGGACAAAGAGATTGAGGTTCTCTATATTCGGAATGTAACTTTTGAGGACGCTGGGGAATATACGTGCTTGGCGGGTAATTCTATTGGGATATCCTTTCACTCTGCATGGTTGACAGTTCTGCCAGCGCCTGGAAGAGAAAAGGAGATTACAGCTTCCCCAGACTACCTGGAGATAGCCATTTACTGCATAGGGGTCTTCTTAATCGCCTGTATGGTGGTAACAGTCATCCTGTGCCGAATGAAGAACCCCCCCAAGAAAGGCCTGGGCTCCCCGGCTGTGCACAAGCTGACCAAACGTATCCCCCTGCGGAGACAGGTAACAGTTTCGGCTGAGTCCAGCTCCTCCATGAACTCCAACACCCCGCTGGTGAGGATAACAACACGCCTCTCTTCAACGGCAGACACCCCCATGCTGGCAGGGGTCTCCGAGTATGAACTTCCAGAGGACCCAAAATGGGAGTTTCCAAGAGATAAGCTGACACTGGGCAAGCCCCTGGGAGAAGGTTGCTTTGGGCAAGTGGTCATGGCGGAAGCAGTGGGAATTGACAAAGACAAGCCCAAGGAGGCGGTCACCGTGGCCGTGAAGATGTTGAAAGATGATGCCACAGAGAAAGACCTTTCTGATCTGGTGTCAGAGATGGAGATGATGAAGATGATTGGGAAACACAAGAATATCATAAATCTTCTTGGAGCCTGCACACAGGATGGGCCTCTCTATGTCATAGTTGAGTATGCCTCTAAAGGCAACCTCCGAGAATACCTCCGAGCCCGGAGGCCACCCGGGATGGAGTACTCCTATGACATTAACCGTGTTCCTGAGGAGCAGATGACCTTCAAGGACTTGGTGTCATGCACCTACCAGCTGGCCAGAGGCATGGAGTACTTGGCTTCCCAAAAATGTATTCATCGAGATTTAGCAGCCAGAAATGTTTTGGTAACAGAAAACAATGTGATGAAAATAGCAGACTTTGGACTCGCCAGAGATATCAACAATATAGACTATTACAAAAAGACCACCAATGGGCGGCTTCCAGTCAAGTGGATGGCTCCAGAAGCCCTGTTTGATAGAGTATACACTCATCAGAGTGATGTCTGGTCCTTCGGGGTGTTAATGTGGGAGATCTTCACTTTAGGGGGCTCGCCCTACCCAGGGATTCCCGTGGAGGAACTTTTTAAGCTGCTGAAGGAAGGACACAGAATGGATAAGCCAGCCAACTGCACCAACGAACTGTACATGATGATGAGGGACTGTTGGCATGCAGTGCCCTCCCAGAGACCAACGTTCAAGCAGTTGGTAGAAGACTTGGATCGAATTCTCACTCTCACAACCAATGAGGAATACTTGGACCTCAGCCAACCTCTCGAACAGTATTCACCTAGTTACCCTGACACAAGAAGTTCTTGTTCTTCAGGAGATGATTCTGTTTTTTCTCCAGACCCCATGCCTTACGAACCATGCCTTCCTCAGTATCCACACATAAACGGCAGTGTTAAAACAAAGGGTCAAGACAATTCTGCAGATATCCAGCACAGTGGCGGCCGCTCGAGTCTAGAGGGCCCGCGGTTCGAAGGTAAGCCTATCCCTAACCCTCTCCTCGGTCTCGATTCTACGCGTACCGGTCATCATCACCATCACCATTGA

Receptor sequence

V5 tag

6x His

pcDNA3.1-FGFR2::R3L2:

TCTCTGGCTAACTAGAGAACCCACTGCTTACTGGCTTATCGAAATTAATACGACTCACTATAGGGAGACCCAAGCTGGCTAGTTAAGCTTGGTACCGAGCTCGGATCCAGTACCCTTCACCATGGTCAGCTGGGGTCGTTTCATCTGCCTGGTCGTGGTCACCATGGCAACCTTGTCCCTGGCCCGGCCCTCCTTCAGTTTAGTTGAGGATACCACATTAGAGCCAGAAGAGCCACCAACCAAATACCAAATCTCTCAACCAGAAGTGTACGTGGCTGCGCCAGGGGAGTCGCTAGAGGTGCGCTGCCTGTTGAAAGATGCCGCCGTGATCAGTTGGACTAAGGATGGGGTGCACTTGGGGCCCAACAATAGGACAGTGCTTATTGGGGAGTACTTGCAGATAAAGGGCGCCACGCCTAGAGACTCCGGCCTCTATGCTTGTACTGCCAGTAGGACTGTAGACAGTGAAACTTGGTACTTCATGGTGAATGTCACAGATGCCATCTCATCCGGAGATGATGAGGATGACACCGATGGTGCGGAAGATTTTGTCAGTGAGAACAGTAACAACAAGAGAGCACCATACTGGACCAACACAGAAAAGATGGAAAAGCGGCTCCATGCTGTGCCTGCGGCCAACACTGTCAAGTTTCGCTGCCCAGCCGGGGGGAACCCAATGCCAACCATGCGGTGGCTGAAAAACGGGAAGGAGTTTAAGCAGGAGCATCGCATTGGAGGCTACAAGGTACGAAACCAGCACTGGAGCCTCATTATGGAAAGTGTGGTCCCATCTGACAAGGGAAATTATACCTGTGTAGTGGAGAATGAATACGGGTCCATCAATCACACGTACCACCTGGATGTTGTGGAGCGATCGCCTCACCGGCCCATCCTCCAAGCCGGACTGCCGGCAAATGCCTCCACAGTGGTCGGAGGAGACGTAGAGTTTGTCTGCAAGGTTTACAGTGATGCCCAGCCCCACATCCAGTGGATCAAGCACGTGGAAAAGAACGGCAGTAAATACGGGCCCGACGGGCTGCCCTACCTCAAGGTTCTCAAGGCCGCCGGTGTTAACACCACGGACAAAGAGATTGAGGTTCTCTATATTCGGAATGTAACTTTTGAGGACGCTGGGGAATATACGTGCTTGGCGGGTAATTCTATTGGGATATCCTTTCACTCTGCATGGTTGACAGTTCTGCCAGCGCCTGGAAGAGAAAAGGAGATTACAGCTTCCCCAGACTACCTGGAGATAGCCATTTACTGCATAGGGGTCTTCTTAATCGCCTGTATGGTGGTAACAGTCATCCTGTGCCGAATGAAGAACACGACCAAGAAGCCAGACTTCAGCAGCCAGCCGGCTGTGCACAAGCTGACCAAACGTATCCCCCTGCGGAGACAGGTATCACTGGAGTCCAGCTCCTCCATGAACTCCAACACCCCGCTGGTGAGGATAACAACACGCCTCTCTTCAACGGCAGACACCCCCATGCTGGCAGGGGTCTCCGAGTATGAACTTCCAGAGGACCCAAAATGGGAGTTTCCAAGAGATAAGCTGACACTGGGCAAGCCCCTGGGAGAAGGTTGCTTTGGGCAAGTGGTCATGGCGGAAGCAGTGGGAATTGACAAAGACAAGCCCAAGGAGGCGGTCACCGTGGCCGTGAAGATGTTGAAAGATGATGCCACAGAGAAAGACCTTTCTGATCTGGTGTCAGAGATGGAGATGATGAAGATGATTGGGAAACACAAGAATATCATAAATCTTCTTGGAGCCTGCACACAGGATGGGCCTCTCTATGTCATAGTTGAGTATGCCTCTAAAGGCAACCTCCGAGAATACCTCCGAGCCCGGAGGCCACCCGGGATGGAGTACTCCTATGACATTAACCGTGTTCCTGAGGAGCAGATGACCTTCAAGGACTTGGTGTCATGCACCTACCAGCTGGCCAGAGGCATGGAGTACTTGGCTTCCCAAAAATGTATTCATCGAGATTTAGCAGCCAGAAATGTTTTGGTAACAGAAAACAATGTGATGAAAATAGCAGACTTTGGACTCGCCAGAGATATCAACAATATAGACTATTACAAAAAGACCACCAATGGGCGGCTTCCAGTCAAGTGGATGGCTCCAGAAGCCCTGTTTGATAGAGTATACACTCATCAGAGTGATGTCTGGTCCTTCGGGGTGTTAATGTGGGAGATCTTCACTTTAGGGGGCTCGCCCTACCCAGGGATTCCCGTGGAGGAACTTTTTAAGCTGCTGAAGGAAGGACACAGAATGGATAAGCCAGCCAACTGCACCAACGAACTGTACATGATGATGAGGGACTGTTGGCATGCAGTGCCCTCCCAGAGACCAACGTTCAAGCAGTTGGTAGAAGACTTGGATCGAATTCTCACTCTCACAACCAATGAGGAATACTTGGACCTCAGCCAACCTCTCGAACAGTATTCACCTAGTTACCCTGACACAAGAAGTTCTTGTTCTTCAGGAGATGATTCTGTTTTTTCTCCAGACCCCATGCCTTACGAACCATGCCTTCCTCAGTATCCACACATAAACGGCAGTGTTAAAACAAAGGGTCAAGACAATTCTGCAGATATCCAGCACAGTGGCGGCCGCTCGAGTCTAGAGGGCCCGCGGTTCGAAGGTAAGCCTATCCCTAACCCTCTCCTCGGTCTCGATTCTACGCGTACCGGTCATCATCACCATCACCATTGA

Receptor sequence

V5 tag

6x His

pcDNA3.1-FGFR2::R4L2:

TCTCTGGCTAACTAGAGAACCCACTGCTTACTGGCTTATCGAAATTAATACGACTCACTATAGGGAGACCCAAGCTGGCTAGTTAAGCTTGGTACCGAGCTCGGATCCAGTACCCTTCACCATGGTCAGCTGGGGTCGTTTCATCTGCCTGGTCGTGGTCACCATGGCAACCTTGTCCCTGGCCCGGCCCTCCTTCAGTTTAGTTGAGGATACCACATTAGAGCCAGAAGAGCCACCAACCAAATACCAAATCTCTCAACCAGAAGTGTACGTGGCTGCGCCAGGGGAGTCGCTAGAGGTGCGCTGCCTGTTGAAAGATGCCGCCGTGATCAGTTGGACTAAGGATGGGGTGCACTTGGGGCCCAACAATAGGACAGTGCTTATTGGGGAGTACTTGCAGATAAAGGGCGCCACGCCTAGAGACTCCGGCCTCTATGCTTGTACTGCCAGTAGGACTGTAGACAGTGAAACTTGGTACTTCATGGTGAATGTCACAGATGCCATCTCATCCGGAGATGATGAGGATGACACCGATGGTGCGGAAGATTTTGTCAGTGAGAACAGTAACAACAAGAGAGCACCATACTGGACCAACACAGAAAAGATGGAAAAGCGGCTCCATGCTGTGCCTGCGGCCAACACTGTCAAGTTTCGCTGCCCAGCCGGGGGGAACCCAATGCCAACCATGCGGTGGCTGAAAAACGGGAAGGAGTTTAAGCAGGAGCATCGCATTGGAGGCTACAAGGTACGAAACCAGCACTGGAGCCTCATTATGGAAAGTGTGGTCCCATCTGACAAGGGAAATTATACCTGTGTAGTGGAGAATGAATACGGGTCCATCAATCACACGTACCACCTGGATGTTGTGGAGCGATCGCCTCACCGGCCCATCCTCCAAGCCGGACTGCCGGCAAATGCCTCCACAGTGGTCGGAGGAGACGTAGAGTTTGTCTGCAAGGTTTACAGTGATGCCCAGCCCCACATCCAGTGGATCAAGCACGTGGAAAAGAACGGCAGTAAATACGGGCCCGACGGGCTGCCCTACCTCAAGGTTCTCAAGGCCGCCGGTGTTAACACCACGGACAAAGAGATTGAGGTTCTCTATATTCGGAATGTAACTTTTGAGGACGCTGGGGAATATACGTGCTTGGCGGGTAATTCTATTGGGATATCCTTTCACTCTGCATGGTTGACAGTTCTGCCAGCGCCTGGAAGAGAAAAGGAGATTACAGCTTCCCCAGACTACCTGGAGATAGCCATTTACTGCATAGGGGTCTTCTTAATCGCCTGTATGGTGGTAACAGTCATCCTGTGCCGAATGAAGAACACGACCAAGAAGCCAGACTTCAGCAGCCAGCCGGCTGTGCACAAGCTGACCAAACGTATCCCCCTGCGGAGACAGTTCTCACTGGAGTCCAGCTCCTCCATGAACTCCAACACCCCGCTGGTGAGGATAACAACACGCCTCTCTTCAACGGCAGACACCCCCATGCTGGCAGGGGTCTCCGAGTATGAACTTCCAGAGGACCCAAAATGGGAGTTTCCAAGAGATAAGCTGACACTGGGCAAGCCCCTGGGAGAAGGTTGCTTTGGGCAAGTGGTCATGGCGGAAGCAGTGGGAATTGACAAAGACAAGCCCAAGGAGGCGGTCACCGTGGCCGTGAAGATGTTGAAAGATGATGCCACAGAGAAAGACCTTTCTGATCTGGTGTCAGAGATGGAGATGATGAAGATGATTGGGAAACACAAGAATATCATAAATCTTCTTGGAGCCTGCACACAGGATGGGCCTCTCTATGTCATAGTTGAGTATGCCTCTAAAGGCAACCTCCGAGAATACCTCCGAGCCCGGAGGCCACCCGGGATGGAGTACTCCTATGACATTAACCGTGTTCCTGAGGAGCAGATGACCTTCAAGGACTTGGTGTCATGCACCTACCAGCTGGCCAGAGGCATGGAGTACTTGGCTTCCCAAAAATGTATTCATCGAGATTTAGCAGCCAGAAATGTTTTGGTAACAGAAAACAATGTGATGAAAATAGCAGACTTTGGACTCGCCAGAGATATCAACAATATAGACTATTACAAAAAGACCACCAATGGGCGGCTTCCAGTCAAGTGGATGGCTCCAGAAGCCCTGTTTGATAGAGTATACACTCATCAGAGTGATGTCTGGTCCTTCGGGGTGTTAATGTGGGAGATCTTCACTTTAGGGGGCTCGCCCTACCCAGGGATTCCCGTGGAGGAACTTTTTAAGCTGCTGAAGGAAGGACACAGAATGGATAAGCCAGCCAACTGCACCAACGAACTGTACATGATGATGAGGGACTGTTGGCATGCAGTGCCCTCCCAGAGACCAACGTTCAAGCAGTTGGTAGAAGACTTGGATCGAATTCTCACTCTCACAACCAATGAGGAATACTTGGACCTCAGCCAACCTCTCGAACAGTATTCACCTAGTTACCCTGACACAAGAAGTTCTTGTTCTTCAGGAGATGATTCTGTTTTTTCTCCAGACCCCATGCCTTACGAACCATGCCTTCCTCAGTATCCACACATAAACGGCAGTGTTAAAACAAAGGGTCAAGACAATTCTGCAGATATCCAGCACAGTGGCGGCCGCTCGAGTCTAGAGGGCCCGCGGTTCGAAGGTAAGCCTATCCCTAACCCTCTCCTCGGTCTCGATTCTACGCGTACCGGTCATCATCACCATCACCATTGA

Receptor sequence

V5 tag

6x His

pcDNA3.1-FGFR3::R2L2:

TCTCTGGCTAACTAGAGAACCCACTGCTTACTGGCTTATCGAAATTAATACGACTCACTATAGGGAGACCCAAGCTGGCTAGTTAAGCTTGGTACCGAGCTCGGATCCAGTACCCTTCACCCACGACCCCACCATGGGCGCCCCTGCCTGCGCCCTCGCGCTCTGCGTGGCCGTGGCCATCGTGGCCGGCGCCTCCTCGGAGTCCTTGGGGACGGAGCAGCGCGTCGTGGGGCGAGCGGCAGAAGTCCCGGGCCCAGAGCCCGGCCAGCAGGAGCAGTTGGTCTTCGGCAGCGGGGATGCTGTGGAGCTGAGCTGTCCCCCGCCCGGGGGTGGTCCCATGGGGCCCACTGTCTGGGTCAAGGATGGCACAGGGCTGGTGCCCTCGGAGCGTGTCCTGGTGGGGCCCCAGCGGCTGCAGGTGCTGAATGCCTCCCACGAGGACTCCGGGGCCTACAGCTGCCGGCAGCGGCTCACGCAGCGCGTACTGTGCCACTTCAGTGTGCGGGTGACAGACGCTCCATCCTCGGGAGATGACGAAGACGGGGAGGACGAGGCTGAGGACACAGGTGTGGACACAGGGGCCCCTTACTGGACACGGCCCGAGCGGATGGACAAGAAGCTGCTGGCCGTGCCGGCCGCCAACACCGTCCGCTTCCGCTGCCCAGCCGCTGGCAACCCCACTCCCTCCATCTCCTGGCTGAAGAACGGCAGGGAGTTCCGCGGCGAGCACCGCATTGGAGGCATCAAGCTGCGGCATCAGCAGTGGAGCCTGGTCATGGAAAGCGTGGTGCCCTCGGACCGCGGCAACTACACCTGCGTCGTGGAGAACAAGTTTGGCAGCATCCGGCAGACGTACACGCTGGACGTGCTGGAGCGCTCCCCGCACCGGCCCATCCTGCAGGCGGGGCTGCCGGCCAACCAGACGGCGGTGCTGGGCAGCGACGTGGAGTTCCACTGCAAGGTGTACAGTGACGCACAGCCCCACATCCAGTGGCTCAAGCACGTGGAGGTGAATGGCAGCAAGGTGGGCCCGGACGGCACACCCTACGTTACCGTGCTCAAGACGGCGGGCGCTAACACCACCGACAAGGAGCTAGAGGTTCTCTCCTTGCACAACGTCACCTTTGAGGACGCCGGGGAGTACACCTGCCTGGCGGGCAATTCTATTGGGTTTTCTCATCACTCTGCGTGGCTGGTGGTGCTGCCAGCCGAGGAGGAGCTGGTGGAGGCTGACGAGGCGGGCAGTGTGTATGCAGGCATCCTCAGCTACGGGGTGGGCTTCTTCCTGTTCATCCTGGTGGTGGCGGCTGTGACGCTCTGCCGCCTGCGCAGCCCCCCCAAGAAAGGCCTGGGCTCCCCCACCGTGCACAAGATCTCCCGCTTCCCGCTCAAGCGACAGGTGacagtttcggctGAGTCCAACGCGTCCATGAGCTCCAACACACCACTGGTGCGCATCGCAAGGCTGTCCTCAGGGGAGGGCCCCACGCTGGCCAATGTCTCCGAGCTCGAGCTGCCTGCCGACCCCAAATGGGAGCTGTCTCGGGCCCGGCTGACCCTGGGCAAGCCCCTTGGGGAGGGCTGCTTCGGCCAGGTGGTCATGGCGGAGGCCATCGGCATTGACAAGGACCGGGCCGCCAAGCCTGTCACCGTAGCCGTGAAGATGCTGAAAGACGATGCCACTGACAAGGACCTGTCGGACCTGGTGTCTGAGATGGAGATGATGAAGATGATCGGGAAACACAAAAACATCATCAACCTGCTGGGCGCCTGCACGCAGGGCGGGCCCCTGTACGTGCTGGTGGAGTACGCGGCCAAGGGTAACCTGCGGGAGTTTCTGCGGGCGCGGCGGCCCCCGGGCCTGGACTACTCCTTCGACACCTGCAAGCCGCCCGAGGAGCAGCTCACCTTCAAGGACCTGGTGTCCTGTGCCTACCAGGTGGCCCGGGGCATGGAGTACTTGGCCTCCCAGAAGTGCATCCACAGGGACCTGGCTGCCCGCAATGTGCTGGTGACCGAGGACAACGTGATGAAGATCGCAGACTTCGGGCTGGCCCGGGACGTGCACAACCTCGACTACTACAAGAAGACGACCAACGGCCGGCTGCCCGTGAAGTGGATGGCGCCTGAGGCCTTGTTTGACCGAGTCTACACTCACCAGAGTGACGTCTGGTCCTTTGGGGTCCTGCTCTGGGAGATCTTCACGCTGGGGGGCTCCCCGTACCCCGGCATCCCTGTGGAGGAGCTCTTCAAGCTGCTGAAGGAGGGCCACCGCATGGACAAGCCCGCCAACTGCACACACGACCTGTACATGATCATGCGGGAGTGCTGGCATGCCGCGCCCTCCCAGAGGCCCACCTTCAAGCAGCTGGTGGAGGACCTGGACCGTGTCCTTACCGTGACGTCCACCGACGAGTACCTGGACCTGTCGGCGCCTTTCGAGCAGTACTCCCCGGGTGGCCAGGACACCCCCAGCTCCAGCTCCTCAGGGGACGACTCCGTGTTTGCCCACGACCTGCTGCCCCCGGCCCCACCCAGCAGTGGGGGCTCGCGGACAAAGGGTCAAGACAATTCTGCAGATATCCAGCACAGTGGCGGCCGCTCGAGTCTAGAGGGCCCGCGGTTCGAAGGTAAGCCTATCCCTAACCCTCTCCTCGGTCTCGATTCTACGCGTACCGGTCATCATCACCATCACCATTGA

Receptor sequence

V5 tag

6x His

pcDNA3.1-FGFR4::R2L2:

TCTCTGGCTAACTAGAGAACCCACTGCTTACTGGCTTATCGAAATTAATACGACTCACTATAGGGAGACCCAAGCTGGCTAGTTAAGCTTGGTACCGAGCTCGGATCCAGTACCCTTCACCTGAGAGCTGTGAGAAGGAGATGCGGCTGCTGCTGGCCCTGTTGGGGGTCCTGCTGAGTGTGCCTGGGCCTCCAGTCTTGTCCCTGGAGGCCTCTGAGGAAGTGGAGCTTGAGCCCTGCCTGGCTCCCAGCCTGGAGCAGCAAGAGCAGGAGCTGACAGTAGCCCTTGGaCAGCCTGTGCGgCTGTGCTGTGGGCGGGCTGAGCGTGGTGGCCACTGGTACAAGGAGGGCAGTCGCCTGGCACCTGCTGGCCGTGTACGGGGCTGGAGGGGCCGCCTAGAGATTGCCAGCTTCCTACCTGAGGATGCTGGCCGCTACCTCTGCCTGGCACGAGGCTCCATGATCGTCCTGCAGAATCTCACCTTGATTACAGGTGACTCCTTGACCTCCAGCAACGATGATGAGGACCCCAAGTCCCATAGGGACCCCTCGAATAGGCACAGTTACCCCCAGCAAGCACCCTACTGGACACACCCCCAGCGCATGGAGAAGAAACTGCATGCAGTACCTGCGGGGAACACCGTCAAGTTCCGCTGTCCAGCTGCAGGCAACCCCACGCCCACCATCCGCTGGCTTAAGGATGGACAGGCCTTTCATGGGGAGAACCGCATTGGAGGCATTCGGCTGCGCCATCAGCACTGGAGTCTCGTGATGGAGAGCGTGGTGCCCTCGGACCGCGGCACATACACCTGCCTGGTAGAGAACGCTGTGGGCAGCATCCGCTATAACTACCTGCTAGATGTGCTGGAGCGGTCCCCGCACCGGCCCATCCTGCAGGCCGGGCTCCCGGCCAACACCACAGCCGTGGTGGGCAGCGACGTGGAGCTGCTGTGCAAGGTGTACAGCGATGCCCAGCCCCACATCCAGTGGCTGAAGCACATCGTCATCAACGGCAGCAGCTTCGGAGCCGACGGTTTCCCCTATGTGCAAGTCCTAAAGACTGCAGACATCAATAGCTCAGAGGTGGAGGTCCTGTACCTGCGGAACGTGTCAGCCGAGGACGCAGGCGAGTACACCTGCCTCGCAGGCAATTCCATCGGCCTCTCCTACCAGTCTGCCTGGCTCACGGTGCTGCCAGAGGAGGACCCCACATGGACCGCAGCAGCGCCCGAGGCCAGGTATACGGACATCATCCTGTACGCGTCGGGCTCCCTGGCCTTGGCTGTGCTCCTGCTGCTGGCCGGGCTGTATCGAGGGCAGGCGCTCCACGGCCGGCACCCCCGCCCGCCCGCCACTGTGCAGAAGCTCTCCCGCTTCCCTCTGGCCCGACAGgtaacagtttcggctGAGTCAGGCTCTTCCGGCAAGTCAAGCTCATCCCTGGTACGAGGCGTGCGTCTCTCCTCCAGCGGCCCCGCCTTGCTCGCCGGCCTCGTGAGTCTAGATCTACCTCTCGACCCACTATGGGAGTTCCCCCGGGACAGGCTGGTGCTTGGGAAGCCCCTAGGCGAGGGCTGCTTTGGCCAGGTAGTACGTGCAGAGGCCTTTGGCATGGACCCTGCCCGGCCTGACCAAGCCAGCACTGTGGCCGTCAAGATGCTCAAAGACAACGCCTCTGACAAGGACCTGGCCGACCTGGTCTCGGAGATGGAGGTGATGAAGCTGATCGGCCGACACAAGAACATCATCAACCTGCTTGGTGTCTGCACCCAGGAAGGGCCCCTGTACGTGATCGTGGAGTGCGCCGCCAAGGGAAACCTGCGGGAGTTCCTGCGGGCCCGGCGCCCCCCAGGCCCCGACCTCAGCCCCGACGGTCCTCGGAGCAGTGAGGGGCCGCTCTCCTTCCCAGTCCTGGTCTCCTGCGCCTACCAGGTGGCCCGAGGCATGCAGTATCTGGAGTCCCGGAAGTGTATCCACCGGGACCTGGCTGCCCGCAATGTGCTGGTGACTGAGGACAATGTGATGAAGATTGCTGACTTTGGGCTGGCCCGCGGCGTCCACCACATTGACTACTATAAGAAAACCAGCAACGGCCGCCTGCCTGTGAAGTGGATGGCGCCCGAGGCCTTGTTTGACCGGGTGTACACACACCAGAGTGACGTGTGGTCTTTTGGGATCCTGCTATGGGAGATCTTCACCCTCGGGGGCTCCCCGTATCCTGGCATCCCGGTGGAGGAGCTGTTCTCGCTGCTGCGGGAGGGACATCGGATGGACCGACCCCCACACTGCCCCCCAGAGCTGTACGGGCTGATGCGTGAGTGCTGGCACGCAGCGCCCTCCCAGAGGCCTACCTTCAAGCAGCTGGTGGAGGCGCTGGACAAGGTCCTGCTGGCCGTCTCTGAGGAGTACCTCGACCTCCGCCTGACCTTCGGACCCTATTCCCCCTCTGGTGGGGACGCCAGCAGCACCTGCTCCTCCAGCGATTCTGTCTTCAGCCACGACCCCCTGCCATTGGGATCCAGCTCCTTCCCCTTCGGGTCTGGGGTGCAGACAAAGGGTCAAGACAATTCTGCAGATATCCAGCACAGTGGCGGCCGCTCGAGTCTAGAGGGCCCGCGGTTCGAAGGTAAGCCTATCCCTAACCCTCTCCTCGGTCTCGATTCTACGCGTACCGGTCATCATCACCATCACCATTGA

Receptor sequence

V5 tag

6x His

pcDNA3.1-Fgfr2:

TCTCTGGCTAACTAGAGAACCCACTGCTTACTGGCTTATCGAAATTAATACGACTCACTATAGGGAGACCCAAGCTGGCTAGCACTAGTAAGCTTGGTACCGAGCTCGGATCCAGTACCCTTCACCATGTTCGCCCGAGGGTGGCTTCTGGGTGCTCTCCTGCTAATGACCCTGGCAACGGTATCTGTGGCACGGCCGTCATTGAAGATTGACTTGGTGAATACATCTGCACCTGAAGAGCCTCCAACCAAAAACCAAAACTGTGTGCCCGTCCTGTTCTCGGTGCACCCGGGTGAGCTGCTCAAGCTCAAGTGCCCGCTGTCCGGGGCAGATGACGTCGTCTGGACCAAAGACAGCTCCTCGCTGCGCCCCGACAACCGCACGCTCGTAGCTCGGGATTGGCTGCAGATCAGCGACGCCACCCCGAAGGACTCGGGCCTGTATTCGTGCAGTGCCACGGGCTTGCGGGACTGCGATGTGTTTAGCTTCATCGTAAACGTTACAGATGCCATCTCATCCGGTGACGATGAGGACGACACAGAGCGGTCAGACGATGTTGGGGCAGACGGCGAACAAATGCGGCTGCCATATTGGACCTTTCCAGAGAAGATGGAGAAGAAGCTGCATGCCGTCCCGGCGGCCAACACGGTGAAGTTTCGCTGCGCGGCAGCCGGAAATCCTAAACCCAAAATGCGCTGGCTGAAAAACGCAAAACCCTTCAGACAGGAGGATCGCATGGGCGGCTACAAGGTTCGACTCCAGCACTGGACCCTGATCATGGAGAGCGTGGTTCCCTCTGATAAAGGAAACTACACCTGTCTGGTGGAGAACCAGTATGGCTCCATCGATCACACATACACGCTGGATGTCGTTGAACGCTCTCCTCACCGGCCCATTCTCCAGGCAGGCCTGCCAGCAAACGTCACTGTGCAAGTTGGACAAGATGCGAAGTTCGTCTGTAAAGTTTACAGTGATGCTCAGCCTCATATCCAGTGGTTGCAGCACTATACGAAGAACGGCAGCTGCTGTGGTCCTGATGGACTTCCTTATGTCCGCGTATTGAAGACGGCAGGTGTGAACACTACGGATAAAGAGATCGAGGTGCTCTACCTGCCTAATGTAACTTTCGAGGATGCGGGCGAGTATACGTGTTTGGCGGGTAACTCTATTGGGATCTCCTATCACACTGCTTGGTTGACGGTGCATCCAGCTGAGACGAATCCCATCGAGACGGATTATCCTCCAGATTATGTGGAGATCGCCATCTACTGTATAGGTGTGTTTCTGATCGCCTGTATGGTGGTGATCGTGGTGGTTTGCCGGATGAGGACGTCGGCTAAGAAGCCTGATTTCAGCAGTCAGCCAGCAGTGCACAAGCTCACCAAACAGATCCCCCTGCGCCGCCAGGTAACAGTGTCGTCAGACTCCAGCTCCTCCATGAGCTCTAGCACGCCATTGGTCAGAATCACCACTCGCCGGAGCTCCGCCCACGATGATCCCATTCCAGAATACGACCTCCCTGAGGACCCACGCTGGGAGTTTTCTAGAGACAAGTTAACTCTGGGTAAGCCTCTCGGCGAGGGCTGCTTTGGTCAGGTTGTGATGGCTGAAGCTCTTGGCATTGACAAGGACAAACCGAAAGAGGCTGTGACTGTGGCTGTCAAGATGCTAAAAGATGATGCGACCGAGAAGGATCTTTCTGACCTGGTGTCAGAGATGGAGATGATGAAGATGATTGGCCGGCACAAGAACATCATCAATCTGTTGGGAGCGTGCACACAGGATGGTCCATTATATGTCATTGTTGAGTATGCATCTAAAGGGAACCTCCGGGAGTATCTGAGGGCCCGGCGGCCCCCAGGAATGGAGTATTCCTATGATATAGCCCGCGTATCAGATGAGCCTCTCACTTTCAAAGATCTGGTCTCCTGCACTTATCAAGTTGCCCGTGGCATGGAGTATTTAGCCTCTCAGAAGTGTATACACCGAGATTTGGCCGCGAGGAATGTGTTGGTCACGGAGAGCAACGTTATGAAGATCGCAGACTTCGGTCTGGCCAGAGACGTCCACAACATAGATTACTATAAAAAGACCACAAATGGTCGTCTGCCTGTGAAATGGATGGCTCCAGAAGCACTGTTCGACCGAGTTTATACACACCAGAGTGACGTCTGGTCGTTTGGGGTTTTAATGTGGGAGATCTTCACTCTCGGCGGTTCTCCGTATCCTGGAATACCTGTCGAGGAGCTCTTTAAACTATTAAAAGAGGGTCATCGCATGGACAAACCTGCTAACTGCACCAATGAGTTGTACATGATGATGAAGGACTGCTGGCATGCCATCTCCTCCCACAGACCCACATTCAAGCAGTTGGTGGAGGATCTGGACCGCATTCTCACTCTGGCAACCAACGAGGAATATCTGGACCTGTGTGCCCCGGTGGAGCAATACTCTCCCAGCTTCCCCGACACACGCAGCTCCTGCCCCTCCGGAGACGACTCCGTATTTTCCCATGATCCCTTAGCGGACGAGCCCTGCCTTCCCAAATACCAGCACATCAACGGCGGCATAAAAACAGCCAAGGGTCAAGACAATTCTGCAGATATCCAGCACAGTGGCGGCCGCTCGAGTCTAGAGGGCCCGCGGTTCGAAGGTAAGCCTATCCCTAACCCTCTCCTCGGTCTCGATTCTACGCGTACCGGTCATCATCACCATCACCATTGA

Receptor sequence

V5 tag

6x His

pcDNA3.1-FGFR2::fL2:

TCTCTGGCTAACTAGAGAACCCACTGCTTACTGGCTTATCGAAATTAATACGACTCACTATAGGGAGACCCAAGCTGGCTAGTTAAGCTTGGTACCGAGCTCGGATCCAGTACCCTTCACCATGGTCAGCTGGGGTCGTTTCATCTGCCTGGTCGTGGTCACCATGGCAACCTTGTCCCTGGCCCGGCCCTCCTTCAGTTTAGTTGAGGATACCACATTAGAGCCAGAAGAGCCACCAACCAAATACCAAATCTCTCAACCAGAAGTGTACGTGGCTGCGCCAGGGGAGTCGCTAGAGGTGCGCTGCCTGTTGAAAGATGCCGCCGTGATCAGTTGGACTAAGGATGGGGTGCACTTGGGGCCCAACAATAGGACAGTGCTTATTGGGGAGTACTTGCAGATAAAGGGCGCCACGCCTAGAGACTCCGGCCTCTATGCTTGTACTGCCAGTAGGACTGTAGACAGTGAAACTTGGTACTTCATGGTGAATGTCACAGATGCCATCTCATCCGGAGATGATGAGGATGACACCGATGGTGCGGAAGATTTTGTCAGTGAGAACAGTAACAACAAGAGAGCACCATACTGGACCAACACAGAAAAGATGGAAAAGCGGCTCCATGCTGTGCCTGCGGCCAACACTGTCAAGTTTCGCTGCCCAGCCGGGGGGAACCCAATGCCAACCATGCGGTGGCTGAAAAACGGGAAGGAGTTTAAGCAGGAGCATCGCATTGGAGGCTACAAGGTACGAAACCAGCACTGGAGCCTCATTATGGAAAGTGTGGTCCCATCTGACAAGGGAAATTATACCTGTGTAGTGGAGAATGAATACGGGTCCATCAATCACACGTACCACCTGGATGTTGTGGAGCGATCGCCTCACCGGCCCATCCTCCAAGCCGGACTGCCGGCAAATGCCTCCACAGTGGTCGGAGGAGACGTAGAGTTTGTCTGCAAGGTTTACAGTGATGCCCAGCCCCACATCCAGTGGATCAAGCACGTGGAAAAGAACGGCAGTAAATACGGGCCCGACGGGCTGCCCTACCTCAAGGTTCTCAAGGCCGCCGGTGTTAACACCACGGACAAAGAGATTGAGGTTCTCTATATTCGGAATGTAACTTTTGAGGACGCTGGGGAATATACGTGCTTGGCGGGTAATTCTATTGGGATATCCTTTCACTCTGCATGGTTGACAGTTCTGCCAGCGCCTGGAAGAGAAAAGGAGATTACAGCTTCCCCAGACTACCTGGAGATAGCCATTTACTGCATAGGGGTCTTCTTAATCGCCTGTATGGTGGTAACAGTCATCCTGTGCCGAATGAAGAACACGACCAAGAAGCCAGACTTCAGCAGCCAGCCGGCTGTGCACAAGCTGACCAAACGTATCCCCCTGCGGAGACAGGTAACAGTTTCGAGTGATTCCAGCTCCTCCATGAACTCCAACACCCCGCTGGTGAGGATAACAACACGCCTCTCTTCAACGGCAGACACCCCCATGCTGGCAGGGGTCTCCGAGTATGAACTTCCAGAGGACCCAAAATGGGAGTTTCCAAGAGATAAGCTGACACTGGGCAAGCCCCTGGGAGAAGGTTGCTTTGGGCAAGTGGTCATGGCGGAAGCAGTGGGAATTGACAAAGACAAGCCCAAGGAGGCGGTCACCGTGGCCGTGAAGATGTTGAAAGATGATGCCACAGAGAAAGACCTTTCTGATCTGGTGTCAGAGATGGAGATGATGAAGATGATTGGGAAACACAAGAATATCATAAATCTTCTTGGAGCCTGCACACAGGATGGGCCTCTCTATGTCATAGTTGAGTATGCCTCTAAAGGCAACCTCCGAGAATACCTCCGAGCCCGGAGGCCACCCGGGATGGAGTACTCCTATGACATTAACCGTGTTCCTGAGGAGCAGATGACCTTCAAGGACTTGGTGTCATGCACCTACCAGCTGGCCAGAGGCATGGAGTACTTGGCTTCCCAAAAATGTATTCATCGAGATTTAGCAGCCAGAAATGTTTTGGTAACAGAAAACAATGTGATGAAAATAGCAGACTTTGGACTCGCCAGAGATATCAACAATATAGACTATTACAAAAAGACCACCAATGGGCGGCTTCCAGTCAAGTGGATGGCTCCAGAAGCCCTGTTTGATAGAGTATACACTCATCAGAGTGATGTCTGGTCCTTCGGGGTGTTAATGTGGGAGATCTTCACTTTAGGGGGCTCGCCCTACCCAGGGATTCCCGTGGAGGAACTTTTTAAGCTGCTGAAGGAAGGACACAGAATGGATAAGCCAGCCAACTGCACCAACGAACTGTACATGATGATGAGGGACTGTTGGCATGCAGTGCCCTCCCAGAGACCAACGTTCAAGCAGTTGGTAGAAGACTTGGATCGAATTCTCACTCTCACAACCAATGAGGAATACTTGGACCTCAGCCAACCTCTCGAACAGTATTCACCTAGTTACCCTGACACAAGAAGTTCTTGTTCTTCAGGAGATGATTCTGTTTTTTCTCCAGACCCCATGCCTTACGAACCATGCCTTCCTCAGTATCCACACATAAACGGCAGTGTTAAAACAAAGGGTCAAGACAATTCTGCAGATATCCAGCACAGTGGCGGCCGCTCGAGTCTAGAGGGCCCGCGGTTCGAAGGTAAGCCTATCCCTAACCCTCTCCTCGGTCTCGATTCTACGCGTACCGGTCATCATCACCATCACCATTGA

Receptor sequence

V5 tag

6x His

pcDNA3.1-FGFR3::fL2:

TCTCTGGCTAACTAGAGAACCCACTGCTTACTGGCTTATCGAAATTAATACGACTCACTATAGGGAGACCCAAGCTGGCTAGTTAAGCTTGGTACCGAGCTCGGATCCAGTACCCTTCACCCACGACCCCACCATGGGCGCCCCTGCCTGCGCCCTCGCGCTCTGCGTGGCCGTGGCCATCGTGGCCGGCGCCTCCTCGGAGTCCTTGGGGACGGAGCAGCGCGTCGTGGGGCGAGCGGCAGAAGTCCCGGGCCCAGAGCCCGGCCAGCAGGAGCAGTTGGTCTTCGGCAGCGGGGATGCTGTGGAGCTGAGCTGTCCCCCGCCCGGGGGTGGTCCCATGGGGCCCACTGTCTGGGTCAAGGATGGCACAGGGCTGGTGCCCTCGGAGCGTGTCCTGGTGGGGCCCCAGCGGCTGCAGGTGCTGAATGCCTCCCACGAGGACTCCGGGGCCTACAGCTGCCGGCAGCGGCTCACGCAGCGCGTACTGTGCCACTTCAGTGTGCGGGTGACAGACGCTCCATCCTCGGGAGATGACGAAGACGGGGAGGACGAGGCTGAGGACACAGGTGTGGACACAGGGGCCCCTTACTGGACACGGCCCGAGCGGATGGACAAGAAGCTGCTGGCCGTGCCGGCCGCCAACACCGTCCGCTTCCGCTGCCCAGCCGCTGGCAACCCCACTCCCTCCATCTCCTGGCTGAAGAACGGCAGGGAGTTCCGCGGCGAGCACCGCATTGGAGGCATCAAGCTGCGGCATCAGCAGTGGAGCCTGGTCATGGAAAGCGTGGTGCCCTCGGACCGCGGCAACTACACCTGCGTCGTGGAGAACAAGTTTGGCAGCATCCGGCAGACGTACACGCTGGACGTGCTGGAGCGCTCCCCGCACCGGCCCATCCTGCAGGCGGGGCTGCCGGCCAACCAGACGGCGGTGCTGGGCAGCGACGTGGAGTTCCACTGCAAGGTGTACAGTGACGCACAGCCCCACATCCAGTGGCTCAAGCACGTGGAGGTGAATGGCAGCAAGGTGGGCCCGGACGGCACACCCTACGTTACCGTGCTCAAGACGGCGGGCGCTAACACCACCGACAAGGAGCTAGAGGTTCTCTCCTTGCACAACGTCACCTTTGAGGACGCCGGGGAGTACACCTGCCTGGCGGGCAATTCTATTGGGTTTTCTCATCACTCTGCGTGGCTGGTGGTGCTGCCAGCCGAGGAGGAGCTGGTGGAGGCTGACGAGGCGGGCAGTGTGTATGCAGGCATCCTCAGCTACGGGGTGGGCTTCTTCCTGTTCATCCTGGTGGTGGCGGCTGTGACGCTCTGCCGCCTGCGCAGCCCCCCCAAGAAAGGCCTGGGCTCCCCCACCGTGCACAAGATCTCCCGCTTCCCGCTCAAGCGACAGGTGACAGTTTCGAGTGATTCCAACGCGTCCATGAGCTCCAACACACCACTGGTGCGCATCGCAAGGCTGTCCTCAGGGGAGGGCCCCACGCTGGCCAATGTCTCCGAGCTCGAGCTGCCTGCCGACCCCAAATGGGAGCTGTCTCGGGCCCGGCTGACCCTGGGCAAGCCCCTTGGGGAGGGCTGCTTCGGCCAGGTGGTCATGGCGGAGGCCATCGGCATTGACAAGGACCGGGCCGCCAAGCCTGTCACCGTAGCCGTGAAGATGCTGAAAGACGATGCCACTGACAAGGACCTGTCGGACCTGGTGTCTGAGATGGAGATGATGAAGATGATCGGGAAACACAAAAACATCATCAACCTGCTGGGCGCCTGCACGCAGGGCGGGCCCCTGTACGTGCTGGTGGAGTACGCGGCCAAGGGTAACCTGCGGGAGTTTCTGCGGGCGCGGCGGCCCCCGGGCCTGGACTACTCCTTCGACACCTGCAAGCCGCCCGAGGAGCAGCTCACCTTCAAGGACCTGGTGTCCTGTGCCTACCAGGTGGCCCGGGGCATGGAGTACTTGGCCTCCCAGAAGTGCATCCACAGGGACCTGGCTGCCCGCAATGTGCTGGTGACCGAGGACAACGTGATGAAGATCGCAGACTTCGGGCTGGCCCGGGACGTGCACAACCTCGACTACTACAAGAAGACGACCAACGGCCGGCTGCCCGTGAAGTGGATGGCGCCTGAGGCCTTGTTTGACCGAGTCTACACTCACCAGAGTGACGTCTGGTCCTTTGGGGTCCTGCTCTGGGAGATCTTCACGCTGGGGGGCTCCCCGTACCCCGGCATCCCTGTGGAGGAGCTCTTCAAGCTGCTGAAGGAGGGCCACCGCATGGACAAGCCCGCCAACTGCACACACGACCTGTACATGATCATGCGGGAGTGCTGGCATGCCGCGCCCTCCCAGAGGCCCACCTTCAAGCAGCTGGTGGAGGACCTGGACCGTGTCCTTACCGTGACGTCCACCGACGAGTACCTGGACCTGTCGGCGCCTTTCGAGCAGTACTCCCCGGGTGGCCAGGACACCCCCAGCTCCAGCTCCTCAGGGGACGACTCCGTGTTTGCCCACGACCTGCTGCCCCCGGCCCCACCCAGCAGTGGGGGCTCGCGGACGAAGGGTCAAGACAATTCTGCAGATATCCAGCACAGTGGCGGCCGCTCGAGTCTAGAGGGCCCGCGGTTCGAAGGTAAGCCTATCCCTAACCCTCTCCTCGGTCTCGATTCTACGCGTACCGGTCATCATCACCATCACCATTGA

Receptor sequence

V5 tag

6x His

pcDNA3.1-FGFR2-ΔTV:

TCTCTGGCTAACTAGAGAACCCACTGCTTACTGGCTTATCGAAATTAATACGACTCACTATAGGGAGACCCAAGCTGGCTAGTTAAGCTTGGTACCGAGCTCGGATCCAGTACCCTTCACCATGGTCAGCTGGGGTCGTTTCATCTGCCTGGTCGTGGTCACCATGGCAACCTTGTCCCTGGCCCGGCCCTCCTTCAGTTTAGTTGAGGATACCACATTAGAGCCAGAAGAGCCACCAACCAAATACCAAATCTCTCAACCAGAAGTGTACGTGGCTGCGCCAGGGGAGTCGCTAGAGGTGCGCTGCCTGTTGAAAGATGCCGCCGTGATCAGTTGGACTAAGGATGGGGTGCACTTGGGGCCCAACAATAGGACAGTGCTTATTGGGGAGTACTTGCAGATAAAGGGCGCCACGCCTAGAGACTCCGGCCTCTATGCTTGTACTGCCAGTAGGACTGTAGACAGTGAAACTTGGTACTTCATGGTGAATGTCACAGATGCCATCTCATCCGGAGATGATGAGGATGACACCGATGGTGCGGAAGATTTTGTCAGTGAGAACAGTAACAACAAGAGAGCACCATACTGGACCAACACAGAAAAGATGGAAAAGCGGCTCCATGCTGTGCCTGCGGCCAACACTGTCAAGTTTCGCTGCCCAGCCGGGGGGAACCCAATGCCAACCATGCGGTGGCTGAAAAACGGGAAGGAGTTTAAGCAGGAGCATCGCATTGGAGGCTACAAGGTACGAAACCAGCACTGGAGCCTCATTATGGAAAGTGTGGTCCCATCTGACAAGGGAAATTATACCTGTGTAGTGGAGAATGAATACGGGTCCATCAATCACACGTACCACCTGGATGTTGTGGAGCGATCGCCTCACCGGCCCATCCTCCAAGCCGGACTGCCGGCAAATGCCTCCACAGTGGTCGGAGGAGACGTAGAGTTTGTCTGCAAGGTTTACAGTGATGCCCAGCCCCACATCCAGTGGATCAAGCACGTGGAAAAGAACGGCAGTAAATACGGGCCCGACGGGCTGCCCTACCTCAAGGTTCTCAAGGCCGCCGGTGTTAACACCACGGACAAAGAGATTGAGGTTCTCTATATTCGGAATGTAACTTTTGAGGACGCTGGGGAATATACGTGCTTGGCGGGTAATTCTATTGGGATATCCTTTCACTCTGCATGGTTGACAGTTCTGCCAGCGCCTGGAAGAGAAAAGGAGATTACAGCTTCCCCAGACTACCTGGAGATAGCCATTTACTGCATAGGGGTCTTCTTAATCGCCTGTATGGTGGTAACAGTCATCCTGTGCCGAATGAAGAACACGACCAAGAAGCCAGACTTCAGCAGCCAGCCGGCTGTGCACAAGCTGACCAAACGTATCCCCCTGCGGAGACAGGTATCGGCTGAGTCCAGCTCCTCCATGAACTCCAACACCCCGCTGGTGAGGATAACAACACGCCTCTCTTCAACGGCAGACACCCCCATGCTGGCAGGGGTCTCCGAGTATGAACTTCCAGAGGACCCAAAATGGGAGTTTCCAAGAGATAAGCTGACACTGGGCAAGCCCCTGGGAGAAGGTTGCTTTGGGCAAGTGGTCATGGCGGAAGCAGTGGGAATTGACAAAGACAAGCCCAAGGAGGCGGTCACCGTGGCCGTGAAGATGTTGAAAGATGATGCCACAGAGAAAGACCTTTCTGATCTGGTGTCAGAGATGGAGATGATGAAGATGATTGGGAAACACAAGAATATCATAAATCTTCTTGGAGCCTGCACACAGGATGGGCCTCTCTATGTCATAGTTGAGTATGCCTCTAAAGGCAACCTCCGAGAATACCTCCGAGCCCGGAGGCCACCCGGGATGGAGTACTCCTATGACATTAACCGTGTTCCTGAGGAGCAGATGACCTTCAAGGACTTGGTGTCATGCACCTACCAGCTGGCCAGAGGCATGGAGTACTTGGCTTCCCAAAAATGTATTCATCGAGATTTAGCAGCCAGAAATGTTTTGGTAACAGAAAACAATGTGATGAAAATAGCAGACTTTGGACTCGCCAGAGATATCAACAATATAGACTATTACAAAAAGACCACCAATGGGCGGCTTCCAGTCAAGTGGATGGCTCCAGAAGCCCTGTTTGATAGAGTATACACTCATCAGAGTGATGTCTGGTCCTTCGGGGTGTTAATGTGGGAGATCTTCACTTTAGGGGGCTCGCCCTACCCAGGGATTCCCGTGGAGGAACTTTTTAAGCTGCTGAAGGAAGGACACAGAATGGATAAGCCAGCCAACTGCACCAACGAACTGTACATGATGATGAGGGACTGTTGGCATGCAGTGCCCTCCCAGAGACCAACGTTCAAGCAGTTGGTAGAAGACTTGGATCGAATTCTCACTCTCACAACCAATGAGGAATACTTGGACCTCAGCCAACCTCTCGAACAGTATTCACCTAGTTACCCTGACACAAGAAGTTCTTGTTCTTCAGGAGATGATTCTGTTTTTTCTCCAGACCCCATGCCTTACGAACCATGCCTTCCTCAGTATCCACACATAAACGGCAGTGTTAAAACAAAGGGTCAAGACAATTCTGCAGATATCCAGCACAGTGGCGGCCGCTCGAGTCTAGAGGGCCCGCGGTTCGAAGGTAAGCCTATCCCTAACCCTCTCCTCGGTCTCGATTCTACGCGTACCGGTCATCATCACCATCACCATTGA

Receptor sequence

V5 tag

6x His

pcDNA3.1-FGFR2::fL2-ΔTV:

TCTCTGGCTAACTAGAGAACCCACTGCTTACTGGCTTATCGAAATTAATACGACTCACTATAGGGAGACCCAAGCTGGCTAGTTAAGCTTGGTACCGAGCTCGGATCCAGTACCCTTCACCATGGTCAGCTGGGGTCGTTTCATCTGCCTGGTCGTGGTCACCATGGCAACCTTGTCCCTGGCCCGGCCCTCCTTCAGTTTAGTTGAGGATACCACATTAGAGCCAGAAGAGCCACCAACCAAATACCAAATCTCTCAACCAGAAGTGTACGTGGCTGCGCCAGGGGAGTCGCTAGAGGTGCGCTGCCTGTTGAAAGATGCCGCCGTGATCAGTTGGACTAAGGATGGGGTGCACTTGGGGCCCAACAATAGGACAGTGCTTATTGGGGAGTACTTGCAGATAAAGGGCGCCACGCCTAGAGACTCCGGCCTCTATGCTTGTACTGCCAGTAGGACTGTAGACAGTGAAACTTGGTACTTCATGGTGAATGTCACAGATGCCATCTCATCCGGAGATGATGAGGATGACACCGATGGTGCGGAAGATTTTGTCAGTGAGAACAGTAACAACAAGAGAGCACCATACTGGACCAACACAGAAAAGATGGAAAAGCGGCTCCATGCTGTGCCTGCGGCCAACACTGTCAAGTTTCGCTGCCCAGCCGGGGGGAACCCAATGCCAACCATGCGGTGGCTGAAAAACGGGAAGGAGTTTAAGCAGGAGCATCGCATTGGAGGCTACAAGGTACGAAACCAGCACTGGAGCCTCATTATGGAAAGTGTGGTCCCATCTGACAAGGGAAATTATACCTGTGTAGTGGAGAATGAATACGGGTCCATCAATCACACGTACCACCTGGATGTTGTGGAGCGATCGCCTCACCGGCCCATCCTCCAAGCCGGACTGCCGGCAAATGCCTCCACAGTGGTCGGAGGAGACGTAGAGTTTGTCTGCAAGGTTTACAGTGATGCCCAGCCCCACATCCAGTGGATCAAGCACGTGGAAAAGAACGGCAGTAAATACGGGCCCGACGGGCTGCCCTACCTCAAGGTTCTCAAGGCCGCCGGTGTTAACACCACGGACAAAGAGATTGAGGTTCTCTATATTCGGAATGTAACTTTTGAGGACGCTGGGGAATATACGTGCTTGGCGGGTAATTCTATTGGGATATCCTTTCACTCTGCATGGTTGACAGTTCTGCCAGCGCCTGGAAGAGAAAAGGAGATTACAGCTTCCCCAGACTACCTGGAGATAGCCATTTACTGCATAGGGGTCTTCTTAATCGCCTGTATGGTGGTAACAGTCATCCTGTGCCGAATGAAGAACACGACCAAGAAGCCAGACTTCAGCAGCCAGCCGGCTGTGCACAAGCTGACCAAACGTATCCCCCTGCGGAGACAGGTATCGAGTGATTCCAGCTCCTCCATGAACTCCAACACCCCGCTGGTGAGGATAACAACACGCCTCTCTTCAACGGCAGACACCCCCATGCTGGCAGGGGTCTCCGAGTATGAACTTCCAGAGGACCCAAAATGGGAGTTTCCAAGAGATAAGCTGACACTGGGCAAGCCCCTGGGAGAAGGTTGCTTTGGGCAAGTGGTCATGGCGGAAGCAGTGGGAATTGACAAAGACAAGCCCAAGGAGGCGGTCACCGTGGCCGTGAAGATGTTGAAAGATGATGCCACAGAGAAAGACCTTTCTGATCTGGTGTCAGAGATGGAGATGATGAAGATGATTGGGAAACACAAGAATATCATAAATCTTCTTGGAGCCTGCACACAGGATGGGCCTCTCTATGTCATAGTTGAGTATGCCTCTAAAGGCAACCTCCGAGAATACCTCCGAGCCCGGAGGCCACCCGGGATGGAGTACTCCTATGACATTAACCGTGTTCCTGAGGAGCAGATGACCTTCAAGGACTTGGTGTCATGCACCTACCAGCTGGCCAGAGGCATGGAGTACTTGGCTTCCCAAAAATGTATTCATCGAGATTTAGCAGCCAGAAATGTTTTGGTAACAGAAAACAATGTGATGAAAATAGCAGACTTTGGACTCGCCAGAGATATCAACAATATAGACTATTACAAAAAGACCACCAATGGGCGGCTTCCAGTCAAGTGGATGGCTCCAGAAGCCCTGTTTGATAGAGTATACACTCATCAGAGTGATGTCTGGTCCTTCGGGGTGTTAATGTGGGAGATCTTCACTTTAGGGGGCTCGCCCTACCCAGGGATTCCCGTGGAGGAACTTTTTAAGCTGCTGAAGGAAGGACACAGAATGGATAAGCCAGCCAACTGCACCAACGAACTGTACATGATGATGAGGGACTGTTGGCATGCAGTGCCCTCCCAGAGACCAACGTTCAAGCAGTTGGTAGAAGACTTGGATCGAATTCTCACTCTCACAACCAATGAGGAATACTTGGACCTCAGCCAACCTCTCGAACAGTATTCACCTAGTTACCCTGACACAAGAAGTTCTTGTTCTTCAGGAGATGATTCTGTTTTTTCTCCAGACCCCATGCCTTACGAACCATGCCTTCCTCAGTATCCACACATAAACGGCAGTGTTAAAACAAAGGGTCAAGACAATTCTGCAGATATCCAGCACAGTGGCGGCCGCTCGAGTCTAGAGGGCCCGCGGTTCGAAGGTAAGCCTATCCCTAACCCTCTCCTCGGTCTCGATTCTACGCGTACCGGTCATCATCACCATCACCATTGA

Receptor sequence

V5 tag

6x His

pcDNA3.1-L424A:R426A:

TCTCTGGCTAACTAGAGAACCCACTGCTTACTGGCTTATCGAAATTAATACGACTCACTATAGGGAGACCCAAGCTGGCTAGTTAAGCTTGGTACCGAGCTCGGATCCAGTACCCTTCACCATGGTCAGCTGGGGTCGTTTCATCTGCCTGGTCGTGGTCACCATGGCAACCTTGTCCCTGGCCCGGCCCTCCTTCAGTTTAGTTGAGGATACCACATTAGAGCCAGAAGAGCCACCAACCAAATACCAAATCTCTCAACCAGAAGTGTACGTGGCTGCGCCAGGGGAGTCGCTAGAGGTGCGCTGCCTGTTGAAAGATGCCGCCGTGATCAGTTGGACTAAGGATGGGGTGCACTTGGGGCCCAACAATAGGACAGTGCTTATTGGGGAGTACTTGCAGATAAAGGGCGCCACGCCTAGAGACTCCGGCCTCTATGCTTGTACTGCCAGTAGGACTGTAGACAGTGAAACTTGGTACTTCATGGTGAATGTCACAGATGCCATCTCATCCGGAGATGATGAGGATGACACCGATGGTGCGGAAGATTTTGTCAGTGAGAACAGTAACAACAAGAGAGCACCATACTGGACCAACACAGAAAAGATGGAAAAGCGGCTCCATGCTGTGCCTGCGGCCAACACTGTCAAGTTTCGCTGCCCAGCCGGGGGGAACCCAATGCCAACCATGCGGTGGCTGAAAAACGGGAAGGAGTTTAAGCAGGAGCATCGCATTGGAGGCTACAAGGTACGAAACCAGCACTGGAGCCTCATTATGGAAAGTGTGGTCCCATCTGACAAGGGAAATTATACCTGTGTAGTGGAGAATGAATACGGGTCCATCAATCACACGTACCACCTGGATGTTGTGGAGCGATCGCCTCACCGGCCCATCCTCCAAGCCGGACTGCCGGCAAATGCCTCCACAGTGGTCGGAGGAGACGTAGAGTTTGTCTGCAAGGTTTACAGTGATGCCCAGCCCCACATCCAGTGGATCAAGCACGTGGAAAAGAACGGCAGTAAATACGGGCCCGACGGGCTGCCCTACCTCAAGGTTCTCAAGGCCGCCGGTGTTAACACCACGGACAAAGAGATTGAGGTTCTCTATATTCGGAATGTAACTTTTGAGGACGCTGGGGAATATACGTGCTTGGCGGGTAATTCTATTGGGATATCCTTTCACTCTGCATGGTTGACAGTTCTGCCAGCGCCTGGAAGAGAAAAGGAGATTACAGCTTCCCCAGACTACCTGGAGATAGCCATTTACTGCATAGGGGTCTTCTTAATCGCCTGTATGGTGGTAACAGTCATCCTGTGCCGAATGAAGAACACGACCAAGAAGCCAGACTTCAGCAGCCAGCCGGCTGTGCACAAGCTGACCAAACGTATCCCCGCGCGGGCACAGGTAACAGTTTCGGCTGAGTCCAGCTCCTCCATGAACTCCAACACCCCGCTGGTGAGGATAACAACACGCGTAACAGTTTCGGCTGAGCTCTCTTCAACGGCAGACACCCCCATGCTGGCAGGGGTCTCCGAGTATGAACTTCCAGAGGACCCAAAATGGGAGTTTCCAAGAGATAAGCTGACACTGGGCAAGCCCCTGGGAGAAGGTTGCTTTGGGCAAGTGGTCATGGCGGAAGCAGTGGGAATTGACAAAGACAAGCCCAAGGAGGCGGTCACCGTGGCCGTGAAGATGTTGAAAGATGATGCCACAGAGAAAGACCTTTCTGATCTGGTGTCAGAGATGGAGATGATGAAGATGATTGGGAAACACAAGAATATCATAAATCTTCTTGGAGCCTGCACACAGGATGGGCCTCTCTATGTCATAGTTGAGTATGCCTCTAAAGGCAACCTCCGAGAATACCTCCGAGCCCGGAGGCCACCCGGGATGGAGTACTCCTATGACATTAACCGTGTTCCTGAGGAGCAGATGACCTTCAAGGACTTGGTGTCATGCACCTACCAGCTGGCCAGAGGCATGGAGTACTTGGCTTCCCAAAAATGTATTCATCGAGATTTAGCAGCCAGAAATGTTTTGGTAACAGAAAACAATGTGATGAAAATAGCAGACTTTGGACTCGCCAGAGATATCAACAATATAGACTATTACAAAAAGACCACCAATGGGCGGCTTCCAGTCAAGTGGATGGCTCCAGAAGCCCTGTTTGATAGAGTATACACTCATCAGAGTGATGTCTGGTCCTTCGGGGTGTTAATGTGGGAGATCTTCACTTTAGGGGGCTCGCCCTACCCAGGGATTCCCGTGGAGGAACTTTTTAAGCTGCTGAAGGAAGGACACAGAATGGATAAGCCAGCCAACTGCACCAACGAACTGTACATGATGATGAGGGACTGTTGGCATGCAGTGCCCTCCCAGAGACCAACGTTCAAGCAGTTGGTAGAAGACTTGGATCGAATTCTCACTCTCACAACCAATGAGGAATACTTGGACCTCAGCCAACCTCTCGAACAGTATTCACCTAGTTACCCTGACACAAGAAGTTCTTGTTCTTCAGGAGATGATTCTGTTTTTTCTCCAGACCCCATGCCTTACGAACCATGCCTTCCTCAGTATCCACACATAAACGGCAGTGTTAAAACAAAGGGTCAAGACAATTCTGCAGATATCCAGCACAGTGGCGGCCGCTCGAGTCTAGAGGGCCCGCGGTTCGAAGGTAAGCCTATCCCTAACCCTCTCCTCGGTCTCGATTCTACGCGTACCGGTCATCATCACCATCACCATTGA

Receptor sequence

V5 tag

6x His

pcDNA3.1-FGFR2 M391R:

TCTCTGGCTAACTAGAGAACCCACTGCTTACTGGCTTATCGAAATTAATACGACTCACTATAGGGAGACCCAAGCTGGCTAGTTAAGCTTGGTACCGAGCTCGGATCCAGTACCCTTCACCATGGTCAGCTGGGGTCGTTTCATCTGCCTGGTCGTGGTCACCATGGCAACCTTGTCCCTGGCCCGGCCCTCCTTCAGTTTAGTTGAGGATACCACATTAGAGCCAGAAGAGCCACCAACCAAATACCAAATCTCTCAACCAGAAGTGTACGTGGCTGCGCCAGGGGAGTCGCTAGAGGTGCGCTGCCTGTTGAAAGATGCCGCCGTGATCAGTTGGACTAAGGATGGGGTGCACTTGGGGCCCAACAATAGGACAGTGCTTATTGGGGAGTACTTGCAGATAAAGGGCGCCACGCCTAGAGACTCCGGCCTCTATGCTTGTACTGCCAGTAGGACTGTAGACAGTGAAACTTGGTACTTCATGGTGAATGTCACAGATGCCATCTCATCCGGAGATGATGAGGATGACACCGATGGTGCGGAAGATTTTGTCAGTGAGAACAGTAACAACAAGAGAGCACCATACTGGACCAACACAGAAAAGATGGAAAAGCGGCTCCATGCTGTGCCTGCGGCCAACACTGTCAAGTTTCGCTGCCCAGCCGGGGGGAACCCAATGCCAACCATGCGGTGGCTGAAAAACGGGAAGGAGTTTAAGCAGGAGCATCGCATTGGAGGCTACAAGGTACGAAACCAGCACTGGAGCCTCATTATGGAAAGTGTGGTCCCATCTGACAAGGGAAATTATACCTGTGTAGTGGAGAATGAATACGGGTCCATCAATCACACGTACCACCTGGATGTTGTGGAGCGATCGCCTCACCGGCCCATCCTCCAAGCCGGACTGCCGGCAAATGCCTCCACAGTGGTCGGAGGAGACGTAGAGTTTGTCTGCAAGGTTTACAGTGATGCCCAGCCCCACATCCAGTGGATCAAGCACGTGGAAAAGAACGGCAGTAAATACGGGCCCGACGGGCTGCCCTACCTCAAGGTTCTCAAGGCCGCCGGTGTTAACACCACGGACAAAGAGATTGAGGTTCTCTATATTCGGAATGTAACTTTTGAGGACGCTGGGGAATATACGTGCTTGGCGGGTAATTCTATTGGGATATCCTTTCACTCTGCATGGTTGACAGTTCTGCCAGCGCCTGGAAGAGAAAAGGAGATTACAGCTTCCCCAGACTACCTGGAGATAGCCATTTACTGCATAGGGGTCTTCTTAATCGCCTGTAGGGTGGTAACAGTCATCCTGTGCCGAATGAAGAACACGACCAAGAAGCCAGACTTCAGCAGCCAGCCGGCTGTGCACAAGCTGACCAAACGTATCCCCCTGCGGAGACAGGTAACAGTTTCGGCTGAGTCCAGCTCCTCCATGAACTCCAACACCCCGCTGGTGAGGATAACAACACGCCTCTCTTCAACGGCAGACACCCCCATGCTGGCAGGGGTCTCCGAGTATGAACTTCCAGAGGACCCAAAATGGGAGTTTCCAAGAGATAAGCTGACACTGGGCAAGCCCCTGGGAGAAGGTTGCTTTGGGCAAGTGGTCATGGCGGAAGCAGTGGGAATTGACAAAGACAAGCCCAAGGAGGCGGTCACCGTGGCCGTGAAGATGTTGAAAGATGATGCCACAGAGAAAGACCTTTCTGATCTGGTGTCAGAGATGGAGATGATGAAGATGATTGGGAAACACAAGAATATCATAAATCTTCTTGGAGCCTGCACACAGGATGGGCCTCTCTATGTCATAGTTGAGTATGCCTCTAAAGGCAACCTCCGAGAATACCTCCGAGCCCGGAGGCCACCCGGGATGGAGTACTCCTATGACATTAACCGTGTTCCTGAGGAGCAGATGACCTTCAAGGACTTGGTGTCATGCACCTACCAGCTGGCCAGAGGCATGGAGTACTTGGCTTCCCAAAAATGTATTCATCGAGATTTAGCAGCCAGAAATGTTTTGGTAACAGAAAACAATGTGATGAAAATAGCAGACTTTGGACTCGCCAGAGATATCAACAATATAGACTATTACAAAAAGACCACCAATGGGCGGCTTCCAGTCAAGTGGATGGCTCCAGAAGCCCTGTTTGATAGAGTATACACTCATCAGAGTGATGTCTGGTCCTTCGGGGTGTTAATGTGGGAGATCTTCACTTTAGGGGGCTCGCCCTACCCAGGGATTCCCGTGGAGGAACTTTTTAAGCTGCTGAAGGAAGGACACAGAATGGATAAGCCAGCCAACTGCACCAACGAACTGTACATGATGATGAGGGACTGTTGGCATGCAGTGCCCTCCCAGAGACCAACGTTCAAGCAGTTGGTAGAAGACTTGGATCGAATTCTCACTCTCACAACCAATGAGGAATACTTGGACCTCAGCCAACCTCTCGAACAGTATTCACCTAGTTACCCTGACACAAGAAGTTCTTGTTCTTCAGGAGATGATTCTGTTTTTTCTCCAGACCCCATGCCTTACGAACCATGCCTTCCTCAGTATCCACACATAAACGGCAGTGTTAAAACAAAGGGTCAAGACAATTCTGCAGATATCCAGCACAGTGGCGGCCGCTCGAGTCTAGAGGGCCCGCGGTTCGAAGGTAAGCCTATCCCTAACCCTCTCCTCGGTCTCGATTCTACGCGTACCGGTCATCATCACCATCACCATTGA

Receptor sequence

V5 tag

6x His

pcDNA3.1-FGFR2-ΔL2;R450_L451insL2:

TCTCTGGCTAACTAGAGAACCCACTGCTTACTGGCTTATCGAAATTAATACGACTCACTATAGGGAGACCCAAGCTGGCTAGTTAAGCTTGGTACCGAGCTCGGATCCAGTACCCTTCACCATGGTCAGCTGGGGTCGTTTCATCTGCCTGGTCGTGGTCACCATGGCAACCTTGTCCCTGGCCCGGCCCTCCTTCAGTTTAGTTGAGGATACCACATTAGAGCCAGAAGAGCCACCAACCAAATACCAAATCTCTCAACCAGAAGTGTACGTGGCTGCGCCAGGGGAGTCGCTAGAGGTGCGCTGCCTGTTGAAAGATGCCGCCGTGATCAGTTGGACTAAGGATGGGGTGCACTTGGGGCCCAACAATAGGACAGTGCTTATTGGGGAGTACTTGCAGATAAAGGGCGCCACGCCTAGAGACTCCGGCCTCTATGCTTGTACTGCCAGTAGGACTGTAGACAGTGAAACTTGGTACTTCATGGTGAATGTCACAGATGCCATCTCATCCGGAGATGATGAGGATGACACCGATGGTGCGGAAGATTTTGTCAGTGAGAACAGTAACAACAAGAGAGCACCATACTGGACCAACACAGAAAAGATGGAAAAGCGGCTCCATGCTGTGCCTGCGGCCAACACTGTCAAGTTTCGCTGCCCAGCCGGGGGGAACCCAATGCCAACCATGCGGTGGCTGAAAAACGGGAAGGAGTTTAAGCAGGAGCATCGCATTGGAGGCTACAAGGTACGAAACCAGCACTGGAGCCTCATTATGGAAAGTGTGGTCCCATCTGACAAGGGAAATTATACCTGTGTAGTGGAGAATGAATACGGGTCCATCAATCACACGTACCACCTGGATGTTGTGGAGCGATCGCCTCACCGGCCCATCCTCCAAGCCGGACTGCCGGCAAATGCCTCCACAGTGGTCGGAGGAGACGTAGAGTTTGTCTGCAAGGTTTACAGTGATGCCCAGCCCCACATCCAGTGGATCAAGCACGTGGAAAAGAACGGCAGTAAATACGGGCCCGACGGGCTGCCCTACCTCAAGGTTCTCAAGGCCGCCGGTGTTAACACCACGGACAAAGAGATTGAGGTTCTCTATATTCGGAATGTAACTTTTGAGGACGCTGGGGAATATACGTGCTTGGCGGGTAATTCTATTGGGATATCCTTTCACTCTGCATGGTTGACAGTTCTGCCAGCGCCTGGAAGAGAAAAGGAGATTACAGCTTCCCCAGACTACCTGGAGATAGCCATTTACTGCATAGGGGTCTTCTTAATCGCCTGTATGGTGGTAACAGTCATCCTGTGCCGAATGAAGAACACGACCAAGAAGCCAGACTTCAGCAGCCAGCCGGCTGTGCACAAGCTGACCAAACGTATCCCCCTGCGGAGACAGTCCAGCTCCTCCATGAACTCCAACACCCCGCTGGTGAGGATAACAACACGCGTAACAGTTTCGGCTGAGCTCTCTTCAACGGCAGACACCCCCATGCTGGCAGGGGTCTCCGAGTATGAACTTCCAGAGGACCCAAAATGGGAGTTTCCAAGAGATAAGCTGACACTGGGCAAGCCCCTGGGAGAAGGTTGCTTTGGGCAAGTGGTCATGGCGGAAGCAGTGGGAATTGACAAAGACAAGCCCAAGGAGGCGGTCACCGTGGCCGTGAAGATGTTGAAAGATGATGCCACAGAGAAAGACCTTTCTGATCTGGTGTCAGAGATGGAGATGATGAAGATGATTGGGAAACACAAGAATATCATAAATCTTCTTGGAGCCTGCACACAGGATGGGCCTCTCTATGTCATAGTTGAGTATGCCTCTAAAGGCAACCTCCGAGAATACCTCCGAGCCCGGAGGCCACCCGGGATGGAGTACTCCTATGACATTAACCGTGTTCCTGAGGAGCAGATGACCTTCAAGGACTTGGTGTCATGCACCTACCAGCTGGCCAGAGGCATGGAGTACTTGGCTTCCCAAAAATGTATTCATCGAGATTTAGCAGCCAGAAATGTTTTGGTAACAGAAAACAATGTGATGAAAATAGCAGACTTTGGACTCGCCAGAGATATCAACAATATAGACTATTACAAAAAGACCACCAATGGGCGGCTTCCAGTCAAGTGGATGGCTCCAGAAGCCCTGTTTGATAGAGTATACACTCATCAGAGTGATGTCTGGTCCTTCGGGGTGTTAATGTGGGAGATCTTCACTTTAGGGGGCTCGCCCTACCCAGGGATTCCCGTGGAGGAACTTTTTAAGCTGCTGAAGGAAGGACACAGAATGGATAAGCCAGCCAACTGCACCAACGAACTGTACATGATGATGAGGGACTGTTGGCATGCAGTGCCCTCCCAGAGACCAACGTTCAAGCAGTTGGTAGAAGACTTGGATCGAATTCTCACTCTCACAACCAATGAGGAATACTTGGACCTCAGCCAACCTCTCGAACAGTATTCACCTAGTTACCCTGACACAAGAAGTTCTTGTTCTTCAGGAGATGATTCTGTTTTTTCTCCAGACCCCATGCCTTACGAACCATGCCTTCCTCAGTATCCACACATAAACGGCAGTGTTAAAACAGCCAAGGGTCAAGACAATTCTGCAGATATCCAGCACAGTGGCGGCCGCTCGAGTCTAGAGGGCCCGCGGTTCGAAGGTAAGCCTATCCCTAACCCTCTCCTCGGTCTCGATTCTACGCGTACCGGTCATCATCACCATCACCATTGA

Receptor sequence

V5 tag

6x His

pDonor MCS Rosa26-CMV-FGFR2IIIb-V5-delTV-Puro

ACGCCCACACACCAGGTTAGCCTTTAAGCCTGCCCAGAAGACTCCCGCCCATCTGCGGCCGCGTCGATAATTTGAATAGATATTAAGTTTTATTATATTTACACTTACATACTAATAATAAATTCAACAAACAATTTATTTATGTTTATTTATTTATTAAAAAAAAACAAAAACTCAAAATTTCTTCTATAAAGTAACAAAACTTTTATGAGGGACAGCCCCCCCCCAAAGCCCCCAGGGATGTAATTACGTCCCTCCCCCGCTAGGGGGCAGCAGCGAGCCGCCCGGGGCTCCGCTCCGGTCCGGCGCTCCCCCCGCATCCCCGAGCCGGCAGCGTGCGGGGACAGCCCGGGCACGGGGAAGGTGGCACGGGATCGCTTTCCTCTGAACGCTTCTCGCTGCTCTTTGAGCCTGCAGACACCTGGGGGGATACGGGGAAAAGGCCTCCACGGCCACTAGTGCTAGTATGCCAAGTACGCCCCCTATTGACGTCAATGACGGTAAATGGCCCGCCTGGCATTATGCCCAGTACATGACCTTATGGGACTTTCCTACTTGGCAGTACATCTACGTATTAGTCATCGCTATTACCATGGTGATGCGGTTTTGGCAGTACATCAATGGGCGTGGATAGCGGTTTGACTCACGGGGATTTCCAAGTCTCCACCCCATTGACGTCAATGGGAGTTTGTTTTGGCACCAAAATCAACGGGACTTTCCAAAATGTCGTAACAACTCCGCCCCATTGACGCAAATGGGCGGTAGGCGTGTACGGTGGGAGGTCTATATAAGCAGAGCTCTCTGGCTAACTAGAGAACCCACTGCTTACTGGCTTATCGAAATTAATACGACTCACTATAGGGAGACCCAAGCTGGCTAGTTAAGCTTGGTACCGAGCTCGGATCCAGTACCCTTCACCATGGTCAGCTGGGGTCGTTTCATCTGCCTGGTCGTGGTCACCATGGCAACCTTGTCCCTGGCCCGGCCCTCCTTCAGTTTAGTTGAGGATACCACATTAGAGCCAGAAGAGCCACCAACCAAATACCAAATCTCTCAACCAGAAGTGTACGTGGCTGCGCCAGGGGAGTCGCTAGAGGTGCGCTGCCTGTTGAAAGATGCCGCCGTGATCAGTTGGACTAAGGATGGGGTGCACTTGGGGCCCAACAATAGGACAGTGCTTATTGGGGAGTACTTGCAGATAAAGGGCGCCACGCCTAGAGACTCCGGCCTCTATGCTTGTACTGCCAGTAGGACTGTAGACAGTGAAACTTGGTACTTCATGGTGAATGTCACAGATGCCATCTCATCCGGAGATGATGAGGATGACACCGATGGTGCGGAAGATTTTGTCAGTGAGAACAGTAACAACAAGAGAGCACCATACTGGACCAACACAGAAAAGATGGAAAAGCGGCTCCATGCTGTGCCTGCGGCCAACACTGTCAAGTTTCGCTGCCCAGCCGGGGGAAACCCAATGCCAACCATGCGGTGGCTGAAAAACGGGAAGGAGTTTAAGCAGGAGCATCGCATTGGAGGCTACAAGGTACGAAACCAGCACTGGAGCCTCATTATGGAAAGTGTGGTCCCATCTGACAAGGGAAATTATACCTGTGTGGTGGAGAATGAATACGGGTCCATCAATCACACGTACCACCTGGATGTTGTGGAGCGATCGCCTCACCGGCCCATCCTCCAAGCCGGACTGCCGGCAAATGCCTCCACAGTGGTCGGAGGAGACGTAGAGTTTGTCTGCAAGGTTTACAGTGATGCCCAGCCCCACATCCAGTGGATCAAGCACGTGGAAAAGAACGGCAGTAAATACGGGCCCGACGGGCTGCCCTACCTCAAGGTTCTCAAGCACTCGGGGATAAATAGTTCCAATGCAGAAGTGCTGGCTCTGTTCAATGTGACCGAGGCGGATGCTGGGGAATATATATGTAAGGTCTCCAATTATATAGGGCAGGCCAACCAGTCTGCCTGGCTCACTGTCCTGCCAAAACAGCAAGCGCCTGGAAGAGAAAAGGAGATTACAGCTTCCCCAGACTACCTGGAGATAGCCATTTACTGCATAGGGGTCTTCTTAATCGCCTGTATGGTGGTAACAGTCATCCTGTGCCGAATGAAGAACACGACCAAGAAGCCAGACTTCAGCAGCCAGCCGGCTGTGCACAAGCTGACCAAACGTATCCCCCTGCGGAGACAGGTATCGGCTGAGTCCAGCTCCTCCATGAACTCCAACACCCCGCTGGTGAGGATAACAACACGCCTCTCTTCAACGGCAGACACCCCCATGCTGGCAGGGGTCTCCGAGTATGAACTTCCAGAGGACCCAAAATGGGAGTTTCCAAGAGATAAGCTGACACTGGGCAAGCCCCTGGGAGAAGGTTGCTTTGGGCAAGTGGTCATGGCGGAAGCAGTGGGAATTGACAAAGACAAGCCCAAGGAGGCGGTCACCGTGGCCGTGAAGATGTTGAAAGATGATGCCACAGAGAAAGACCTTTCTGATCTGGTGTCAGAGATGGAGATGATGAAGATGATTGGGAAACACAAGAATATCATAAATCTTCTTGGAGCCTGCACACAGGATGGGCCTCTCTATGTCATAGTTGAGTATGCCTCTAAAGGCAACCTCCGAGAATACCTCCGAGCCCGGAGGCCACCCGGGATGGAGTACTCCTATGACATTAACCGTGTTCCTGAGGAGCAGATGACCTTCAAGGACTTGGTGTCATGCACCTACCAGCTGGCCAGAGGCATGGAGTACTTGGCTTCCCAAAAATGTATTCATCGAGATTTAGCAGCCAGAAATGTTTTGGTAACAGAAAACAATGTGATGAAAATAGCAGACTTTGGACTCGCCAGAGATATCAACAATATAGACTATTACAAAAAGACCACCAATGGGCGGCTTCCAGTCAAGTGGATGGCTCCAGAAGCCCTGTTTGATAGAGTATACACTCATCAGAGTGATGTCTGGTCCTTCGGGGTGTTAATGTGGGAGATCTTCACTTTAGGGGGCTCGCCCTACCCAGGGATTCCCGTGGAGGAACTTTTTAAGCTGCTGAAGGAAGGACACAGAATGGATAAGCCAGCCAACTGCACCAACGAACTGTACATGATGATGAGGGACTGTTGGCATGCAGTGCCCTCCCAGAGACCAACGTTCAAGCAGTTGGTAGAAGACTTGGATCGAATCCTCACTCTCACAACCAATGAGGAATACTTGGACCTCAGCCAACCTCTCGAACAGTATTCACCTAGTTACCCTGACACAAGAAGTTCTTGTTCTTCAGGAGATGATTCTGTTTTTTCTCCAGACCCCATGCCTTACGAACCATGCCTTCCTCAGTATCCACACATAAACGGCAGTGTTAAAACAGCGAAGGGTCAAGACAATTCTGCAGATATCCAGCACAGTGGCGGCCGCTCGAGTCTAGAGGGCCCGCGGTTCGAAGGTAAGCCTATCCCTAACCCTCTCCTCGGTCTCGATTCTACGCGTACCGGTCATCATCACCATCACCATTGAGTTTAGTCGGGGCGGCCGGCCGCTTCGAGCAGACATGATAAGATACATTGATGAGTTTGGACAAACCACAACTAGAATGCAGTGAAAAAAATGCTTTATTTGTGAAATTTGTGATGCTATTGCTTTATTTGTAACCATTATAAGCTGCAATAAACAAGTTAACAACAACAATTGCATTCATTTTATGTTTCAGGTTCAGGGGGAGGTGTGGGAGGTTTTTTAAAGCAAGTAAAACCTCTACAAATGTGGTAAAATCGATAAGGATCCGTTTGCGTATTGGGCGCTCTTCCGCTGATCTGCGCAGCACCATGGCCTGAAATAACCTCTGAAAGAGGAACTTGGTTAGCTACCTTCTAAATCGGATCCGCGGCCGCAAGGATCTGCGATCGCTCCGGTGCCCGTCAGTGGGCAGAGCGCACATCGCCCACAGTCCCCGAGAAGTTGGGGGGAGGGGTCGGCAATTGAACGGGTGCCTAGAGAAGGTGGCGCGGGGTAAACTGGGAAAGTGATGTCGTGTACTGGCTCCGCCTTTTTCCCGAGGGTGGGGGAGAACCGTATATAAGTGCAGTAGTCGCCGTGAACGTTCTTTTTCGCAACGGGTTTGCCGCCAGAACACAGCTGAAGCTTCGAGGGGCTCGCATCTCTCCTTCACGCGCCCGCCGCCCTACCTGAGGCCGCCATCCACGCCGGTTGAGTCGCGTTCTGCCGCCTCCCGCCTGTGGTGCCTCCTGAACTGCGTCCGCCGTCTAGGTAAGTTTAAAGCTCAGGTCGAGACCGGGCCTTTGTCCGGCGCTCCCTTGGAGCCTACCTAGACTCAGCCGGCTCTCCACGCTTTGCCTGACCCTGCTTGCTCAACTCTACGTCTTTGTTTCGTTTTCTGTTCTGCGCCGTTACAGATCCAAGCTGTGACCGGCGCCTACGCTAGACGCCATCCATGGCCGAGTACAAGCCCACGGTGCGCCTCGCCACCCGCGACGACGTCCCCAGGGCCGTACGCACCCTCGCCGCCGCGTTCGCCGACTACCCCGCCACGCGCCACACCGTCGATCCGGACCGCCACATCGAGCGGGTCACCGAGCTGCAAGAACTCTTCCTCACGCGCGTCGGGCTCGACATCGGCAAGGTGTGGGTCGCGGACGACGGCGCCGCGGTGGCGGTCTGGACCACGCCGGAGAGCGTCGAAGCGGGGGCGGTGTTCGCCGAGATCGGCCCGCGCATGGCCGAGTTGAGCGGTTCCCGGCTGGCCGCGCAGCAACAGATGGAAGGCCTCCTGGCGCCGCACCGGCCCAAGGAGCCCGCGTGGTTCCTGGCCACCGTCGGCGTCTCGCCCGACCACCAGGGCAAGGGTCTGGGCAGCGCCGTCGTGCTCCCCGGAGTGGAGGCGGCCGAGCGCGCCGGGGTGCCCGCCTTCCTGGAGACCTCCGCGCCCCGCAACCTCCCCTTCTACGAGCGGCTCGGCTTCACCGTCACCGCCGACGTCGAGGTGCCCGAAGGACCGCGCACCTGGTGCATGACCCGCAAGCCCGGTGCCTGAAATCAACCTCTGGATTACAAAATTTGTGAAAGATTGACTGAAACCCGCTGATCAGCCTCGACTGTGCCTTCTAGTTGCCAGCCATCTGTTGTTTGCCCCTCCCCCGTGCCTTCCTTGACCCTGGAAGGTGCCACTCCCACTGTCCTTTCCTAATAAAATGAGGAAATTGCATCGCATTGTCTGAGTAGGTGTCATTCTATTCTGGGGGGTGGGGTGGGGCAGGACAGCAAGGGGGAGGATTGGGAAGACAATAGCAGGCATGCTGGGGATGCGGTGGGCTCTATGGCTTCTGAGGCGGAAAGAACCAGCTGGGGCTCTAGGGGGTATCCCCACGCGCCCTGTAGCGGCGCATTAAGCGCGGCGGGTGTGGTGGTTACGCGCAGCGTGACCGCTACACTTGCCAGCGCCCTAGCGCCCGCTCCTTTCGCTTTCTTCCCTTCCTTTCTCGCCACGTTCGCCGGCTTTCCCCGTCAAGCTCTAAATCGGGGGCTCCCTTTAGGGTTCCGATTTAGTGCTTTACGGCACCTCGACCCCAAAAAACTTGATTAGGGTGATGGTTCACGTAGTGGGCCATTGGTATGGCTTTTTCCCCGTATCCCCCCAGGTGTCTGCAGGCTCAAAGAGCAGCGAGAAGCGTTCAGAGGAAAGCGATCCCGTGCCACCTTCCCCGTGCCCGGGCTGTCCCCGCACGCTGCCGGCTCGGGGATGCGGGGGGAGCGCCGGACCGGAGCGGAGCCCCGGGCGGCTCGCTGCTGCCCCCTAGCGGGGGAGGGACGTAATTACATCCCTGGGGGCTTTGGGGGGGGGCTGTCCCTGATAATTCTCTAGAAAGACTGGAGTTGCAGATCACGAGGGAAG

Receptor sequence

V5 tag

6x His

Puromycin resistance

Insulator sequence

pDonor MCS Rosa26-CMV-FGFR2IIIb-V5-WT-Puro

ACGCCCACACACCAGGTTAGCCTTTAAGCCTGCCCAGAAGACTCCCGCCCATCTGCGGCCGCGTCGATAATTTGAATAGATATTAAGTTTTATTATATTTACACTTACATACTAATAATAAATTCAACAAACAATTTATTTATGTTTATTTATTTATTAAAAAAAAACAAAAACTCAAAATTTCTTCTATAAAGTAACAAAACTTTTATGAGGGACAGCCCCCCCCCAAAGCCCCCAGGGATGTAATTACGTCCCTCCCCCGCTAGGGGGCAGCAGCGAGCCGCCCGGGGCTCCGCTCCGGTCCGGCGCTCCCCCCGCATCCCCGAGCCGGCAGCGTGCGGGGACAGCCCGGGCACGGGGAAGGTGGCACGGGATCGCTTTCCTCTGAACGCTTCTCGCTGCTCTTTGAGCCTGCAGACACCTGGGGGGATACGGGGAAAAGGCCTCCACGGCCACTAGTGCTAGTATGCCAAGTACGCCCCCTATTGACGTCAATGACGGTAAATGGCCCGCCTGGCATTATGCCCAGTACATGACCTTATGGGACTTTCCTACTTGGCAGTACATCTACGTATTAGTCATCGCTATTACCATGGTGATGCGGTTTTGGCAGTACATCAATGGGCGTGGATAGCGGTTTGACTCACGGGGATTTCCAAGTCTCCACCCCATTGACGTCAATGGGAGTTTGTTTTGGCACCAAAATCAACGGGACTTTCCAAAATGTCGTAACAACTCCGCCCCATTGACGCAAATGGGCGGTAGGCGTGTACGGTGGGAGGTCTATATAAGCAGAGCTCTCTGGCTAACTAGAGAACCCACTGCTTACTGGCTTATCGAAATTAATACGACTCACTATAGGGAGACCCAAGCTGGCTAGTTAAGCTTGGTACCGAGCTCGGATCCAGTACCCTTCACCATGGTCAGCTGGGGTCGTTTCATCTGCCTGGTCGTGGTCACCATGGCAACCTTGTCCCTGGCCCGGCCCTCCTTCAGTTTAGTTGAGGATACCACATTAGAGCCAGAAGAGCCACCAACCAAATACCAAATCTCTCAACCAGAAGTGTACGTGGCTGCGCCAGGGGAGTCGCTAGAGGTGCGCTGCCTGTTGAAAGATGCCGCCGTGATCAGTTGGACTAAGGATGGGGTGCACTTGGGGCCCAACAATAGGACAGTGCTTATTGGGGAGTACTTGCAGATAAAGGGCGCCACGCCTAGAGACTCCGGCCTCTATGCTTGTACTGCCAGTAGGACTGTAGACAGTGAAACTTGGTACTTCATGGTGAATGTCACAGATGCCATCTCATCCGGAGATGATGAGGATGACACCGATGGTGCGGAAGATTTTGTCAGTGAGAACAGTAACAACAAGAGAGCACCATACTGGACCAACACAGAAAAGATGGAAAAGCGGCTCCATGCTGTGCCTGCGGCCAACACTGTCAAGTTTCGCTGCCCAGCCGGGGGAAACCCAATGCCAACCATGCGGTGGCTGAAAAACGGGAAGGAGTTTAAGCAGGAGCATCGCATTGGAGGCTACAAGGTACGAAACCAGCACTGGAGCCTCATTATGGAAAGTGTGGTCCCATCTGACAAGGGAAATTATACCTGTGTGGTGGAGAATGAATACGGGTCCATCAATCACACGTACCACCTGGATGTTGTGGAGCGATCGCCTCACCGGCCCATCCTCCAAGCCGGACTGCCGGCAAATGCCTCCACAGTGGTCGGAGGAGACGTAGAGTTTGTCTGCAAGGTTTACAGTGATGCCCAGCCCCACATCCAGTGGATCAAGCACGTGGAAAAGAACGGCAGTAAATACGGGCCCGACGGGCTGCCCTACCTCAAGGTTCTCAAGCACTCGGGGATAAATAGTTCCAATGCAGAAGTGCTGGCTCTGTTCAATGTGACCGAGGCGGATGCTGGGGAATATATATGTAAGGTCTCCAATTATATAGGGCAGGCCAACCAGTCTGCCTGGCTCACTGTCCTGCCAAAACAGCAAGCGCCTGGAAGAGAAAAGGAGATTACAGCTTCCCCAGACTACCTGGAGATAGCCATTTACTGCATAGGGGTCTTCTTAATCGCCTGTATGGTGGTAACAGTCATCCTGTGCCGAATGAAGAACACGACCAAGAAGCCAGACTTCAGCAGCCAGCCGGCTGTGCACAAGCTGACCAAACGTATCCCCCTGCGGAGACAGGTAACAGTTTCGGCTGAGTCCAGCTCCTCCATGAACTCCAACACCCCGCTGGTGAGGATAACAACACGCCTCTCTTCAACGGCAGACACCCCCATGCTGGCAGGGGTCTCCGAGTATGAACTTCCAGAGGACCCAAAATGGGAGTTTCCAAGAGATAAGCTGACACTGGGCAAGCCCCTGGGAGAAGGTTGCTTTGGGCAAGTGGTCATGGCGGAAGCAGTGGGAATTGACAAAGACAAGCCCAAGGAGGCGGTCACCGTGGCCGTGAAGATGTTGAAAGATGATGCCACAGAGAAAGACCTTTCTGATCTGGTGTCAGAGATGGAGATGATGAAGATGATTGGGAAACACAAGAATATCATAAATCTTCTTGGAGCCTGCACACAGGATGGGCCTCTCTATGTCATAGTTGAGTATGCCTCTAAAGGCAACCTCCGAGAATACCTCCGAGCCCGGAGGCCACCCGGGATGGAGTACTCCTATGACATTAACCGTGTTCCTGAGGAGCAGATGACCTTCAAGGACTTGGTGTCATGCACCTACCAGCTGGCCAGAGGCATGGAGTACTTGGCTTCCCAAAAATGTATTCATCGAGATTTAGCAGCCAGAAATGTTTTGGTAACAGAAAACAATGTGATGAAAATAGCAGACTTTGGACTCGCCAGAGATATCAACAATATAGACTATTACAAAAAGACCACCAATGGGCGGCTTCCAGTCAAGTGGATGGCTCCAGAAGCCCTGTTTGATAGAGTATACACTCATCAGAGTGATGTCTGGTCCTTCGGGGTGTTAATGTGGGAGATCTTCACTTTAGGGGGCTCGCCCTACCCAGGGATTCCCGTGGAGGAACTTTTTAAGCTGCTGAAGGAAGGACACAGAATGGATAAGCCAGCCAACTGCACCAACGAACTGTACATGATGATGAGGGACTGTTGGCATGCAGTGCCCTCCCAGAGACCAACGTTCAAGCAGTTGGTAGAAGACTTGGATCGAATCCTCACTCTCACAACCAATGAGGAATACTTGGACCTCAGCCAACCTCTCGAACAGTATTCACCTAGTTACCCTGACACAAGAAGTTCTTGTTCTTCAGGAGATGATTCTGTTTTTTCTCCAGACCCCATGCCTTACGAACCATGCCTTCCTCAGTATCCACACATAAACGGCAGTGTTAAAACAGCGAAGGGTCAAGACAATTCTGCAGATATCCAGCACAGTGGCGGCCGCTCGAGTCTAGAGGGCCCGCGGTTCGAAGGTAAGCCTATCCCTAACCCTCTCCTCGGTCTCGATTCTACGCGTACCGGTCATCATCACCATCACCATTGAGTTTAGTCGGGGCGGCCGGCCGCTTCGAGCAGACATGATAAGATACATTGATGAGTTTGGACAAACCACAACTAGAATGCAGTGAAAAAAATGCTTTATTTGTGAAATTTGTGATGCTATTGCTTTATTTGTAACCATTATAAGCTGCAATAAACAAGTTAACAACAACAATTGCATTCATTTTATGTTTCAGGTTCAGGGGGAGGTGTGGGAGGTTTTTTAAAGCAAGTAAAACCTCTACAAATGTGGTAAAATCGATAAGGATCCGTTTGCGTATTGGGCGCTCTTCCGCTGATCTGCGCAGCACCATGGCCTGAAATAACCTCTGAAAGAGGAACTTGGTTAGCTACCTTCTAAATCGGATCCGCGGCCGCAAGGATCTGCGATCGCTCCGGTGCCCGTCAGTGGGCAGAGCGCACATCGCCCACAGTCCCCGAGAAGTTGGGGGGAGGGGTCGGCAATTGAACGGGTGCCTAGAGAAGGTGGCGCGGGGTAAACTGGGAAAGTGATGTCGTGTACTGGCTCCGCCTTTTTCCCGAGGGTGGGGGAGAACCGTATATAAGTGCAGTAGTCGCCGTGAACGTTCTTTTTCGCAACGGGTTTGCCGCCAGAACACAGCTGAAGCTTCGAGGGGCTCGCATCTCTCCTTCACGCGCCCGCCGCCCTACCTGAGGCCGCCATCCACGCCGGTTGAGTCGCGTTCTGCCGCCTCCCGCCTGTGGTGCCTCCTGAACTGCGTCCGCCGTCTAGGTAAGTTTAAAGCTCAGGTCGAGACCGGGCCTTTGTCCGGCGCTCCCTTGGAGCCTACCTAGACTCAGCCGGCTCTCCACGCTTTGCCTGACCCTGCTTGCTCAACTCTACGTCTTTGTTTCGTTTTCTGTTCTGCGCCGTTACAGATCCAAGCTGTGACCGGCGCCTACGCTAGACGCCATCCATGGCCGAGTACAAGCCCACGGTGCGCCTCGCCACCCGCGACGACGTCCCCAGGGCCGTACGCACCCTCGCCGCCGCGTTCGCCGACTACCCCGCCACGCGCCACACCGTCGATCCGGACCGCCACATCGAGCGGGTCACCGAGCTGCAAGAACTCTTCCTCACGCGCGTCGGGCTCGACATCGGCAAGGTGTGGGTCGCGGACGACGGCGCCGCGGTGGCGGTCTGGACCACGCCGGAGAGCGTCGAAGCGGGGGCGGTGTTCGCCGAGATCGGCCCGCGCATGGCCGAGTTGAGCGGTTCCCGGCTGGCCGCGCAGCAACAGATGGAAGGCCTCCTGGCGCCGCACCGGCCCAAGGAGCCCGCGTGGTTCCTGGCCACCGTCGGCGTCTCGCCCGACCACCAGGGCAAGGGTCTGGGCAGCGCCGTCGTGCTCCCCGGAGTGGAGGCGGCCGAGCGCGCCGGGGTGCCCGCCTTCCTGGAGACCTCCGCGCCCCGCAACCTCCCCTTCTACGAGCGGCTCGGCTTCACCGTCACCGCCGACGTCGAGGTGCCCGAAGGACCGCGCACCTGGTGCATGACCCGCAAGCCCGGTGCCTGAAATCAACCTCTGGATTACAAAATTTGTGAAAGATTGACTGAAACCCGCTGATCAGCCTCGACTGTGCCTTCTAGTTGCCAGCCATCTGTTGTTTGCCCCTCCCCCGTGCCTTCCTTGACCCTGGAAGGTGCCACTCCCACTGTCCTTTCCTAATAAAATGAGGAAATTGCATCGCATTGTCTGAGTAGGTGTCATTCTATTCTGGGGGGTGGGGTGGGGCAGGACAGCAAGGGGGAGGATTGGGAAGACAATAGCAGGCATGCTGGGGATGCGGTGGGCTCTATGGCTTCTGAGGCGGAAAGAACCAGCTGGGGCTCTAGGGGGTATCCCCACGCGCCCTGTAGCGGCGCATTAAGCGCGGCGGGTGTGGTGGTTACGCGCAGCGTGACCGCTACACTTGCCAGCGCCCTAGCGCCCGCTCCTTTCGCTTTCTTCCCTTCCTTTCTCGCCACGTTCGCCGGCTTTCCCCGTCAAGCTCTAAATCGGGGGCTCCCTTTAGGGTTCCGATTTAGTGCTTTACGGCACCTCGACCCCAAAAAACTTGATTAGGGTGATGGTTCACGTAGTGGGCCATTGGTATGGCTTTTTCCCCGTATCCCCCCAGGTGTCTGCAGGCTCAAAGAGCAGCGAGAAGCGTTCAGAGGAAAGCGATCCCGTGCCACCTTCCCCGTGCCCGGGCTGTCCCCGCACGCTGCCGGCTCGGGGATGCGGGGGGAGCGCCGGACCGGAGCGGAGCCCCGGGCGGCTCGCTGCTGCCCCCTAGCGGGGGAGGGACGTAATTACATCCCTGGGGGCTTTGGGGGGGGGCTGTCCCTGATAATTCTCTAGAAAGACTGGAGTTGCAGATCACGAGGGAAG

Receptor sequence

V5 tag

6x His

Puromycin resistance

Insulator sequence

pTR01F-hFGFR2IIIb delTV

CTAGTATTATGCCCAGTACATGACCTTATGGGACTTTCCTACTTGGCAGTACATCTACGTAAACTAGCAAAATAGGCTGTCCCCAGTGCAAGTGCAGGTGCCAGAACATTTCTCTGGCCTAACTGGCCGTAGTAATCAATTACGGGGTCATTAGTTCATAGCCCATATATGGAGTTCCGCGTTACATAACTTACGGTAAATGGCCCGCCTGGCTGACCGCCCAACGACCCCCGCCCATTGACGTCAATAATGACGTATGTTCCCATAGTAACGCCAATAGGGACTTTCCATTGACGTCAATGGGTGGAGTATTTACGGTAAACTGCCCACTTGGCAGTACATCAAGTGTATCATATGCCAAGTACGCCCCCTATTGACGTCAATGACGGTAAATGGCCCGCCTGGCATTATGCCCAGTACATGACCTTATGGGACTTTCCTACTTGGCAGTACATCTACGTATTAGTCATCGCTATTACCATGGTGATGCGGTTTTGGCAGTACATCAATGGGCGTGGATAGCGGTTTGACTCACGGGGATTTCCAAGTCTCCACCCCATTGACGTCAATGGGAGTTTGTTTTGGCACCAAAATCAACGGGACTTTCCAAAATGTCGTAACAACTCCGCCCCATTGACGCAAATGGGCGGTAGGCGTGTACGGTGGGAGGTCTATATAAGCAGAGCTCTCTGGCTAACTAGAGAACCCACTGCTTACTGGCTTATCGAAATTAATACGACTCACTATAGGGAGACCCAAGCTGGCTAGTTAAGCTTGGTACCGAGCTCGGATCCAGTACCCTTCACCATGGTCAGCTGGGGTCGTTTCATCTGCCTGGTCGTGGTCACCATGGCAACCTTGTCCCTGGCCCGGCCCTCCTTCAGTTTAGTTGAGGATACCACATTAGAGCCAGAAGAGCCACCAACCAAATACCAAATCTCTCAACCAGAAGTGTACGTGGCTGCGCCAGGGGAGTCGCTAGAGGTGCGCTGCCTGTTGAAAGATGCCGCCGTGATCAGTTGGACTAAGGATGGGGTGCACTTGGGGCCCAACAATAGGACAGTGCTTATTGGGGAGTACTTGCAGATAAAGGGCGCCACGCCTAGAGACTCCGGCCTCTATGCTTGTACTGCCAGTAGGACTGTAGACAGTGAAACTTGGTACTTCATGGTGAATGTCACAGATGCCATCTCATCCGGAGATGATGAGGATGACACCGATGGTGCGGAAGATTTTGTCAGTGAGAACAGTAACAACAAGAGAGCACCATACTGGACCAACACAGAAAAGATGGAAAAGCGGCTCCATGCTGTGCCTGCGGCCAACACTGTCAAGTTTCGCTGCCCAGCCGGGGGAAACCCAATGCCAACCATGCGGTGGCTGAAAAACGGGAAGGAGTTTAAGCAGGAGCATCGCATTGGAGGCTACAAGGTACGAAACCAGCACTGGAGCCTCATTATGGAAAGTGTGGTCCCATCTGACAAGGGAAATTATACCTGTGTGGTGGAGAATGAATACGGGTCCATCAATCACACGTACCACCTGGATGTTGTGGAGCGATCGCCTCACCGGCCCATCCTCCAAGCCGGACTGCCGGCAAATGCCTCCACAGTGGTCGGAGGAGACGTAGAGTTTGTCTGCAAGGTTTACAGTGATGCCCAGCCCCACATCCAGTGGATCAAGCACGTGGAAAAGAACGGCAGTAAATACGGGCCCGACGGGCTGCCCTACCTCAAGGTTCTCAAGCACTCGGGGATAAATAGTTCCAATGCAGAAGTGCTGGCTCTGTTCAATGTGACCGAGGCGGATGCTGGGGAATATATATGTAAGGTCTCCAATTATATAGGGCAGGCCAACCAGTCTGCCTGGCTCACTGTCCTGCCAAAACAGCAAGCGCCTGGAAGAGAAAAGGAGATTACAGCTTCCCCAGACTACCTGGAGATAGCCATTTACTGCATAGGGGTCTTCTTAATCGCCTGTATGGTGGTAACAGTCATCCTGTGCCGAATGAAGAACACGACCAAGAAGCCAGACTTCAGCAGCCAGCCGGCTGTGCACAAGCTGACCAAACGTATCCCCCTGCGGAGACAGGTATCGGCTGAGTCCAGCTCCTCCATGAACTCCAACACCCCGCTGGTGAGGATAACAACACGCCTCTCTTCAACGGCAGACACCCCCATGCTGGCAGGGGTCTCCGAGTATGAACTTCCAGAGGACCCAAAATGGGAGTTTCCAAGAGATAAGCTGACACTGGGCAAGCCCCTGGGAGAAGGTTGCTTTGGGCAAGTGGTCATGGCGGAAGCAGTGGGAATTGACAAAGACAAGCCCAAGGAGGCGGTCACCGTGGCCGTGAAGATGTTGAAAGATGATGCCACAGAGAAAGACCTTTCTGATCTGGTGTCAGAGATGGAGATGATGAAGATGATTGGGAAACACAAGAATATCATAAATCTTCTTGGAGCCTGCACACAGGATGGGCCTCTCTATGTCATAGTTGAGTATGCCTCTAAAGGCAACCTCCGAGAATACCTCCGAGCCCGGAGGCCACCCGGGATGGAGTACTCCTATGACATTAACCGTGTTCCTGAGGAGCAGATGACCTTCAAGGACTTGGTGTCATGCACCTACCAGCTGGCCAGAGGCATGGAGTACTTGGCTTCCCAAAAATGTATTCATCGAGATTTAGCAGCCAGAAATGTTTTGGTAACAGAAAACAATGTGATGAAAATAGCAGACTTTGGACTCGCCAGAGATATCAACAATATAGACTATTACAAAAAGACCACCAATGGGCGGCTTCCAGTCAAGTGGATGGCTCCAGAAGCCCTGTTTGATAGAGTATACACTCATCAGAGTGATGTCTGGTCCTTCGGGGTGTTAATGTGGGAGATCTTCACTTTAGGGGGCTCGCCCTACCCAGGGATTCCCGTGGAGGAACTTTTTAAGCTGCTGAAGGAAGGACACAGAATGGATAAGCCAGCCAACTGCACCAACGAACTGTACATGATGATGAGGGACTGTTGGCATGCAGTGCCCTCCCAGAGACCAACGTTCAAGCAGTTGGTAGAAGACTTGGATCGAATCCTCACTCTCACAACCAATGAGGAATACTTGGACCTCAGCCAACCTCTCGAACAGTATTCACCTAGTTACCCTGACACAAGAAGTTCTTGTTCTTCAGGAGATGATTCTGTTTTTTCTCCAGACCCCATGCCTTACGAACCATGCCTTCCTCAGTATCCACACATAAACGGCAGTGTTAAAACAGCGAAGGGTCAAGACAATTCTGCAGATATCCAGCACAGTGGCGGCCGCTCGAGTCTAGAGGGCCCGCGGTTCGAAGGTAAGCCTATCCCTAACCCTCTCCTCGGTCTCGATTCTACGCGTACCGGTCATCATCACCATCACCATTGAGTTTTAGAGTCGGGGCGGCCGGCCGCTTCGAGCAGACATGATAAGATACATTGATGAGTTTGGACAAACCACAACTAGAATGCAGTGAAAAAAATGCTTTATTTGTGAAATTTGTGATGCTATTGCTTTATTTGTAACCATTATAAGCTGCAATAAACAAGTTAACAACAACAATTGCATTCATTTTATGTTTCAGGTTCAGGGGGAGGTGTGGGAGGTTTTTTAAAGCAAGTAAAACCTCTACAAATGTGGTAAAATCGATAAGGATCCGTTTGCGTATTGGGCGCTCTTCCGCTGATCTGCGCAGCACCATGGCCTGAAATAACCTCTGAAAGAGGAACTTGGTTAGCTACCTTCTAAATCGGATCCGCGGCCGCAAGGATCTGCGATCGCTCCGGTGCCCGTCAGTG

Receptor sequence

V5 tag

6x His

pTR01F-hFGFR2IIIb WT

CTAGTATTATGCCCAGTACATGACCTTATGGGACTTTCCTACTTGGCAGTACATCTACGTAAACTAGCAAAATAGGCTGTCCCCAGTGCAAGTGCAGGTGCCAGAACATTTCTCTGGCCTAACTGGCCGTAGTAATCAATTACGGGGTCATTAGTTCATAGCCCATATATGGAGTTCCGCGTTACATAACTTACGGTAAATGGCCCGCCTGGCTGACCGCCCAACGACCCCCGCCCATTGACGTCAATAATGACGTATGTTCCCATAGTAACGCCAATAGGGACTTTCCATTGACGTCAATGGGTGGAGTATTTACGGTAAACTGCCCACTTGGCAGTACATCAAGTGTATCATATGCCAAGTACGCCCCCTATTGACGTCAATGACGGTAAATGGCCCGCCTGGCATTATGCCCAGTACATGACCTTATGGGACTTTCCTACTTGGCAGTACATCTACGTATTAGTCATCGCTATTACCATGGTGATGCGGTTTTGGCAGTACATCAATGGGCGTGGATAGCGGTTTGACTCACGGGGATTTCCAAGTCTCCACCCCATTGACGTCAATGGGAGTTTGTTTTGGCACCAAAATCAACGGGACTTTCCAAAATGTCGTAACAACTCCGCCCCATTGACGCAAATGGGCGGTAGGCGTGTACGGTGGGAGGTCTATATAAGCAGAGCTCTCTGGCTAACTAGAGAACCCACTGCTTACTGGCTTATCGAAATTAATACGACTCACTATAGGGAGACCCAAGCTGGCTAGTTAAGCTTGGTACCGAGCTCGGATCCAGTACCCTTCACCATGGTCAGCTGGGGTCGTTTCATCTGCCTGGTCGTGGTCACCATGGCAACCTTGTCCCTGGCCCGGCCCTCCTTCAGTTTAGTTGAGGATACCACATTAGAGCCAGAAGAGCCACCAACCAAATACCAAATCTCTCAACCAGAAGTGTACGTGGCTGCGCCAGGGGAGTCGCTAGAGGTGCGCTGCCTGTTGAAAGATGCCGCCGTGATCAGTTGGACTAAGGATGGGGTGCACTTGGGGCCCAACAATAGGACAGTGCTTATTGGGGAGTACTTGCAGATAAAGGGCGCCACGCCTAGAGACTCCGGCCTCTATGCTTGTACTGCCAGTAGGACTGTAGACAGTGAAACTTGGTACTTCATGGTGAATGTCACAGATGCCATCTCATCCGGAGATGATGAGGATGACACCGATGGTGCGGAAGATTTTGTCAGTGAGAACAGTAACAACAAGAGAGCACCATACTGGACCAACACAGAAAAGATGGAAAAGCGGCTCCATGCTGTGCCTGCGGCCAACACTGTCAAGTTTCGCTGCCCAGCCGGGGGGAACCCAATGCCAACCATGCGGTGGCTGAAAAACGGGAAGGAGTTTAAGCAGGAGCATCGCATTGGAGGCTACAAGGTACGAAACCAGCACTGGAGCCTCATTATGGAAAGTGTGGTCCCATCTGACAAGGGAAATTATACCTGTGTAGTGGAGAATGAATACGGGTCCATCAATCACACGTACCACCTGGATGTTGTGGAGCGATCGCCTCACCGGCCCATCCTCCAAGCCGGACTGCCGGCAAATGCCTCCACAGTGGTCGGAGGAGACGTAGAGTTTGTCTGCAAGGTTTACAGTGATGCCCAGCCCCACATCCAGTGGATCAAGCACGTGGAAAAGAACGGCAGTAAATACGGGCCCGACGGGCTGCCCTACCTCAAGGTTCTCAAGCACTCGGGGATAAATAGTTCCAATGCAGAAGTGCTGGCTCTGTTCAATGTGACCGAGGCGGATGCTGGGGAATATATATGTAAGGTCTCCAATTATATAGGGCAGGCCAACCAGTCTGCCTGGCTCACTGTCCTGCCAAAACAGCAAGCGCCTGGAAGAGAAAAGGAGATTACAGCTTCCCCAGACTACCTGGAGATAGCCATTTACTGCATAGGGGTCTTCTTAATCGCCTGTATGGTGGTAACAGTCATCCTGTGCCGAATGAAGAACACGACCAAGAAGCCAGACTTCAGCAGCCAGCCGGCTGTGCACAAGCTGACCAAACGTATCCCCCTGCGGAGACAGGTAACAGTTTCGGCTGAGTCCAGCTCCTCCATGAACTCCAACACCCCGCTGGTGAGGATAACAACACGCCTCTCTTCAACGGCAGACACCCCCATGCTGGCAGGGGTCTCCGAGTATGAACTTCCAGAGGACCCAAAATGGGAGTTTCCAAGAGATAAGCTGACACTGGGCAAGCCCCTGGGAGAAGGTTGCTTTGGGCAAGTGGTCATGGCGGAAGCAGTGGGAATTGACAAAGACAAGCCCAAGGAGGCGGTCACCGTGGCCGTGAAGATGTTGAAAGATGATGCCACAGAGAAAGACCTTTCTGATCTGGTGTCAGAGATGGAGATGATGAAGATGATTGGGAAACACAAGAATATCATAAATCTTCTTGGAGCCTGCACACAGGATGGGCCTCTCTATGTCATAGTTGAGTATGCCTCTAAAGGCAACCTCCGAGAATACCTCCGAGCCCGGAGGCCACCCGGGATGGAGTACTCCTATGACATTAACCGTGTTCCTGAGGAGCAGATGACCTTCAAGGACTTGGTGTCATGCACCTACCAGCTGGCCAGAGGCATGGAGTACTTGGCTTCCCAAAAATGTATTCATCGAGATTTAGCAGCCAGAAATGTTTTGGTAACAGAAAACAATGTGATGAAAATAGCAGACTTTGGACTCGCCAGAGATATCAACAATATAGACTATTACAAAAAGACCACCAATGGGCGGCTTCCAGTCAAGTGGATGGCTCCAGAAGCCCTGTTTGATAGAGTATACACTCATCAGAGTGATGTCTGGTCCTTCGGGGTGTTAATGTGGGAGATCTTCACTTTAGGGGGCTCGCCCTACCCAGGGATTCCCGTGGAGGAACTTTTTAAGCTGCTGAAGGAAGGACACAGAATGGATAAGCCAGCCAACTGCACCAACGAACTGTACATGATGATGAGGGACTGTTGGCATGCAGTGCCCTCCCAGAGACCAACGTTCAAGCAGTTGGTAGAAGACTTGGATCGAATcCTCACTCTCACAACCAATGAGGAATACTTGGACCTCAGCCAACCTCTCGAACAGTATTCACCTAGTTACCCTGACACAAGAAGTTCTTGTTCTTCAGGAGATGATTCTGTTTTTTCTCCAGACCCCATGCCTTACGAACCATGCCTTCCTCAGTATCCACACATAAACGGCAGTGTTAAAACAGCGAAGGGTCAAGACAATTCTGCAGATATCCAGCACAGTGGCGGCCGCTCGAGTCTAGAGGGCCCGCGGTTCGAAGGTAAGCCTATCCCTAACCCTCTCCTCGGTCTCGATTCTACGCGTACCGGTCATCATCACCATCACCATTGAGTTTTAGAGTCGGGGCGGCCGGCCGCTTCGAGCAGACATGATAAGATACATTGATGAGTTTGGACAAACCACAACTAGAATGCAGTGAAAAAAATGCTTTATTTGTGAAATTTGTGATGCTATTGCTTTATTTGTAACCATTATAAGCTGCAATAAACAAGTTAACAACAACAATTGCATTCATTTTATGTTTCAGGTTCAGGGGGAGGTGTGGGAGGTTTTTTAAAGCAAGTAAAACCTCTACAAATGTGGTAAAATCGATAAGGATCCGTTTGCGTATTGGGCGCTCTTCCGCTGATCTGCGCAGCACCATGGCCTGAAATAACCTCTGAAAGAGGAACTTGGTTAGCTACCTTCTAAATCGGATCCGCGGCCGCAAGGATCTGCGATCGCTCCGGTGCCCGTCAGTG

Receptor sequence

V5 tag

6x His
