# Supplementary figures and images for: FGFR2 residence in primary cilia is necessary for epithelial cell signaling
Source: J Cell Biol. 2025 Apr 22;224(7):e202311030. doi: 10.1083/jcb.202311030 (PMC12010920; doi:10.1083/jcb.202311030)

C

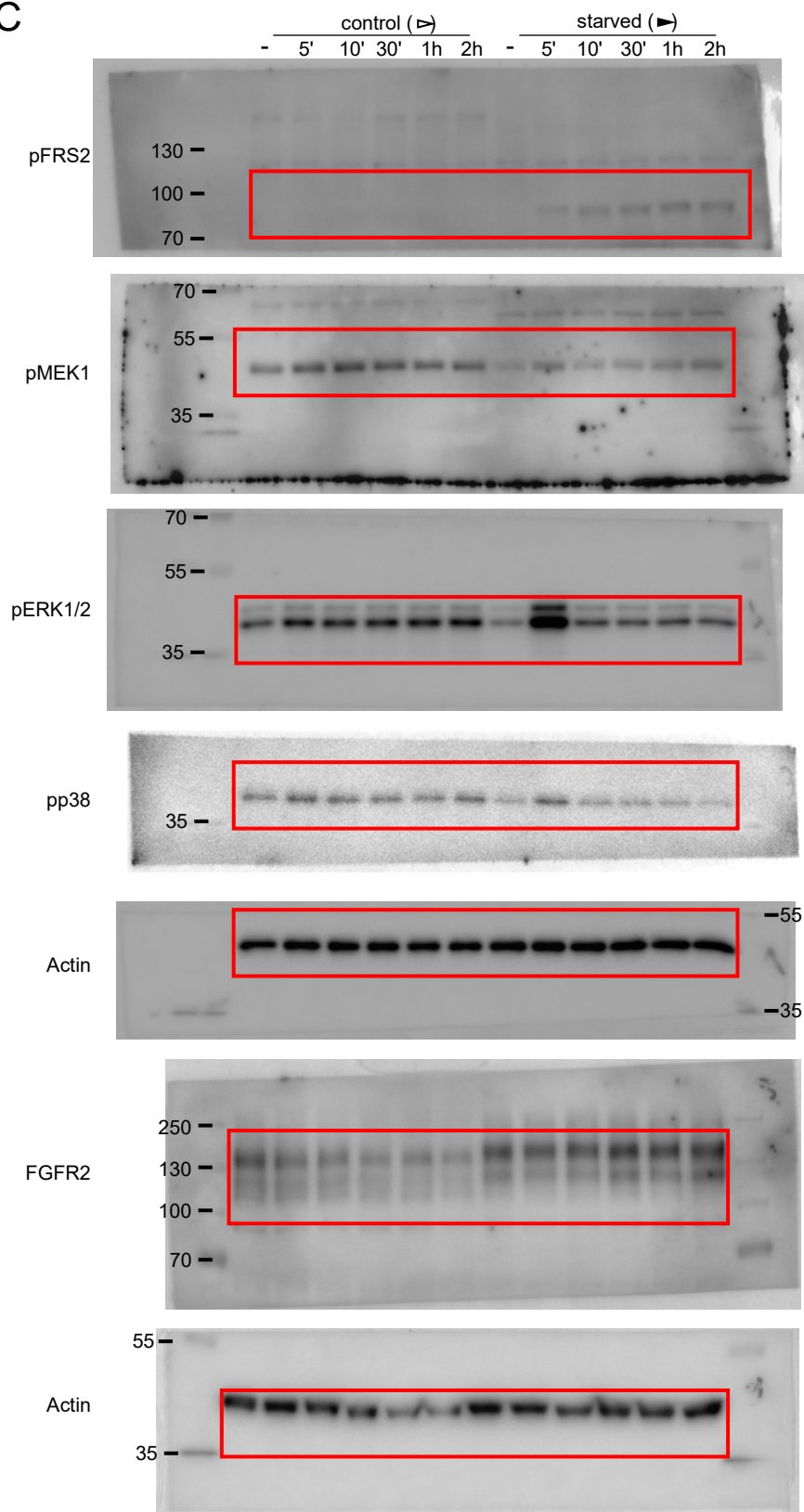

F

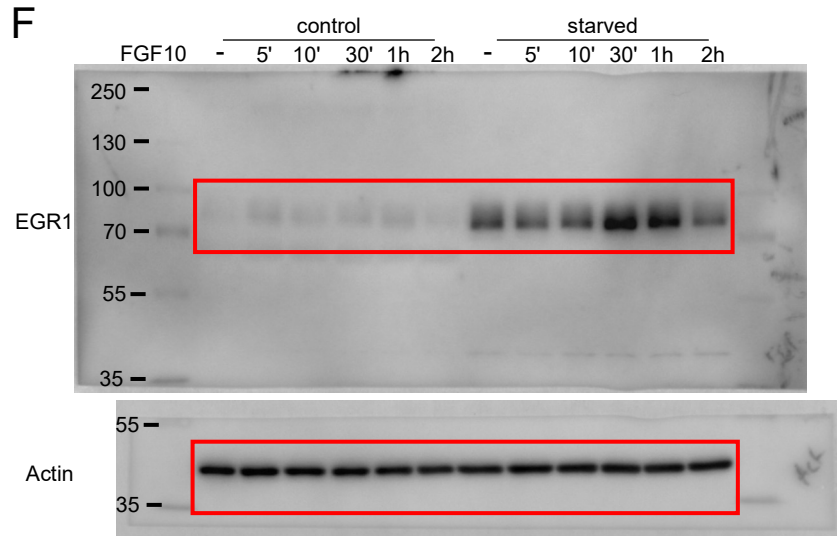

Supplement: SourceData F2 — is the source file for Fig. 2. [file jcb_202311030_sourcedataf2.pdf]

**B**

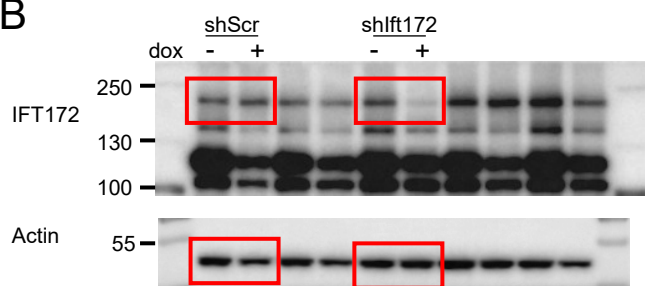

E

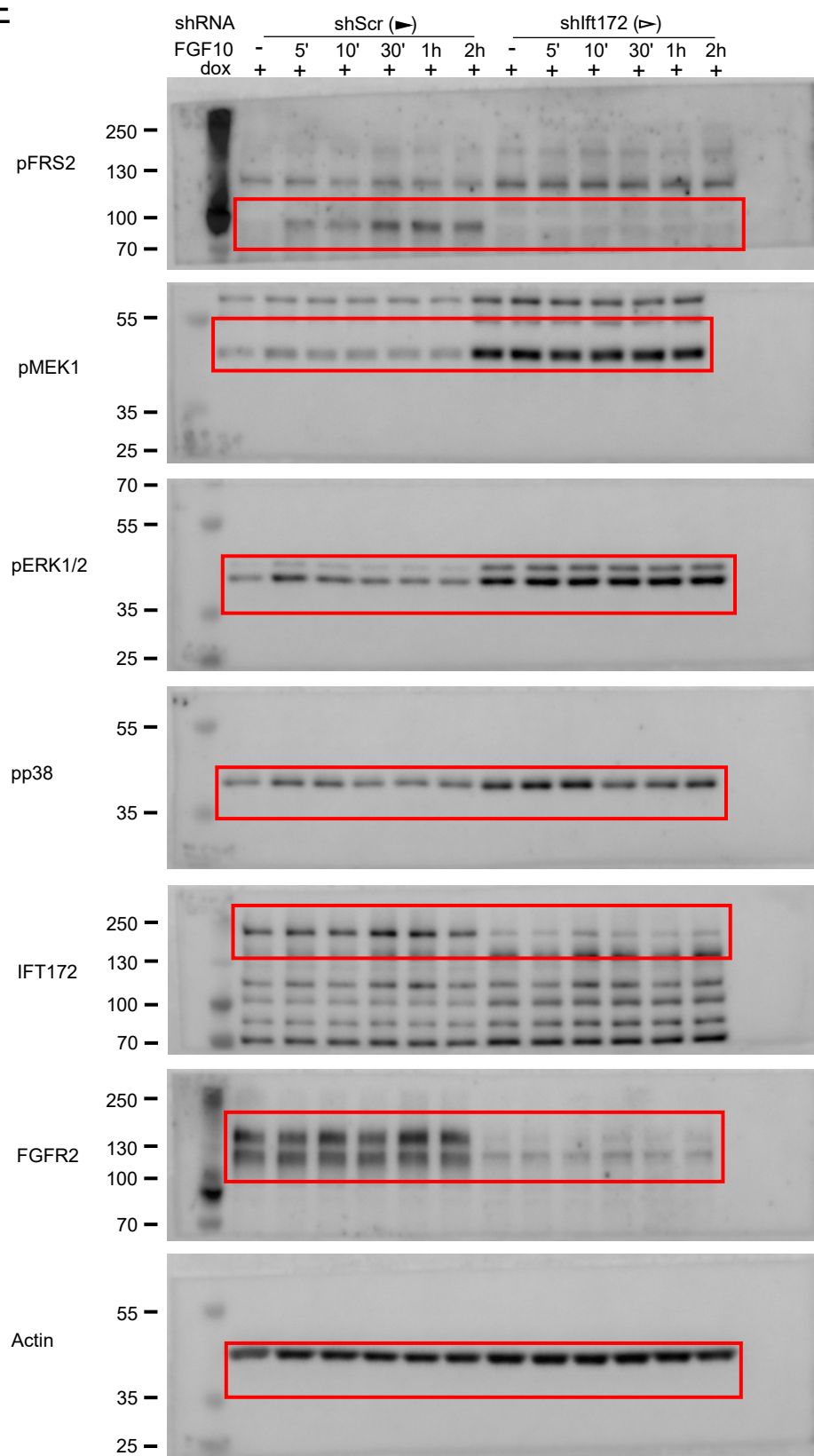

K

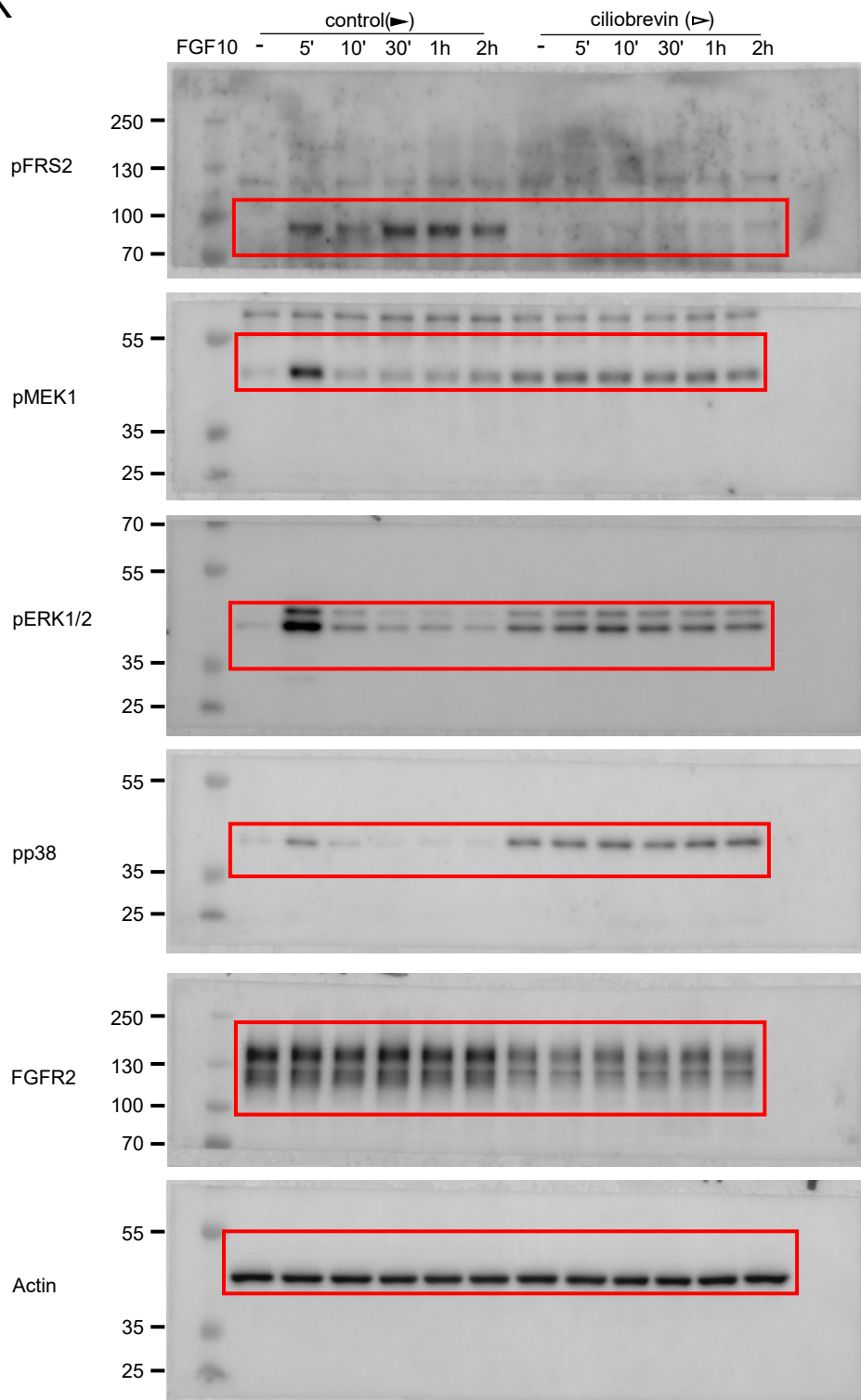

Supplement: SourceData F3 — is the source file for Fig. 3. [file jcb_202311030_sourcedataf3.pdf]

G

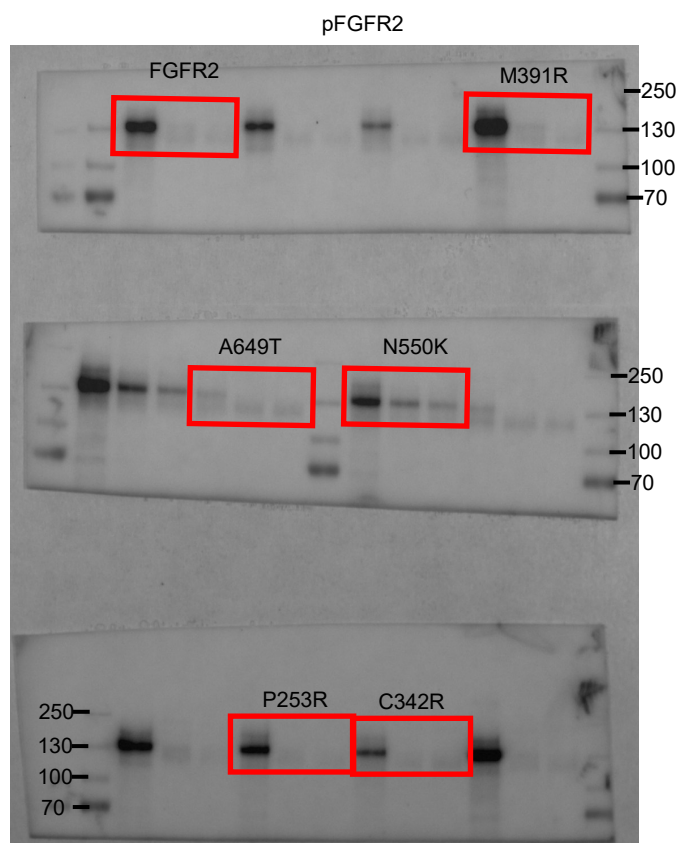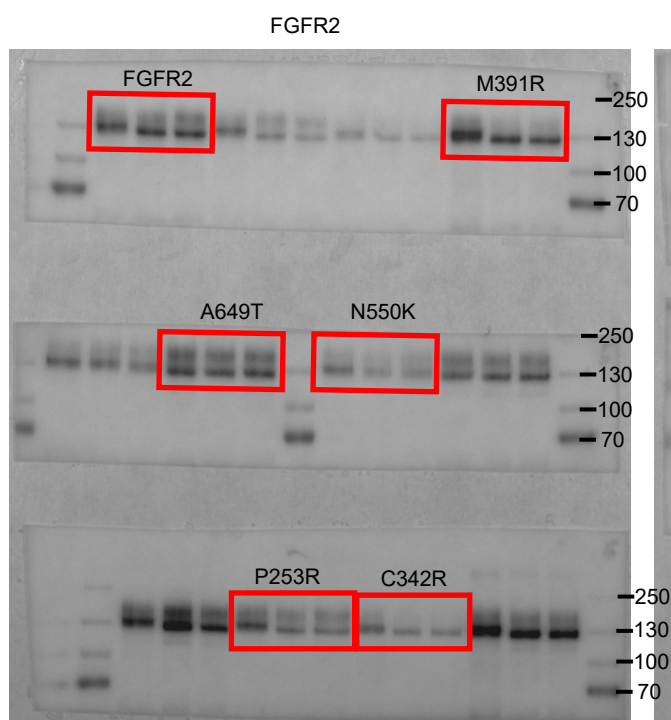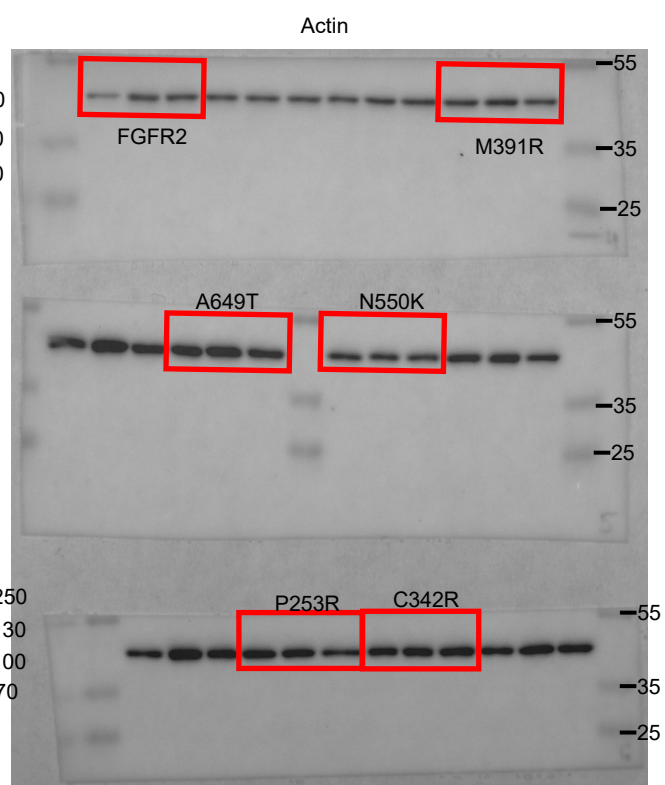

Supplement: SourceData F5 — is the source file for Fig. 5. [file jcb_202311030_sourcedataf5.pdf]

C

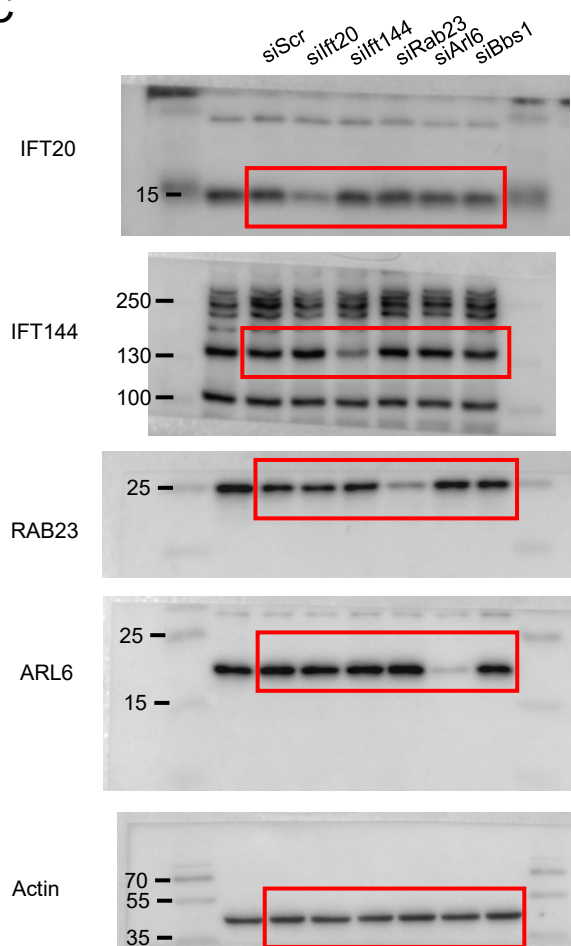

Supplement: SourceData F6 — is the source file for Fig. 6. [file jcb_202311030_sourcedataf6.pdf]

## G

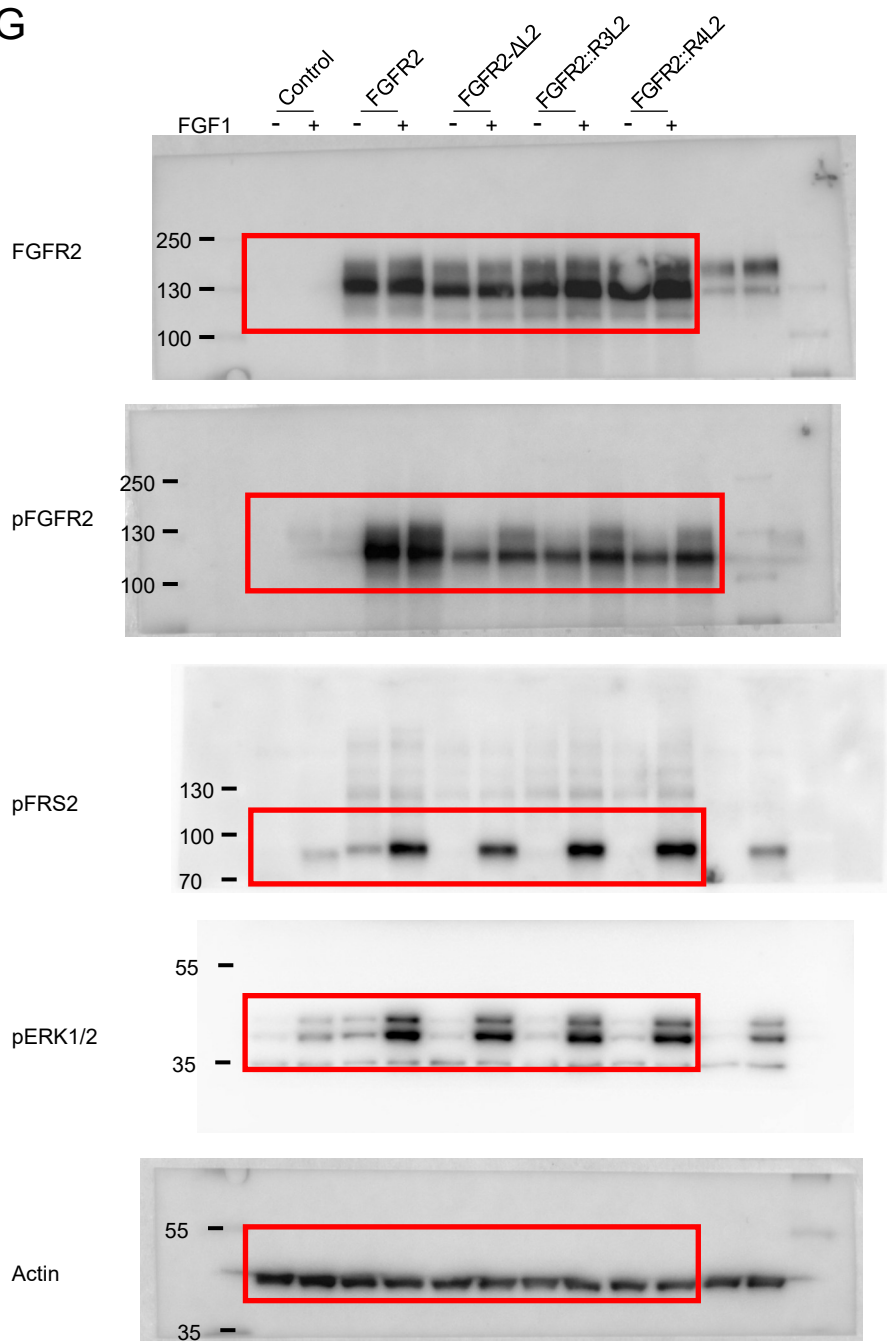

Supplement: SourceData F7 — is the source file for Fig. 7. [file jcb_202311030_sourcedataf7.pdf]

D

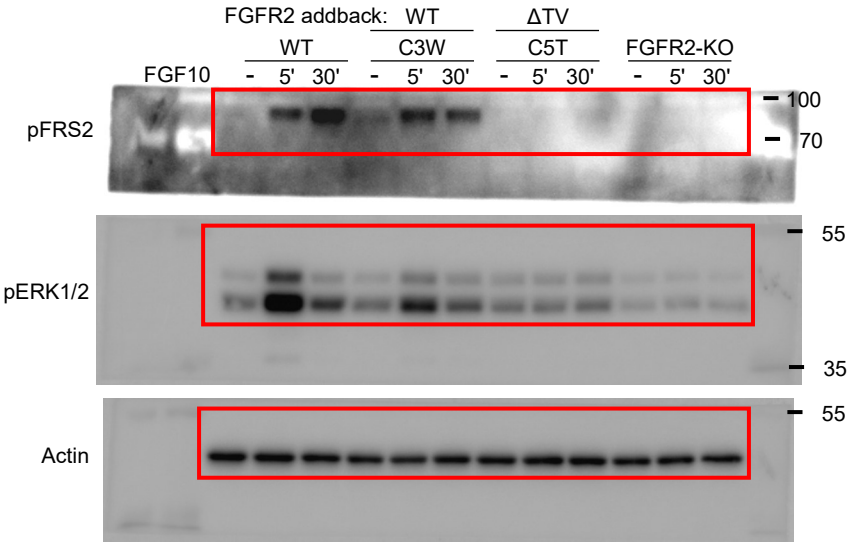

F

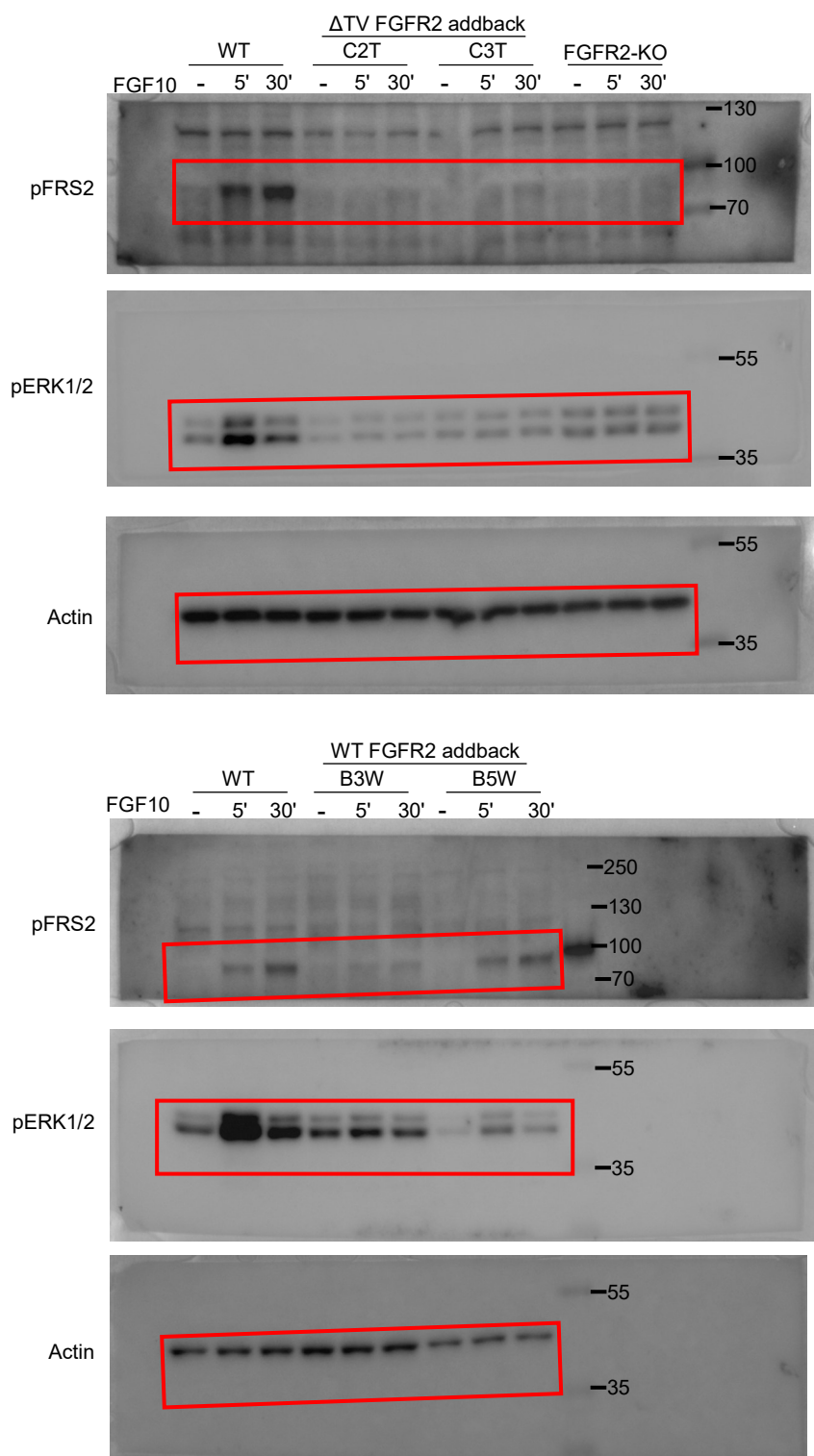

Supplement: SourceData F8 — is the source file for Fig. 8. [file jcb_202311030_sourcedataf8.pdf]

A

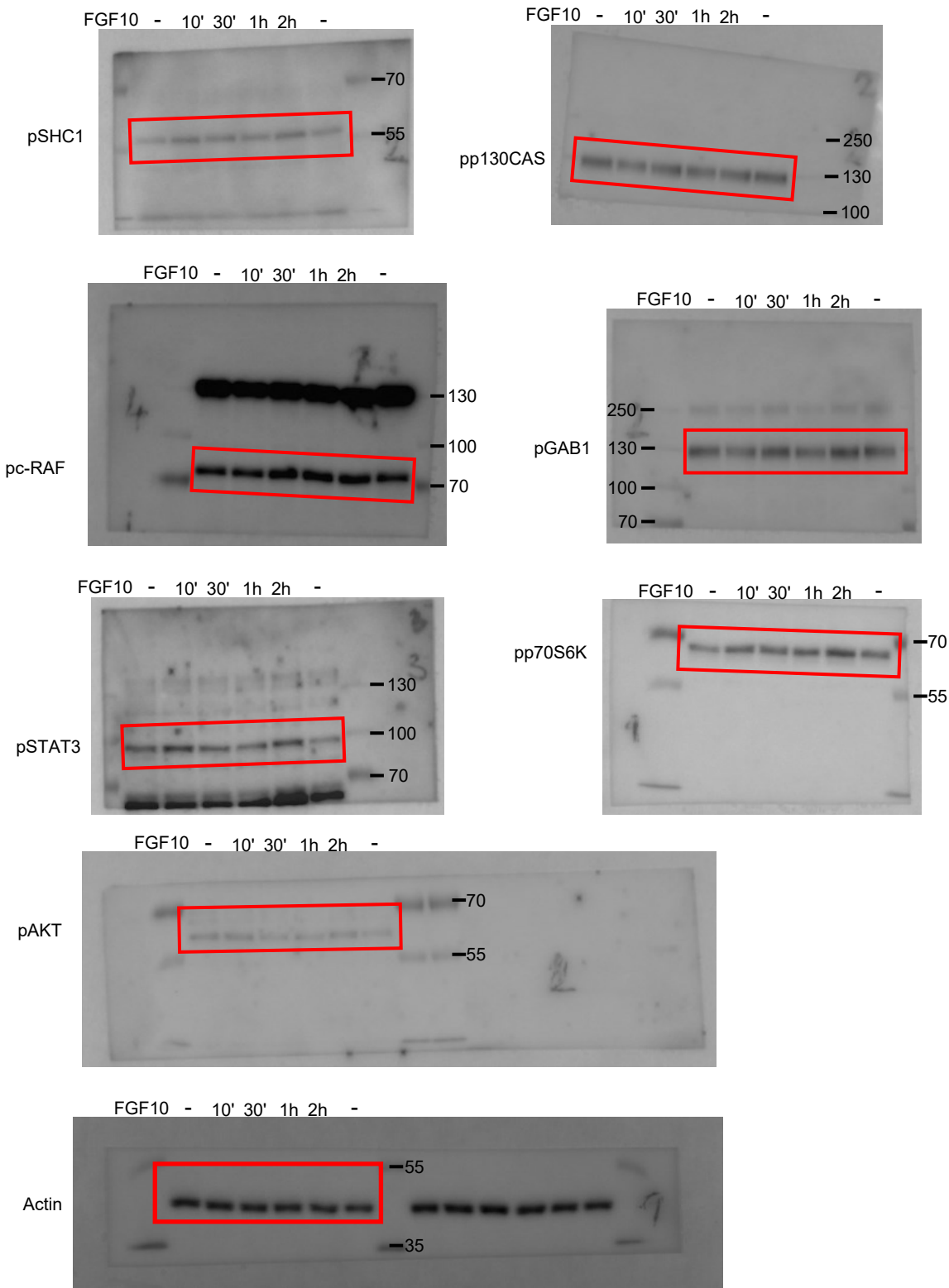

C

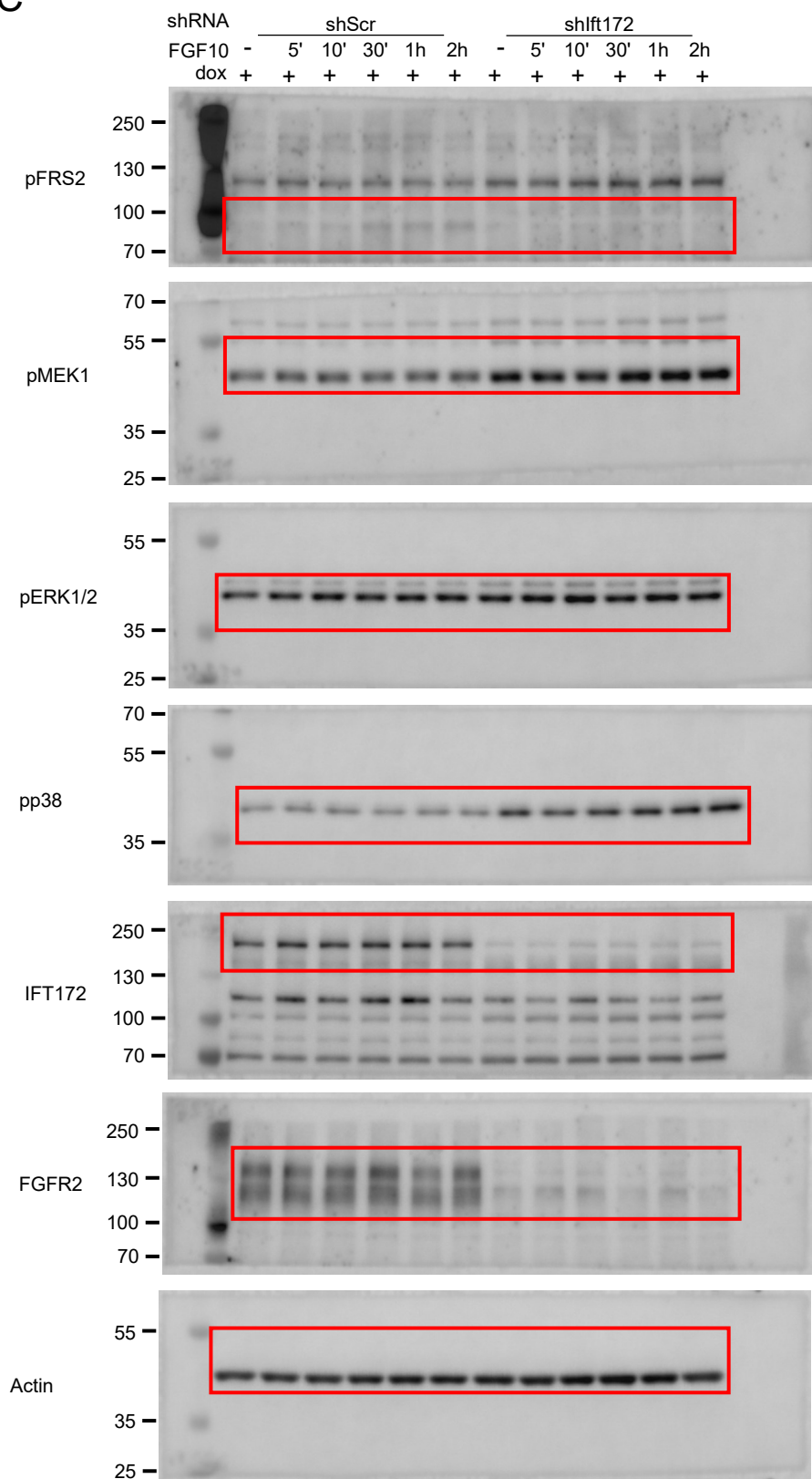

F

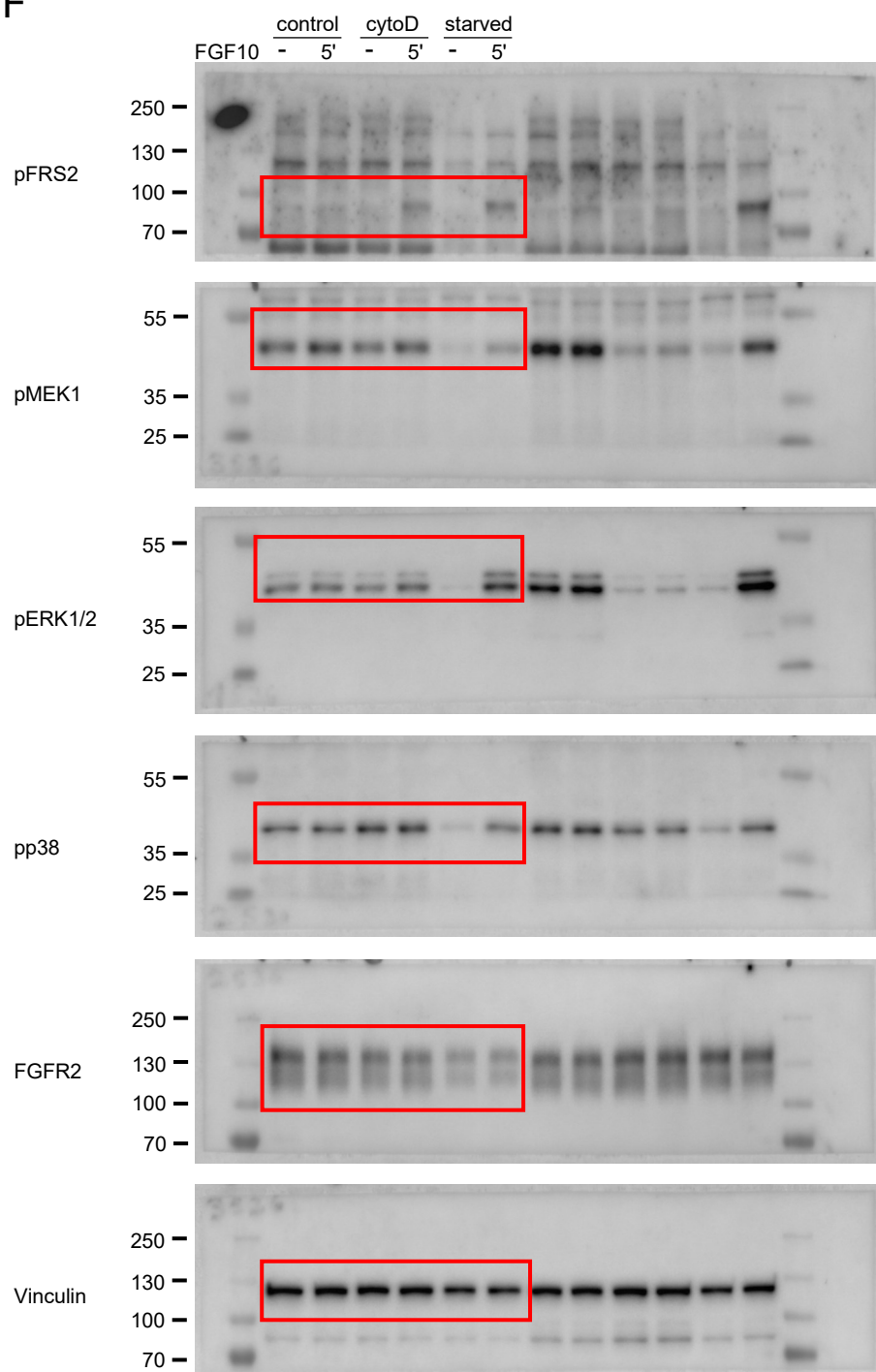

Supplement: SourceData FS2 — is the source file for Fig. S2. [file jcb_202311030_sourcedatafs2.pdf]

**B**

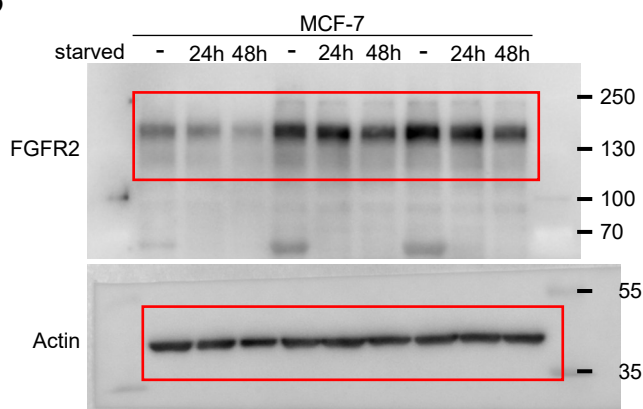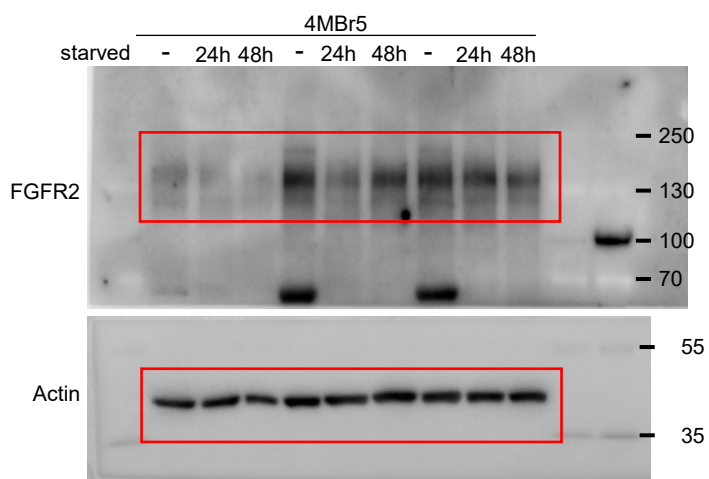

**C**

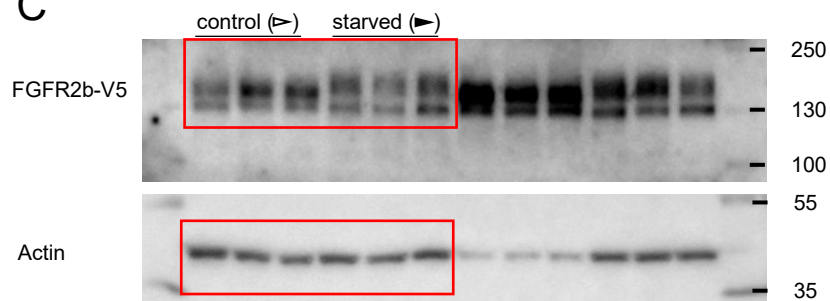

**E**

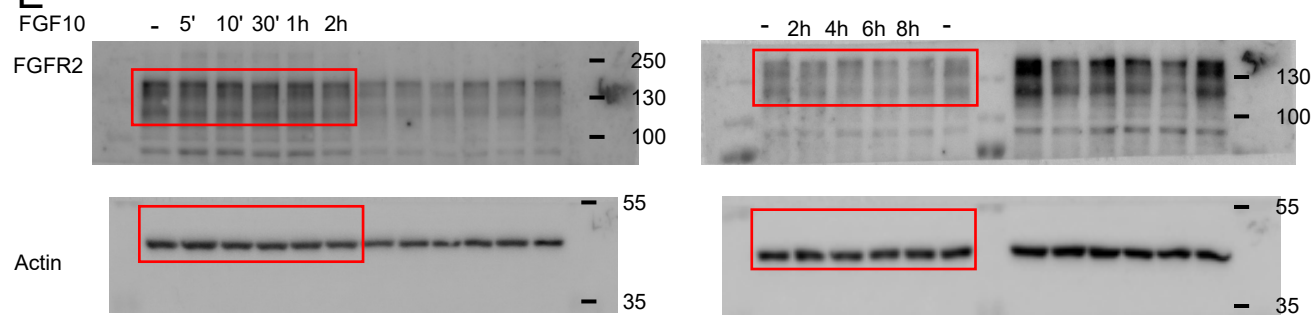

Supplement: SourceData FS3 — is the source file for Fig. S3. [file jcb_202311030_sourcedatafs3.pdf]

A

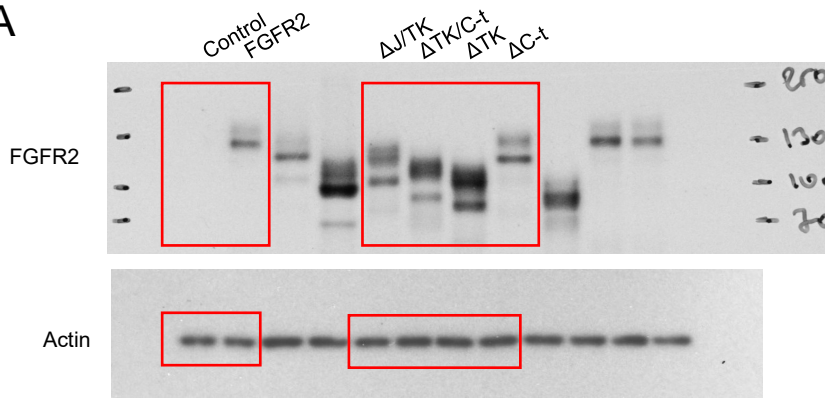

C

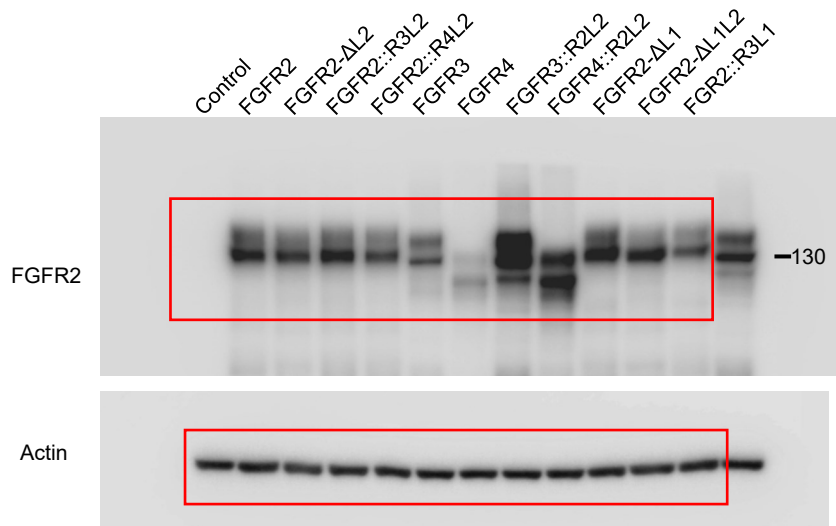

E

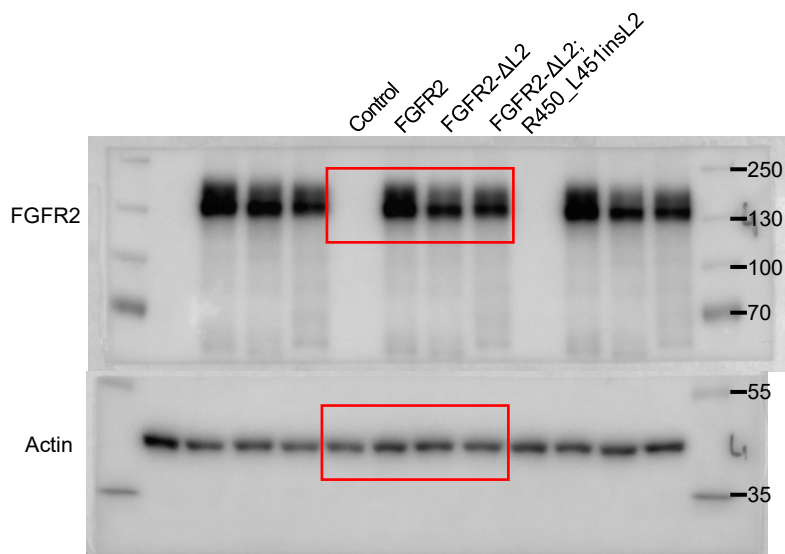

G

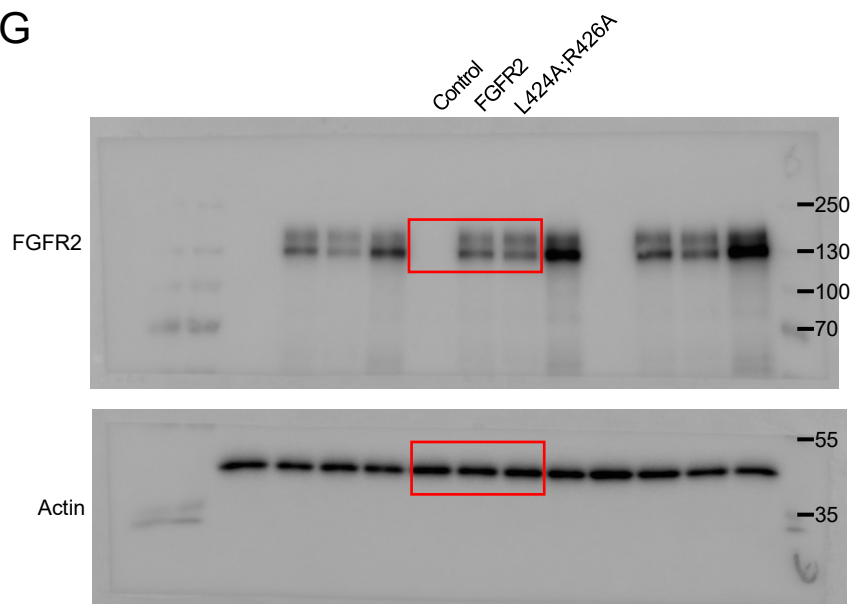

I

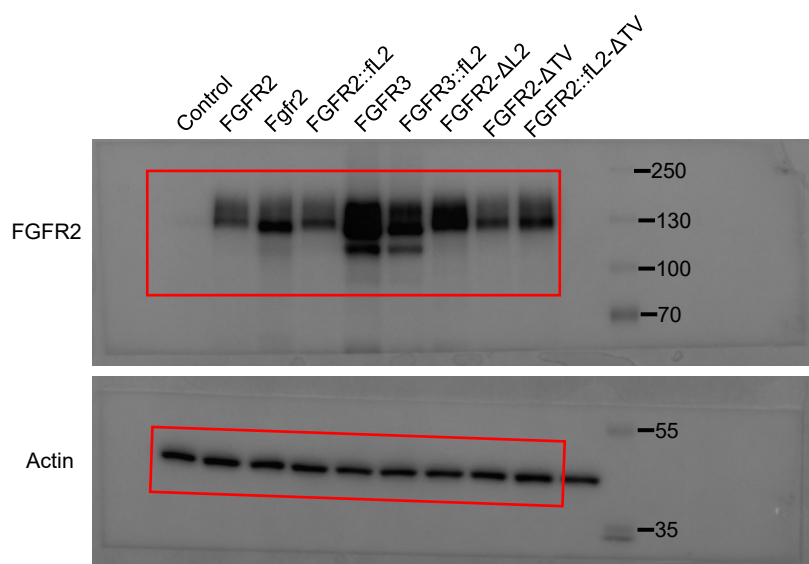

Supplement: SourceData FS6 — is the source file for Fig. S6. [file jcb_202311030_sourcedatafs6.pdf]
